# Stereodivergent Anion Binding Catalysis with Molecular Motors

**DOI:** 10.1002/anie.201913054

**Published:** 2019-12-12

**Authors:** Ruth Dorel, Ben L. Feringa

**Affiliations:** ^1^ Stratingh Institute for Chemistry Zernike Institute for Advanced Materials University of Groningen Nijenborgh 4 9747AG Groningen The Netherlands

**Keywords:** anion binding catalysis, molecular motors, oligotriazoles, photoswitches, stereodivergent catalysis

## Abstract

A photoresponsive chiral catalyst based on an oligotriazole‐functionalized unidirectional molecular motor has been developed for stereodivergent anion binding catalysis. The motor function controls the helical chirality of supramolecular assemblies with chloride anions, which by means of chirality transfer enables the enantioselective addition of a silyl ketene acetal nucleophile to oxocarbenium cations. Reversal of stereoselectivity (up to 142 % Δ*ee*) was achieved through rotation of the motor core induced by photochemical and thermal isomerization steps.

## Graphical abstract

**Motorized catalysis**: A photoresponsive chiral catalyst based on an oligotriazole‐functionalized unidirectional molecular motor has been developed for stereodivergent anion binding catalysis. The motor function controls the helical chirality of supramolecular assemblies with chloride anions.

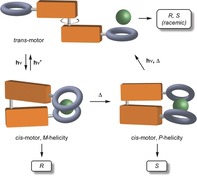

The allosteric regulation of enzymes in nature^1^ has served as a source of inspiration for the development of responsive artificial catalysts whose function can be altered by the action of external stimuli,^2^, ^3^ which may translate into variations in activity,2a, 4 selectivity,^5^ or reaction type^6^ without reengineering the structure of the catalyst. Particularly desirable and at the same time remarkably challenging are switchable catalysts that can on‐demand selectively provide all the different stereoisomers in a given chemical transformation.^7^ Our group pioneered this concept^8^ through the use of so‐called first generation molecular motors^9^—which can be interconverted between four different states by the action of light and heat—containing two reactive sites attached to the motor core. Based on this approach, the stereodivergent addition of a thiolate to an α,β‐unsaturated ketone was initially accomplished,^8^ followed by the development of thiourea‐based switchable organocatalysts for the Henry reaction^10^ as well as a Trost‐type ligand for a palladium‐catalyzed asymmetric allylic substitution.^11^ More recently, photoswitchable catalysts based on second generation molecular motors^12^ have also been realized.^13^

Asymmetric anion binding catalysis is based on the activation of an ion pair for the attack of a nucleophile through the recognition of the anion by a chiral receptor.^14^ Since the groundbreaking reports by Jacobsen and co‐workers on the use of chiral thioureas as the anion binding catalysts,^15^ several other classes of anion receptors have been designed and successfully applied in asymmetric catalysis.^16^ Herein, we report on the use of a molecular motor‐based stimuli‐responsive anion receptor to illustrate the concept of adaptive stereodivergent anion binding catalysis, which is achieved by means of supramolecular transfer of chirality (Figure 1). The precise positioning of two ion binders with respect to each other at each stage of the rotation cycle of the motor creates different chiral environments at the anion recognition site, which translates into different stereochemical outcomes in anion binding catalysis.

**Figure 1:**
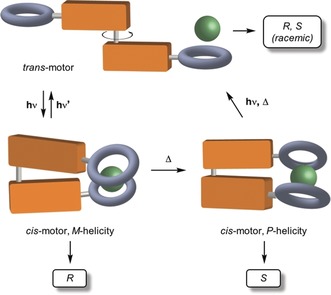
The use of molecular motors for stereodivergent anion binding catalysis.

Triazoles have been recognized as amide surrogate anion binders due to their dipole moment (ca. 5 D), which results in an electropositive C−H capable of engaging in hydrogen bonding with negatively charged species.^17^ In the case of aryl triazole oligomers that cannot adopt a planar conformation, this interaction translates into a helical supramolecular arrangement in the presence of, among others, chloride anions.^18^, ^19^ Based on this concept, chiral oligotriazoles bearing a *trans*‐1,2‐cyclohexanediamine backbone have been developed^20^ and their utility in asymmetric anion binding catalysis has been elegantly illustrated for the dearomatization of a variety of heterocycles.^21^ We anticipated that a first generation molecular motor could serve as a unique responsive chiral scaffold for oligotriazole‐based anion receptors, which due to the motor function could interconvert between isomeric states as a response to external stimuli. In our design, molecular motors **1** feature two branches, each of them containing two triazole moieties linked by an aryl group (Scheme 1). While the two branches would be far apart from each other in (*R*,*R*)‐(*P*,*P*)‐*trans*‐**1**, they could come into close proximity in both (*R*,*R*)‐(*M*,*M*)‐*cis*‐**1** and (*R*,*R*)‐(*P*,*P*)‐*cis*‐**1** forms, which would be sequentially accessed by irradiation of (*R*,*R*)‐(*P*,*P*)‐*trans*‐**1** and heating of the resulting photogenerated species, respectively. These species are pseudoenantiomers, only differing from each other in the handedness of the helical structure. In the presence of chloride anions, (*R*,*R*)‐(*M*,*M*)‐*cis*‐**1** and (*R*,*R*)‐(*P*,*P*)‐*cis*‐**1** can form a supramolecular helical assembly to give rise to (*R*,*R*)‐(*M*,*M*)‐*cis*‐**1‐Cl** and (*R*,*R*)‐(*P*,*P*)‐*cis*‐**1‐Cl**, respectively, whose configuration would be dictated by the helicity of the motor backbone.^22^ We envisioned that this transfer and amplification of chiral information from the molecular to the supramolecular level could be exploited to achieve the preferential formation of different stereoisomers in the context of asymmetric anion binding catalysis depending upon the external stimulus applied.

**Scheme 1:**
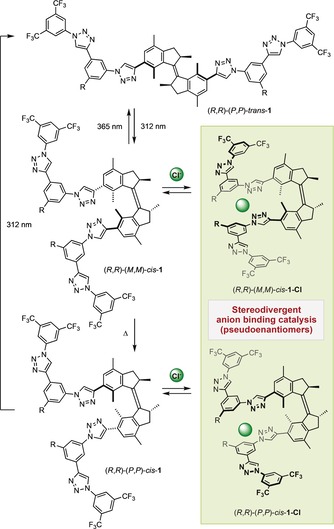
Oligotriazole‐functionalized molecular motors for stereodivergent anion binding catalysis.

Our starting point for the synthesis of the target triazole‐functionalized molecular motors was a mixture of (*R*, *R*)‐(*P*,*P*)‐*cis*‐ and (*R*, *R*)‐(*P*,*P*)‐*trans*‐**2** (e.r. >95:5), which results from the McMurry coupling of the corresponding enantiomerically enriched indanone.^23^ This mixture could be converted into pure (*R*, *R*)‐(*P*,*P*)‐*cis*‐**2** by heating at 180 °C (Scheme 2).^24^ While the attempts of direct Sonogashira coupling from (*R*, *R*)‐(*P*,*P*)‐*cis*‐**2** turned out to be unsuccessful, the double aromatic Finkelstein reaction^25^ proceeded smoothly on (*R*, *R*)‐(*P*,*P*)‐*cis*‐**2** to afford (*R*, *R*)‐(*P*,*P*)‐*cis*‐**3**, which could be coupled with trimethylsilylacetylene under standard reaction conditions. Subsequent cleavage of the trimethylsilyl groups at the alkyne termini provided (*R*, *R*)‐(*P*,*P*)‐*cis*‐**4**, which was obtained in its enantiomerically pure form after recrystallization. Alternatively, the double aromatic Finkelstein followed by double Sonogashira coupling starting from the mixture of *cis*‐ and *trans*‐**2** provided separable mixtures of bis‐alkynylated motors, which after TMS cleavage afforded (*R*, *R*)‐(*P*,*P*)‐*cis*‐ and (*R*, *R*)‐(*P*,*P*)‐*trans*‐**4**. Ultimately, the reaction of (*R*, *R*)‐(*P*,*P*)‐*cis*‐**4** with aromatic azides **5** and **6** in the presence of substoichiometric amounts of copper afforded tetra‐ and bistriazole‐functionalized molecular motors (*R*, *R*)‐(*P*,*P*)‐*cis*‐**1 a**–**d** and (*R*, *R*)‐(*P*,*P*)‐*cis*‐**7**, respectively. In the case of *cis*‐**1 a**–**d**, different electron withdrawing substituents (R) at the ring connecting the triazole moieties were selected, since they are known to have an impact on the performance of oligotriazole receptors in asymmetric catalysis.^20^ Likewise, (*R*, *R*)‐(*P*,*P*)‐*trans*‐**4** could be used to obtain *trans*‐configured oligotriazole‐containing molecular motors **1**.

**Scheme 2:**
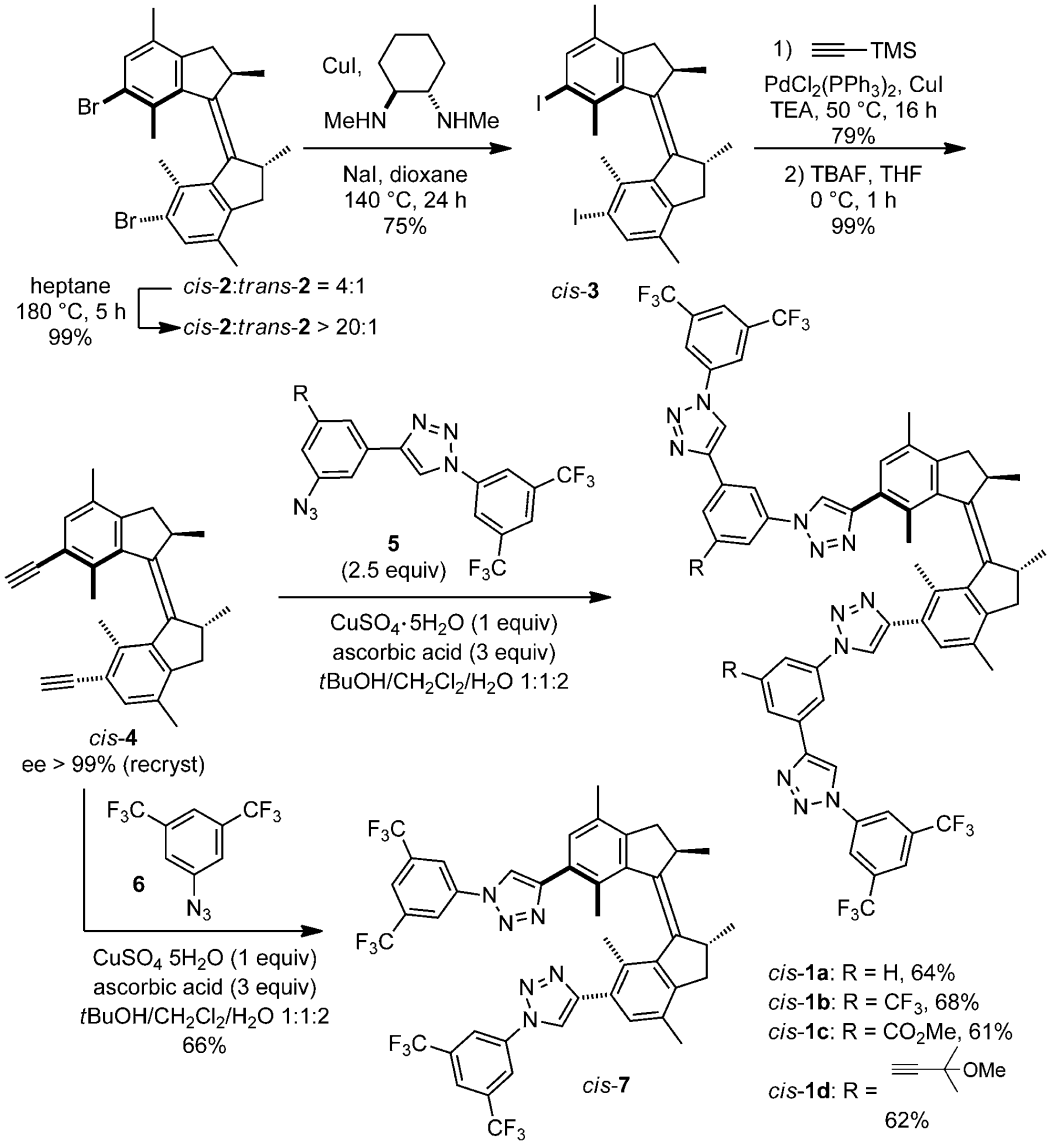
Synthesis of oligotriazole‐functionalized motors (*R*,*R*)‐(*P*,*P*)*‐cis*‐**1 a**–**d** and (*R*,*R*)‐(*P*,*P*)‐*cis*‐**7**. TEA=triethylamine. TBAF=tetrabutylammonium fluoride. THF=tetrahydrofuran.

With enantiomerically pure (*P*,*P*)‐*cis*‐configured triazole‐containing motors **1 a**–**d** and **7** in hand, their performance as anion binding catalysts was next examined. We selected the addition of silyl ketene acetal nucleophiles to 1‐chloroisochroman as a model reaction (Table 1), which has been studied in the presence of thiourea‐based anion binders^15^, ^26^ among others.^27^ We initially tested the reaction between 1‐chloroisochroman (**8**) and *tert*‐butyl((1‐isopropoxyvinyl)oxy)dimethylsilane (**9**) in MTBE at −70 °C for 36 h. We found that two triazole moieties per branch are required in order to induce enantioselectivity in this transformation since (*R*,*R*)‐(*P*,*P*)‐*cis*‐**7** provided *rac*‐**10** (Table 1, entry 1), which is in line with the predicted formation of a supramolecular helical structure upon chloride binding. Among the tetratriazole‐motors (*R*,*R*)‐(*P*,*P*)‐*cis*‐**1 a**–**d** tested (Table 1, entries 2–5), motor (*R*,*R*)‐(*P*,*P*)‐*cis*‐**1 b** bearing CF_3_ substituents at the linking ring gave the highest conversion (65 %) and enantioinduction (e.r.=86:14) in the formation of **10 a** (Table 1, entry 3) and was therefore selected for further optimization studies. The use of other solvents including Et_2_O, THF, CH_2_Cl_2_, or toluene resulted in lower values of enantioselectivity (see Table S1 in the Supporting Information for details). We also examined the influence of the nucleophile, finding lower levels of enantioinduction with the other silyl ketene acetals tested and no conversion when an enol ether was used as the nucleophile (see Table S2 in the Supporting Information for details). Therefore, **9** was the nucleophile of choice for further experimentation. Lowering the reaction temperature to −80 °C led to an increase in e.r. (Table 1, entry 6). Finally, dilution to an initial 0.15 m concentration of **8 a** provided the addition product **10 a** with 90:10 e.r. (Table 1, entry 7), which was isolated in 81 % yield after extending the reaction time to 5 days (Table 1, entry 8). The enantiomer preferentially formed in the presence of (*R*,*R*)‐(*P*,*P*)‐*cis*‐**1 b** as the catalyst was determined to be (*S*)‐**10 a** by comparison to previously published data (see Supporting Information for details).^15^

**Table 1:** Catalytic activity of motors (*R*,*R*)‐(*P*,*P*)‐*cis*‐**1 a**–**d** and (*R*,*R*)‐(*P*,*P*)‐*cis*‐**7** in the addition of silyl ketene acetal **9** to 1‐chloroisochroman **8**. 
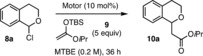

Entry	Motor	*T* [°C]	conversion [%]^[a]^	e.r.^[b]^
1	(*R*,*R*)‐(*P*,*P*)‐*cis*‐**7**	−70	20	50:50
2	(*R*,*R*)‐(*P*,*P*)‐*cis*‐**1 a**	−70	17	81:19
3	(*R*,*R*)‐(*P*,*P*)‐*cis*‐**1 b**	−70	65	86:14
4	(*R*,*R*)‐(*P*,*P*)‐*cis*‐**1 c**	−70	31	84:16
5	(*R*,*R*)‐(*P*,*P*)‐*cis*‐**1 d**	−70	47	80:20
6	(*R*,*R*)‐(*P*,*P*)‐*cis*‐**1 b**	−80	46	88:12
7^[c]^	(*R*,*R*)‐(*P*,*P*)‐*cis*‐**1 b**	−80	40	90:10
8^[c,d]^	(*R*,*R*)‐(*P*,*P*)‐*cis*‐**1 b**	−80	91(81)^[e]^	90:10

[a] Determined by ^1^H NMR. [b] Determined by chiral HPLC. [c] [**8 a**]=0.15 M. [d] Reaction time=5 days. [e] Isolated yield in parentheses. TBS=*tert*‐butyldimethylsilyl. MTBE=methyl *tert*‐butyl ether.

Having established **1 b** as the most efficient catalyst for the formation of **10 a**, its photoswitching behavior was next examined by UV/Vis spectroscopy (Figure 2). Irradiation of a solution of (*R*,*R*)‐(*P*,*P*)‐*trans*‐**1 b** in THF (312 nm) promoted its isomerization to (*R*,*R*)‐(*M*,*M*)‐*cis*‐**1 b** (Figure 2 a). The presence of clear isosbestic points is indicative of a unimolecular process (see Figure S1). This process could be reversed by irradiation with 365 nm light. Subsequent heating of (*R*,*R*)‐(*M*,*M*)‐*cis*‐**1 b** at 60 °C induced thermal helix inversion (THI) to form (*R*,*R*)‐(*P*,*P*)‐*cis*‐**1 b**. The same process was monitored by ^1^H NMR (Figure 2 b). Thus, a PSS ratio of 90:10 (*R*,*R*)‐(*M*,*M*)‐*cis*‐**1 b**:(*R*,*R*)‐(*P*,*P*)‐*trans*‐**1 b** was obtained after irradiation of a solution of (*P*,*P*)‐*trans*‐**1 b** in [D_8_]THF (312 nm) for 2 h. Both isomers could be easily separated by preparative thin layer chromatography. After THI, the ^1^H NMR spectrum obtained for (*R*,*R*)‐(*P*,*P*)‐*cis*‐**1 b** was identical to that of the corresponding synthetic sample. Additionally, the effect of the presence of chloride anions was studied by CD spectroscopy (Figure 2 c). The addition of 10 equiv of TBACl to a THF solution of (*R*,*R*)‐(*P*,*P*)‐*cis*‐**1 b** (black line in Figure 2 c) and (*R*,*R*)‐(*M*,*M*)‐*cis*‐**1 b** (blue line in Figure 2 c) resulted in an enhanced CD signal with negative ((*R*,*R*)‐(*P*,*P*)‐*cis*‐**1 b**, red line in Figure 2 c) and positive ((*R*,*R*)‐(*M*,*M*)‐*cis*‐**1 b**, green line in Figure 2 c) signs between 300 and 400 nm, which is indicative of the formation of supramolecular helical structures with opposite configurations upon anion binding.^22^ Job plot analysis indicated a 1:1 binding stoichiometry for the binding of chloride ions to the three isomers of **1 b** (see Supporting Information for details).

**Figure 2:**
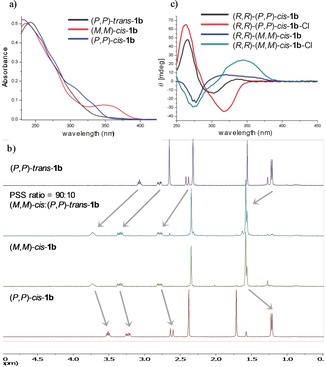
a) UV/Vis spectra (degassed THF, 10^−5^ m) and b) ^1^H NMR spectra (CD_2_Cl_2_) of (*P*,*P*)‐*trans*, (*M*,*M*)‐*cis*, and (*P*,*P*)‐*cis*‐**1 b**. c) CD spectra at 20 °C in degassed THF (10^−5^ m) of (*M*,*M*)‐*cis*, and (*P*,*P*)‐*cis*‐**1 b** before and after the addition of 10 equivalent TBACl.

We next evaluated the stereochemical outcome of the reaction catalyzed by each of the interconvertible isomers of **1 b** generating 1‐chloroisochroman derivatives **8** in situ from the corresponding 1‐methoxyisochromans **11** (Scheme 3). The reaction of **8 a** in the presence of (*R*,*R*)‐(*P*,*P*)‐*trans*‐**1 b** under the previously optimized reaction conditions led to the formation of nearly racemic **10 a** (e.r.=51:49), which was isolated in 48 % yield. The reaction catalyzed by (*R*,*R*)‐(*P*,*O*)‐*cis*‐**1 b** afforded as expected (*S*)‐**10 a** in 90 % yield and 10:90 e.r. Gratifyingly, the use of pure (*R*,*R*)‐(*M*,*M*)‐*cis*‐**1 b**, which results from the irradiation of (*R*,*R*)‐(*P*,*P*)‐*trans*‐**1 b** with 312 nm light, promoted the preferential formation of the opposite enantiomer, giving rise to (*R*)‐**10 a** in 47 % yield and 74:26 e.r. Notably, the conversion reached after 5 days was significantly lower in the presence of both (*R*,*R*)‐(*P*,*P*)‐*trans*‐**1 b** and (*R*,*R*)‐(*M*,*M*)‐*cis*‐**1 b** as compared to (*R*,*R*)‐(*P*,*P*)‐*cis*‐**1 b**, which was consistently observed for other 1‐chloroisochroman derivatives. In the case of (*R*,*R*)‐(*P*,*P*)‐*trans*‐**1 b**, the lower activity can be rationalized based on the absence of cooperativity between the two oligotriazole branches, whereas in the case of (*R*,*R*)‐(*M*,*M*)‐*cis*‐**1 b**, we attribute this effect to a less optimal geometry for chloride binding. Differences in catalytic activity between (*M*,*M*)‐*cis* and (*P*,*P*)‐*cis* motors had been previously observed in our group in the context of stereodivergent catalysis.^8^ In a similar vein, methyl‐substituted derivatives **11 b** and **11 c** gave rise to racemic **10 b** and **10 c** in the presence of (*R*,*R*)‐(*P*,*P*)‐*trans*‐**1 b**, whereas the use of (*R*,*R*)‐(*M*,*M*)‐*cis*‐**1 b** and (*R*,*R*)‐(*P*,*P*)‐*cis*‐**1 b** promoted the formation of enantioenriched products. In the case of **10 b**, opposite enantiomers were preferentially obtained in 65:35 e.r. and 14:86 e.r. through the use of (*R*,*R*)‐(*M*,*M*)‐*cis*‐**1 b** and (*R*,*R*)‐(*P*,*P*)‐*cis*‐**1 b**, respectively, which corresponds to 102 % Δ*ee*. Likewise, reversal of stereoselectivity in the formation of **10 c** was achieved, obtaining a remarkable 142 % Δee when switching from (*R*,*R*)‐(*M*,*M*)‐*cis*‐**1 b** to (*R*,*R*)‐(*P*,*P*)‐*cis*‐**1 b**. Unfortunately, (*R*,*R*)‐(*M*,*M*)‐*cis*‐**1 b** was not able to promote the addition of **9** to **8 d**–**f** under the optimized reaction conditions and only starting material was recovered after 5 days. This lack of reactivity is attributed to the low solubility observed for its adducts with the corresponding 1‐chloroisochroman derivatives at the optimized reaction temperature. Conducting the reaction at higher temperatures (over −20 °C) led to conversion to the product, although not surprisingly, the levels of enantioinduction were very poor. Nonetheless, in all those cases switching between (*R*,*R*)‐(*P*,*P*)‐*trans*‐**1 b** and (*R*,*R*)‐(*P*,*P*)‐*cis*‐**1 b** allowed for control on the stereochemical outcome in the formation of **10 d**–**f**, which were obtained as racemates in the presence of (*R*,*R*)‐(*P*,*P*)‐*trans*‐**1 b** and as enantioenriched products when (*R*,*R*)‐(*P*,*P*)‐*cis*‐**1 b** was used, in up to 95:5 e.r. in the case of **10 e**.

**Scheme 3:**
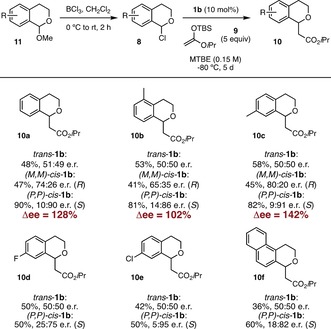
Stereodivergent addition of silyl ketene acetal **9** to 1‐chloroisochromans **8** catalyzed by motors **1 b**.

In summary, we have developed a photoresponsive chiral oligotriazole anion receptor based on a molecular motor core, which promotes the stereodivergent addition of silyl ketene acetal nucleophiles to oxocarbenium ions via anion binding catalysis. The stereoselectivity of this transformation can be modulated through the light‐ and heat‐driven rotation around the double bond axis of the molecular motor, which functions as a multistage chiral switch. Products **10** were obtained as racemates in the presence of the (*P*,*P*)‐*trans* state, and in up to 80:20 e.r. with (*M*,*M*)‐*cis* state and 5:95 e.r. with (*P*,*P*)‐*cis* state, promoting each of the *cis* states the formation of opposite enantiomers. The unidirectionality of molecular motors translates into a defined sequence of isomers formed as one moves forward in the cycle. In our particular case, the cycle (*R*,*R*)‐(*P*,*P*)‐*trans*‐**1 b**–(*R*,*R*)‐(*M*,*M*)‐*cis*‐**1 b**–(*R*,*R*)‐(*P*,*P*)‐*cis*‐**1 b** gives rise to addition products in the order (*R*,*S*)racemic–*R* enantiomer–*S* enantiomer. Starting from the (*S*,*S*)‐configured motor would invert the sequence resulting in (*R*,*S*)racemic–*S* enantiomer–*R* enantiomer. Although with lower absolute enantioselectivities than those reported for non‐switchable organocatalysts, reversal of enantioselectivity with up to 142 % Δ*ee* was achieved in this work starting from a single enantiomer of the catalyst through simple irradiation and heating steps. These results highlight the potential of helicates with switchable configuration for asymmetric synthesis and open up new avenues not only for future applications in the field of photoswitchable catalysis but also in the arena of responsive supramolecular assemblies.

## Conflict of interest

The authors declare no conflict of interest.

## Supporting information

As a service to our authors and readers, this journal provides supporting information supplied by the authors. Such materials are peer reviewed and may be re‐organized for online delivery, but are not copy‐edited or typeset. Technical support issues arising from supporting information (other than missing files) should be addressed to the authors.

Supplementary
