# Supplementary material for: Stereodivergent Anion Binding Catalysis with Molecular Motors
Source: Angew Chem Int Ed Engl. 2019 Dec 12;59(2):785–9. doi: 10.1002/anie.201913054 (PMC7004205; doi:10.1002/anie.201913054)
Supplement: Supplementary file 1 — Supplementary [file ANIE-59-785-s001.pdf]

Angewandte Chemie
Eine Zeitschrift der Gesellschaft Deutscher Chemiker

Supporting Information

Stereodivergent Anion Binding Catalysis with Molecular Motors

*Ruth Dorel and Ben L. Feringa**

anie_201913054_sm_miscellaneous_information.pdf

Supporting Information

Table of contents

1. **General methods**……………………………………………………………………S1
2. **Experimental procedures**…………………………………………………………..S2
   2.1. Synthesis of motors (*R*,*R*)-(*P,P*)-*cis*-**4** and (*R*,*R*)-(*P,P*)-*trans*-**4**……………………S2
   2.2. Synthesis of motors (*R*,*R*)-(*P,P*)-*cis*-**1a-d**……………………………………………S6
   2.3. Synthesis of motor (*R*,*R*)-(*P,P*)-*cis*-**7**……………………………………………..S17
   2.4. Synthesis of motor (*R*,*R*)-(*P,P*)-*trans*-**1b**…………………………………………S18
3. **Isomerization of motor 1b**………………………………………………………...S19
   3.1. UV-vis irradiation studies………………………………………………………...S19
   3.2. NMR irradiation studies…………………………………………………………..S20
   3.3. Kinetic studies on the THI step…………………………………………………S21
4. **Job Plot analysis of motor 1b with TBACl**…………...……………………………S22
5. **Catalysis experiments**……………………………………………………………..S24
   5.1. Optimization of the addition of nucleophiles to **8a**……………………...……S24
   5.2. General procedure for the addition of **9a** to **8**……………………………………S25
   5.3. Characterization of products **10**…………………………………………………S25
   5.4. Assignment of the absolute configuration of products **10**…..…………………...S27
6. **NMR spectra**……………………………………………………………………...S28
7. **HPLC and SFC traces**……………………………………………………………S67

1. General methods

All reagents were purchased from commercial sources and used as received without further purification. Dry solvents were obtained from a MBraun solvent purification system. Column chromatography was performed on silica gel (Merck type 9385 230-400 mesh) or on a Reveleris X2 Flash Chromatography system; TLC: silica gel 60, Merck, 0.25 mm. High Resolution Mass spectra (HRMS) were recorded on an LTQ Orbitrap XL. NMR spectra were obtained using a Varian Mercury Plus (^{1}H: 400 MHz, ^{13}C: 100 MHz), a Varian Unity Plus (^{1}H: 500 MHz, ^{13}C: 125 MHz) or a Bruker Innova (^{1}H: 600 MHz, ^{13}C: 151 MHz) in $CDCl_3$ or CD_2Cl_2. Chemical shifts are reported in δ units (ppm) relative to the residual deuterated solvent signal of $CDCl_3$ (^{1}H NMR, δ 7.26 ppm; ^{13}C NMR, δ 77.2 ppm) or CD_2Cl_2 (^{1}H NMR, δ 5.32 ppm; ^{13}C NMR, δ 53.5 ppm). The splitting patterns are designated as follows: s (singlet), d (doublet), t (triplet), q (quartet), dd (doublet of doublets), dt (doublet of triplets), ddd (doublet of doublets of doublets), dtd (doublet of triplets of doublets), ddt (doublet of doublets of triplets), td (triplet of doublets), and m (multiplet).

Chiral HPLC analysis was performed using a Shimadzu LC 10ADVP HPLC equipped with a Shimadzu SPDM10AVP diode array detector using a Chiralpak columns with mixtures of HPLC-grade heptane and 2-propanol as eluent and column temperature of 40°C. Sample injections were made using a HP 6890 Series Auto sample Injector. Supercritical fluid chromatography (SFC) was performed on a TharTechnologies, Inc. (Waters) Investigator II system.

UV-vis absorption spectra were measured on a SPECORD S600 Analityk Jena spectrophotometer, equipped with a QUANTUM Northwest TC-1 temperature controller and fluorescence temperature control cell. CD spectra were measured on a Jasco J-815 CD spectrometer. The solvents used for spectroscopic studies were of spectroscopic grade (UVASOL Merck) and were degassed prior to the spectroscopic measurements. Irradiations were performed using a spectroline ENB-280C/FE lamp (λ max = 312 nm).

2. Experimental procedures

2.1. Synthesis of motors (*P,P*)-*cis*-**4** and (*P,P*)-*trans*-**4**

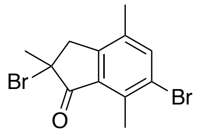

2,6-Dibromo-2,4,7-trimethyl-2,3-dihydro-1*H*-inden-1-one (S1). 2,4,7-Trimethyl-2,3-dihydro-1*H*-inden-1-one[1] (21.8 g, 124.6 mmol) was dissolved in MeCN (350 mL) and aqueous H_2SO_4 (10% v/v, 300 mL) was subsequently added at rt followed by the portionwise addition of *N*-bromosuccinimide (56.0 g, 314 mmol). The resulting mixture was stirred at 70 ºC for 8 h, then cooled to rt, poured into H_2O (500 mL), and extracted with EtOAc (3x500 mL). The combined organic layers were washed with a saturated aqueous $Na_2S_2O_3$ solution (2x600 mL) followed by a saturated aqueous $NaHCO_3$ solution (600 mL), water (600 mL), and brine (600 mL). The organic phase was dried over $MgSO_4$, filtered, and concentrated under reduced pressure to give the crude product as an orange oil, which was taken to the next step without further purification. Analytically pure product could also be obtained as a pale yellow solid after purification by column chromatography (pentane:Et_2O 80:20).

^{1}H NMR (400 MHz, $CDCl_3$) δ 7.53 (s, 1H), 3.37 (dd, *J* = 134.6, 18.4 Hz, 2H), 2.62 (s, 3H), 2.21 (s, 3H), 1.89 (s, 3H).

^{13}C NMR (151 MHz, $CDCl_3$) δ 200.3, 147.7, 139.1, 137.4, 134.2, 131.2, 125.7, 59.7, 44.2, 27.0, 17.2, 16.9.

HRMS (ESI+) *m/z* calc. for $C_{12}H_{13}Br_2O$ $[M+H]^+$: 330.9328. Found: 330.9151.

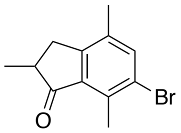

6-Bromo-2,4,7-trimethyl-2,3-dihydro-1*H*-inden-1-one (S2). NaI (146.3 g, 0.98 mol, 8 equiv) and TMSCl (54 mL, 0.43 mol, 3.5 equiv) were sequentially added to a solution of crude **S1** (40.5 g, *ca.* 122 mmol) in MeCN (1.2 L) and the resulting mixture was vigorously stirred at rt for 4 h. After the addition of H_2O (800 mL), the product was extracted with EtOAc (3x800 mL). The combined organic layers were washed with a saturated aqueous $Na_2S_2O_3$ solution until the color changed to intense yellow. The organic layer was then washed with brine (1 L), dried over $MgSO_4$, filtered, and concentrated under reduced pressure. The crude product was first precipitated from CH_2Cl_2/MeOH, and then purified by column chromatography (pentane:Et_2O 100:0 to 90:10). **S2** was obtained as a pale yellow solid (18.9-22.1 g, yield over 2 steps = 60-70%). The spectroscopic data were consistent with those previously reported.[2]

^{1}H NMR (400 MHz, $CDCl_3$) δ 7.43 (s, 1H), 3.13 (dd, *J* = 17.0, 7.9 Hz, 2H), 2.60 (s, 3H), 2.45 (d, *J* = 17.6 Hz, 1H), 2.27 (s, 3H), 1.31 (d, *J* = 7.3 Hz, 3H).

---

1 Prepared according to: Kaminsky, W.; Rabe, O.; Schauwienold, A.-M.; Schupfner, G. U.; Hanss, J.; Kopf, J. *J. Organometallic Chem.* **1995**, *497*, 181–193.

2 Wang, J.; Kulago, A.; Browne, W. R.; Feringa, B. L. *J. Am. Chem. Soc.* **2010**, *132*, 4191–4196.

^{13}C NMR (101 MHz, $CDCl_3$) δ 209.5, 152.4, 137.8, 135.7, 134.6, 134.3, 125.0, 42.3, 32.8, 17.3, 16.6 16.5.

Note: Enantioenriched ketone (*R*)-**S2** was obtained in up to 93% ee following the procedure reported in the literature.[3]

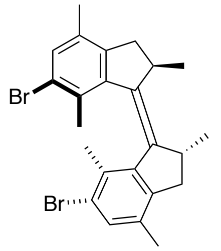

(2*R*,2'*R*,*Z*)-6,6'-Dibromo-2,2',4,4',7,7'-hexamethyl-2,2',3,3'-tetrahydro-1,1'-biindenylidene ((*R*,*R*)-(*P*,*P*)-*cis*-2). A mixture of (*R*,*R*)-(*P,P*)-*cis*-**2** and (*R*,*R*)-(*P*,*P*)-*trans*-**2** was prepared through the McMurry coupling of (*R*)-**S2** following the literature procedures.[2,4] In order to obtain pure (*R*,*R*)-(*P*,*P*)-*cis*-**2**, the mixture of isomers (1.0 g, 2.11 mmol) was placed in a pressure tube, suspended in heptane (5 mL) and heated at 200 ºC for 5 h. After cooling to rt the solvent was evaporated and and product purified by column chromatography (pentane). (*R*,*R*)-(*P*,*P*)-cis-**2** was obtained as a white solid (920 mg, 1.94 mmol, yield = 92%).

^{1}H NMR (400 MHz, $CDCl_3$) δ 7.25 (s, 2H), 3.34 (p, J = 6.7 Hz, 2H), 3.04 (dd, J = 15.2, 6.4 Hz, 2H), 2.41 (d, J = 15.1 Hz, 2H), 2.23 (s, 6H), 1.51 (s, 6H), 1.08 (d, J = 6.7 Hz, 6H).

^{13}C NMR (151 MHz, $CDCl_3$) δ 143.3, 142.2, 141.2, 132.6, 132.3, 131.9, 123.5, 41.7, 38.4, 21.4, 20.3, 18.1.

HRMS (ESI+) *m/z* calc. for $C_{24}H_{27}Br_2$ $[M+H]^+$: 473.0474. Found: 473.0301.

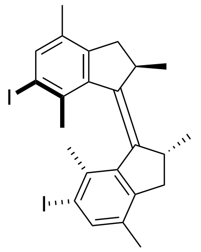

(2*R*,2'*R*,*Z*)-6,6'-Diiodo-2,2',4,4',7,7'-hexamethyl-2,2',3,3'-tetrahydro-1,1'-biindenylidene ((*R*,*R*)-(*P*,*P*)-*cis*-3). (*R*,*R*)-(*P*,*P*)-*cis*-**2** (800 mg, 1.68 mmol), NaI (1.51 g, 10.08 mmol), CuI (48 mg, 0.25 mmol), and *trans*-*N*,*N'*-dimethylcyclohexane-1,2-diamine (79 μL, 0.50 mmol) were mixed in anhydrous and degassed dioxane (8 mL) in a pressure tube. The mixture was stirred at 140 ºC for 24 h under an argon atmosphere and then cooled to rt followed by the addition of H_2O (10 mL). The product was extracted with CH_2Cl_2 (2x15 mL) and the combined organic layers dried over $MgSO_4$, filtered, and concentrated under reduced pressure. Purification by column chromatography (pentane) afforded the product as a white solid (745 mg, 1.31 mmol, yield = 78%).

^{1}H NMR (600 MHz, $CDCl_3$) δ 7.56 (s, 2H), 3.35 (p, J = 6.7 Hz, 2H), 3.07 (dd, J = 15.2, 6.4 Hz, 2H), 2.43 (d, J = 15.2 Hz, 2H), 2.24 (s, 6H), 1.55 (s, 6H), 1.10 (d, J = 6.8 Hz, 6H).

---

3 Cheon, C. H.; Kanno, O.; Toste, F. D. *J. Am. Chem. Soc.* **2011**, 133, 13248–13251.

4 For the preparation of the enantioenriched motor, see: Neubauer, T. M.; van Leeuwen, T.; Zhao, D.; Lubbe, A. S. Kistemaker, J. C. M.; Feringa, B. L. *Org. Lett.* **2014**, *16*, 4220–4223.

^{13}C NMR (151 MHz, $CDCl_3$) δ 144.3, 141.4, 138.6, 135.6, 133.1, 100.3, 41.4, 38.5, 27.0, 20.4, 17.9 (one carbon missing due to overlapping).

HRMS (ESI+) *m/z* calc. for $C_{24}H_{25}I$ $[M-HI]^+$: 440.0996. Found: 440.0987.

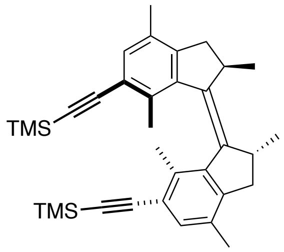

(((2*R*,2'*R*,*Z*)-2,2',4,4',7,7'-Hexamethyl-2,2',3,3'-tetrahydro-[1,1'-biindenylidene]-6,6'-diyl)bis(ethyne-2,1-diyl))bis(trimethylsilane) ((*R*,*R*)-(*P*,*P*)-*cis*-S3). To a solution of (*R*,*R*)-(*P,P*)-*cis*-**3** (568 mg, 1 mmol) in degassed triethylamine (10 mL) were sequentially added $PdCl_2(PPh_3)_2$ (70 mg, 0.1 mmol), CuI (38 mg, 0.2 mmol), and trimethylsilylacetylene (0.41 mL, 3 mmol) under a N_2 atmosphere. The reaction mixture was stirred at 70 ºC for 16 h, then cooled to rt, diluted with EtOAc (15 mL) and filtered through a short pad of silica. The volatiles were removed under reduced pressure and the crude mixture purified by column chromatography (pentane:EtOAc 100:0 to 95:5) to give the product as a white solid (412 mg, 0.81 mmol, yield = 81%).

^{1}H NMR (400 MHz, $CDCl_3$) δ 7.19 (s, 2H), 3.34 (p, *J* = 6.7 Hz, 2H), 3.08 (dd, *J* = 15.4, 6.5 Hz, 2H), 2.43 (d, *J* = 15.2 Hz, 2H), 2.22 (s, 6H), 1.56 (s, 6H), 1.04 (d, *J* = 6.8 Hz, 6H), 0.21 (s, 18H).

^{13}C NMR (101 MHz, $CDCl_3$) δ 147.8, 143. 5, 143.5, 138.3, 135.0, 133.3, 123.7, 107.8, 99.5, 44.3, 41.5, 23.0, 21.7, 20.8, 2.8.

HRMS (ESI+) *m/z* calc. for $C_{34}H_{45}Si_2$ $[M+H]^+$: 509.3054. Found: 509.3042.

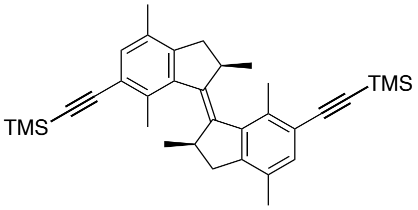

(((2*R*,2'*R*,*E*)-2,2',4,4',7,7'-Hexamethyl-2,2',3,3'-tetrahydro-[1,1'-biindenylidene]-6,6'-diyl)bis(ethyne-2,1-diyl))bis(trimethylsilane) ((*R*,*R*)-(*P*,*P*)-*trans*-S3). The procedure described for the preparation of (*R*,*R*)-(*P*,*P*)-*cis*-**S3** starting from a mixture of (*R*,*R*)-(*P*,*P*)-*cis*-**3** and (*R*,*R*)-(*P,P*)-*trans*-**3** afforded (*R*,*R*)-(*P*,*P*)-*cis*-**S3** and (*R*,*R*)-(*P,P*)-*trans*-**S3**, which could be separated by column chromatography (pentane:EtOAc 100:0 to 95:5). The title product was isolated as a white solid (yield = 75% from (*R*,*R*)-(*P*,*P*)-*trans*-**3**).

^{1}H NMR (400 MHz, $CDCl_3$) δ 7.19 (s, 2H), 2.84 (p, *J* = 6.4 Hz, 2H), 2.61 (dd, *J* = 15.0, 5.7 Hz, 2H), 2.50 (s, 6H), 2.20 (d, *J* = 15.0 Hz, 2H), 2.15 (s, 6H), 1.06 (d, *J* = 6.4 Hz, 6H), 0.28 (d, *J* = 1.1 Hz, 18H).

^{13}C NMR (101 MHz, $CDCl_3$) δ 146.1, 144.2, 143.8, 136.3, 134.8, 133.9, 124.1, 107.8, 99.7, 44.5, 41.8, 23.3, 21.6, 20.6, 2.8.

HRMS (ESI+) *m/z* calc. for $C_{34}H_{44}Si_2$ $[M]^+$: 508.2976. Found: 508.2972.

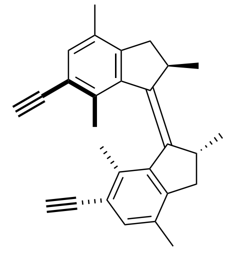

(2*R*,2'*R*,*Z*)-6,6'-Diethynyl-2,2',4,4',7,7'-hexamethyl-2,2',3,3'-tetrahydro-1,1'-biindenylidene ((*R*,*R*)-(*P*,*P*)-*cis*-4). TBAF (1 M solution in THF, 1.5 mL, 1.5 mmol) was added to a solution of (*R*,*R*)-(*P*,*P*)-*cis*-**S3** (254 mg, 0.5 mmol) in THF (5 mL) at 0 ºC and the reaction was stirred for 1 h at rt. After the addition of Et_2O (10 mL), the mixture was washed with a saturated aqueous solution of NH_4Cl (10 mL) and brine. The aqueous layers were extracted with Et_2O (10 mL) and the combined organic phases were dried over $MgSO_4$, filtered, and concentrated under reduced pressure. Purification by column chromatography (pentane:EtOAc 99:1) afforded the product as a white solid (412 mg, 0.81 mmol, yield = 99%).

^{1}H NMR (400 MHz, $CDCl_3$) δ 7.20 (s, 2H), 3.36 (p, *J* = 6.7 Hz, 2H), 3.17 (s, 2H), 3.10 (dd, *J* = 15.3, 6.4 Hz, 2H), 2.45 (d, *J* = 15.3 Hz, 2H), 2.24 (s, 6H), 1.58 (s, 6H), 1.06 (d, *J* = 6.8 Hz, 6H).

^{13}C NMR (101 MHz, $CDCl_3$) δ 148.1, 143.6, 143.5, 138.3, 135.4, 133.4, 122.8, 86.2, 82.6, 44.3, 41.5, 23.0, 21.6, 20.8.

HRMS (ESI+) *m/z* calc. for $C_{28}H_{29}$ $[M+H]^+$: 365.2264. Found: 365.2199.

Recrystallization from hot hexane afforded the enantiopure product (*R*,*R*)-(*P*,*P*)-*cis*-**4** (e.r. > 99:1). HPLC analysis: OD-H, 40 ºC, 0.3 mL/min, heptane 100%, t_1 = 28.8 min, t_2 = 31.5 min.

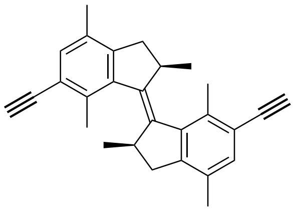

(2*R*,2'*R*,*E*)-6,6'-Diethynyl-2,2',4,4',7,7'-hexamethyl-2,2',3,3'-tetrahydro-1,1'-biindenylidene ((*R*,*R*)-(*P*,*P*)-*trans*-4). Procedure described for (*R*,*R*)-(*P*,*P*)-*cis*-**4** starting from (*R*,*R*)-(*P*,*P*)-*trans*-**S3**. White solid (yield = 72%).

^{1}H NMR (400 MHz, $CDCl_3$) δ 7.22 (s, 2H), 3.26 (s, 2H), 2.84 (p, *J* = 6.4 Hz, 2H), 2.63 (dd, *J* = 14.9, 5.7 Hz, 2H), 2.53 (s, 6H), 2.23 (d, *J* = 14.9 Hz, 2H), 2.17 (s, 6H), 1.08 (d, *J* = 6.3 Hz, 6H).

^{13}C NMR (151 MHz, $CDCl_3$) δ 143.6, 141.6, 141.2, 133.8, 132.5, 131.4, 120.5, 83.5, 80.0, 41.9, 39.2, 20.7, 18.9, 18.0.

HRMS (ESI+) *m/z* calc. for $C_{28}H_{28}$ $[M]^+$: 364.2186. Found: 364.2186.

2.2. <u>*Synthesis of motors (P,P)-cis-**1a-d***</u>

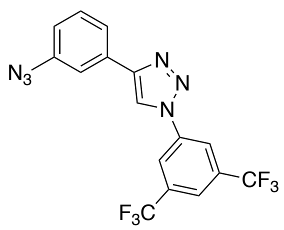

4-(3-Azidophenyl)-1-(3,5-bis(trifluoromethyl)phenyl)-1*H*-1,2,3-triazole (5a). $NaNO_2$ (30 mg, 0.44 mmol) was added to a solution of 3-(1-(3,5-bis(trifluoromethyl)phenyl)-1*H*-1,2,3-triazol-4-yl)aniline[5] (150 mg, 0.4 mmol) in TFA (4 mL) at 0 ºC. After stirring for 1 h at that temperature, NaN_3 (39 mg, 0.6 mmol) was added and the resulting mixture was allowed to warm to rt and stirred for 1.5 h before the addition of H_2O (10 mL). The product was extracted with CH_2Cl_2 (3x10 mL) and the combined organic layers were dried over $MgSO_4$, filtered, and concentrated under reduced pressure. Purification by column chromatography (pentane:EtOAc 98:2) afforded the title compound as an off-white solid (137 mg, 0.34 mmol, yield = 86%).

^{1}H NMR (400 MHz, $CDCl_3$) δ 8.34 (s, 1H), 8.31 (s, 2H), 7.98 (s, 1H), 7.69 (d, *J* = 7.8 Hz, 1H), 7.61 (d, *J* = 2.1 Hz, 1H), 7.47 (t, *J* = 7.9 Hz, 1H), 7.11 – 7.03 (m, 1H).

^{13}C NMR (151 MHz, $CDCl_3$) δ 148.5, 141.1, 137.9, 133.8 (q, *J* = 34.4 Hz), 131.2, 130.5, 122.6 (q, *J* = 273.3 Hz), 122.5, 122.30 (hept, *J* = 3.8 Hz), 120.3 (q, *J* = 3.8 Hz), 119.5, 117.6, 116.5.

^{19}F NMR (376 MHz, $CDCl_3$) δ -63.02.

HRMS (ESI+) *m/z* calc. for $C_{16}H_9F_6N_6$ $[M+H]^+$: 399.0787. Found: 399.0784.

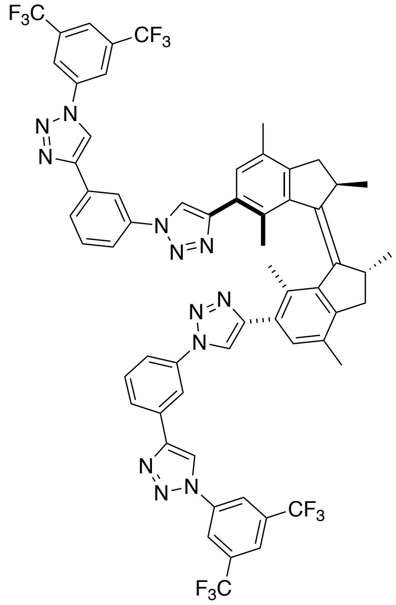

4,4'-((2*R*,2'*R*,*Z*)-2,2',4,4',7,7'-Hexamethyl-2,2',3,3'-tetrahydro-[1,1'-biindenylidene]-6,6'-diyl)bis(1-(3-(1-(3,5-bis(trifluoromethyl)phenyl)-1*H*-1,2,3-triazol-4-yl)phenyl)-1*H*-1,2,3-triazole) ((*R*,*R*)-(*P*,*P*)-*cis*-1a). In a pressure tube, (*R*,*R*)-(*P*,*P*)-*cis*-**4** (36 mg, 0.10 mmol) and **5a** (100 mg, 0.25 mmol) were disolved in a 1:1 mixture of CH_2Cl_2:*t*BuOH (2 mL). A solution of $CuSO_4 \cdot 5H_2O$ (25 mg, 0.10 mmol) in H_2O (1 mL) was next added followed by a solution of sodium

5 Prepared according to: Altimari, J. M.; Hockey, S. C.; Boshoff, H. I.; Sajid, A.; Henderson, L. C. *ChemMedChem* **2015**, *10*, 787–791.

ascorbate (50 mg, 0.25 mmol) in H_2O (1 mL), and the resulting mixture was stirred at 70 ºC for 16 h. After cooling to rt, the reaction was diluted with CH_2Cl_2 (10 mL) and a 1 M aqueous solution of KCN (10 mL) was added. The mixture was stirred at rt for 30 min and then the layers were separated. The organic phase was further washed with a 1 M aqueous solution of KCN (2x10 mL), then dried over $MgSO_4$, filtered, and concentrated under reduced pressure. Purification by column chromatography (pentane:CH_2Cl_2:EtOAc 70:30:0 to 0:100:0, then 0:98:2) afforded the product as a pale yellow solid (74 mg, 0.064 mmol, yield = 64%).

^{1}H NMR (600 MHz, CD_2Cl_2) δ 8.54 (s, 2H), 8.39 (s, 2H), 8.20 (s, 4H), 8.13 (t, *J* = 1.9 Hz, 2H), 7.97 (dt, *J* = 7.8, 1.3 Hz, 2H), 7.94 – 7.90 (m, 1H), 7.82 (ddd, *J* = 8.1, 2.2, 1.0 Hz, 2H), 7.53 (t, *J* = 7.9 Hz, 2H), 7.36 (s, 2H), 3.45 (p, *J* = 6.8 Hz, 2H), 3.17 (dd, *J* = 15.4, 6.5 Hz, 2H), 2.55 (d, *J* = 15.4 Hz, 2H), 2.32 (s, 6H), 1.66 (s, 6H), 1.16 (d, *J* = 6.8 Hz, 6H).

^{13}C NMR (151 MHz, CD_2Cl_2) δ 149.0, 147.7, 145.0, 142.1, 141.3, 137.8, 137.5, 133.2 (q, *J* = 34.3 Hz), 131.4, 131.4, 131.1, 130.6, 129.3, 128.5, 125.4, 122.7 (q, *J* = 273.0 Hz), 122.2 (hept, *J* = 3.7 Hz) , 120.2 (q, *J* = 3.7 Hz), 120.0, 119.5, 118.7, 41.2, 38.6, 20.3, 20.0, 18.0.

^{19}F NMR (565 MHz, CD_2Cl_2) δ -63.25.

HRMS (ESI+) *m/z* calc. for $C_{60}H_{45}F_{12}N_{12}$ $[M+H]^+$: 1161.3693. Found: 1161.3682.

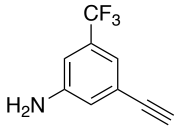

3-Ethynyl-5-(trifluoromethyl)aniline (S4). TBAF (1 M solution in THF, 11.7 mL, 11.66 mmol) was added to a solution of 3-(trifluoromethyl)-5-((trimethylsilyl)ethynyl)aniline[6] (2.00 g, 7.77 mmol) in THF (78 mL) at 0 ºC and the resulting mixture was allowed to warm to rt and stirred for 1 h. The reaction mixture was diluted with Et_2O (40 mL) and washed with brine (50 mL). The aqueous layer was extracted with Et_2O (40 mL) and the combined organic layers were dried over $MgSO_4$, filtered, and concentrated under reduced pressure. Purification by flash column chromatography (pentane:CH_2Cl_2 9:1 to 1:1) afforded the product as a clear oil (1.12 g, 6.06 mmol, yield = 78%).

^{1}H NMR (600 MHz, $CDCl_3$) δ 7.12 (s, 1H), 6.92 (t, *J* = 1.7 Hz, 1H), 6.86 (t, *J* = 2.0 Hz, 1H), 3.89 (s, 2H), 3.07 (s, 1H).

^{13}C NMR (151 MHz, $CDCl_3$) δ 146.7, 132.0 (q, *J* = 32.3 Hz), 123.7, 123.6 (q, *J* = 272.7 Hz), 121.0, 118.8 (q, *J* = 3.9 Hz), 111.8 (q, *J* = 3.8 Hz), 82.5, 77.9.

^{19}F NMR (565 MHz, $CDCl_3$) δ -63.27.

HRMS (ESI+) *m/z* calc. for $C_9H_7F_3N$ $[M+H]^+$: 186.0525. Found: 186.0521.

6 Prepared according to: Zurro, M.; Asmus, S.; Beckendorf, S.; Mück-Lichtenfeld, C.; García-Mancheño, O. *J. Am. Chem. Soc.* **2014**, *136*, 13999–14002.

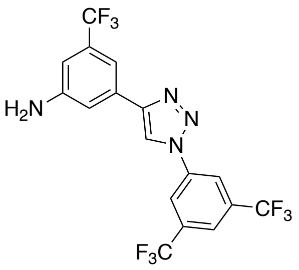

3-(1-(3,5-Bis(trifluoromethyl)phenyl)-1*H*-1,2,3-triazol-4-yl)-5-(trifluoromethyl)aniline (S5). **S4** (226 mg, 1.22 mmol) and 1-azido-3,5-bis(trifluoromethyl)benzene[7] (405 mg, 1.59 mmol) were disolved in a 1:1 mixture of CH_2Cl_2:*t*BuOH (12 mL). A solution of $CuSO_4·5H_2O$ (15 mg, 0.06 mmol) in H_2O (6 mL) was subsequently added followed by a solution of sodium ascorbate (36 mg, 0.18 mmol) in H_2O (6 mL), and the reaction was stirred at 50 ºC for 6 h. After cooling to rt CH_2Cl_2 (20 mL), an aqueous solution of KCN (10 wt%, 20 mL) and a saturated solution of Na_2CO_3 (5 mL) were added. The resulting mixture was stirred at rt for 10 min. The aqueous layer was extracted with CH_2Cl_2 (2x20 mL) and the combined organic layers were dried over $MgSO_4$, filtered, and concentrated under reduced pressure. Purification by flash column chromatography (pentane:EtOAc 98:2 to 8:2) afforded the product as a yellow solid (381 mg, 0.87 mmol, yield = 71%).

^{1}H NMR (600 MHz, $CDCl_3$) δ 8.35 (s, 1H), 8.31 (s, 2H), 7.98 (s, 1H), 7.52 (s, 1H), 7.42 (s, 1H), 6.93 (s, 1H), 4.05 (s, 2H).

^{13}C NMR (151 MHz, $CDCl_3$) δ 148.3, 147.4, 137.9, δ 133.74 (q, *J* = 34.5 Hz), 132.55 (q, *J* = 32.5 Hz), 131.11, 123.9 (q, *J* = 271.3 Hz), 122.5 (q, *J* = 273.2 Hz), 122.3 (hept, *J* = 7.1, 3.0 Hz), 120.3 (q, *J* = 4.3 Hz), 117.7, 115.0, 112.6 (q, *J* = 3.1 Hz), 111.7 (q, *J* = 2.8 Hz).

^{19}F NMR (565 MHz, $CDCl_3$) δ -63.02 (s, 6H), -63.08 (s, 3H).

HRMS (ESI+) *m/z* calc. for $C_{17}H_{10}F_9N_4$ $[M+H]^+$: 441.0756. Found: 441.0757.

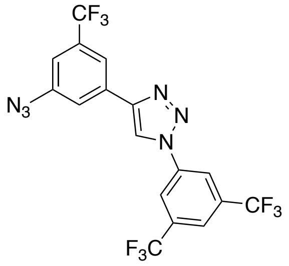

4-(3-Azido-5-(trifluoromethyl)phenyl)-1-(3,5-bis(trifluoromethyl)phenyl)-1*H*-1,2,3-triazole (5b). To a stirred solution of **S5** (150 mg, 0.34 mmol) in TFA (3.4 mL) at 0 ºC was added $NaNO_2$ (28 mg, 0.41 mmol) in one portion and the reaction mixture was vigorously stirred at that temperature for 1 h. NaN_3 (33 mg, 0.51 mmol) was subsequently added and the reaction mixture was allowed to warm to rt and stirred for an additional hour before diluting with H_2O (10 mL). The product was extracted with CH_2Cl_2 (15 mL) and the organic layer was washed with a saturated solution of $NaHCO_3$ (10 mL), brine (10 mL) and H_2O (10 mL), then dried over $MgSO_4$, filtered, and concentrated under reduced pressure. Purification by flash column chromatography (pentane:EtOAc 95:5) afforded the product as a clear oil, which turned into a white solid upon standing (135 mg, 0.29 mmol, yield = 85%).

7 Prepared according to: Beckendorf, S.; Asmus, S.; Mück-Lichtenfeld, C.; García-Mancheño, O. *Chem. Eur. J.* **2013**, *19*, 1581–1585.

^{1}H NMR (600 MHz, $CDCl_3$) δ 8.47 (s, 1H), 8.35 (d, *J* = 1.5 Hz, 2H), 8.03 (s, 1H), 7.92 (s, 1H), 7.85 (t, *J* = 1.7 Hz, 1H), 7.30 (d, *J* = 1.9 Hz, 1H).

^{13}C NMR (151 MHz, $CDCl_3$) δ 147.2, 142.2, 137.7, 133.8 (q, *J* = 34.5 Hz), 133.3 (q, *J* = 33.2 Hz), 132.1, 123.3 (q, *J* = 272.7 Hz), 122.6 (hept, *J* = 3.7 Hz), 122.5 (q, *J* = 273.2 Hz), 120.4 (q, *J* = 3.8 Hz), 119.3, 119.0 (q, *J* = 3.8 Hz), 118.2, 116.2 (q, *J* = 3.8 Hz).

^{19}F NMR (565 MHz, $CDCl_3$) δ -63.02 (s, 6F), -63.05 (s, 3F).

HRMS (ESI+) *m/z* calc. for $C_{17}H_8F_9N_6$ $[M+H]^+$: 467.0661. Found: 467.0657.

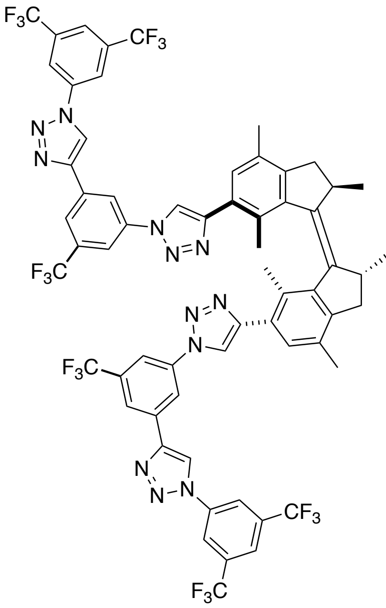

4,4'-((2*R*,2'*R*,*Z*)-2,2',4,4',7,7'-Hexamethyl-2,2',3,3'-tetrahydro-[1,1'-biindenylidene]-6,6'-diyl)bis(1-(3-(1-(3,5-bis(trifluoromethyl)phenyl)-1*H*-1,2,3-triazol-4-yl)-5-(trifluoromethyl)phenyl)-1*H*-1,2,3-triazole) ((*R*,*R*)-(*P*,*P*)-*cis*-1b). (*R*,*R*)-(*P*,*P*)-*cis*-**4** (30 mg, 0.08 mmol) and **5b** (93 mg, 0.2 mmol) were dissolved in a 1:1 mixutre of CH_2Cl_2:*t*BuOH (2 mL). A solution of $CuSO_4·5H_2O$ (40 mg, 0.16 mmol) in H_2O (1 mL) was subsequently added followed by a solution of sodium ascorbate (64 mg, 0.32 mmol) in H_2O (1 mL), and the reaction mixture was stirred at 70 ºC for 16 h. After cooling to rt CH_2Cl_2 (5 mL), an aqueous solution of KCN (10%wt, 5 mL) and a saturated solution of Na_2CO_3 (2 mL) were added. The resulting mixture was stirred at rt for 10 min and then the phases were separated and the aqueous layer was extracted with CH_2Cl_2 (2x10 mL). The combined organic layers were washed with an aqueous solution of KCN (10 wt%, 10 mL), and then dried over $MgSO_4$, filtered, and concentrated under reduced pressure. Purification by flash column chromatography (pentane:CH_2Cl_2:EtOAc 7:3:0 to 0:1:0 to 0:98:2) afforded the product as a white solid (71 mg, 0.054 mmol, yield = 68%).

^{1}H NMR (600 MHz, CD_2Cl_2) δ 8.58 (s, 2H), 8.50 (s, 2H), 8.38 (t, *J* = 1.8 Hz, 2H), 8.21 (d, *J* = 1.6 Hz, 4H), 8.18 (t, *J* = 1.5 Hz, 2H), 8.09 (d, *J* = 1.9 Hz, 2H), 7.93 (s, 2H), 7.35 (s, 2H), 3.44 (p, *J* = 6.8 Hz, 2H), 3.16 (dd, *J* = 15.4, 6.5 Hz, 2H), 2.55 (d, *J* = 15.4 Hz, 2H), 2.31 (s, 6H), 1.64 (s, 6H), 1.15 (d, *J* = 6.8 Hz, 6H).

^{13}C NMR (151 MHz, CD_2Cl_2) δ 149.4, 146.5, 145.2, 142.1, 141.3, 138.0, 137.7, 133.4 (q, *J* = 34.4 Hz), 133.0 (q, *J* = 33.9 Hz), 132.5, 131.4, 131.2, 129.3, 128.1, 123.3 (q, *J* = 272.9 Hz), 122.7 (q, *J* = 273.1 Hz), 122.5 (hept, *J* = 3.8 Hz), 122.0 (q, *J* = 3.8 Hz), 120.3 (q, *J* = 3.9 Hz), 120.1, 119.5, 119.2, 116.4 (q, *J* = 3.8 Hz), 41.2, 38.6, 20.3, 20.0, 18.0.

^{19}F NMR (565 MHz, CD_2Cl_2) δ -63.31 (s, 6F), -63.37 (s, 12F).

HRMS (ESI+) *m/z* calc. for $C_{62}H_{41}F_{18}N_{12}$ $[M]^+$: 1295.3284. Found: 1295.3263.

SFC analysis: Chiralpak IC (70% CO_2:30% MeOH), 160 bar, 40 °C, 4 mL/min, t_1 = 9.5 min, t_2 = 14.1 min.

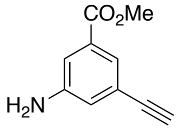

Methyl 3-amino-5-ethynylbenzoate (S6). TBAF (1 M solution in THF, 0.9 mL, 0.89 mmol) was added to a solution of methyl 3-amino-5-((trimethylsilyl)ethynyl)benzoate[8] (200 mg, 0.81 mmol) in THF (8 mL) at 0 ºC and the resulting mixture was allowed to warm to rt and stirred for 1 h. The reaction mixture was diluted with Et_2O (10 mL) and washed with brine (20 mL). The aqueous layer was extracted with Et_2O (20 mL) and the combined organic layers were dried over $MgSO_4$, filtered, and concentrated under reduced pressure. Purification by flash column chromatography (pentane:EtOAc 95:5 to 1:1) afforded the product as an off-white solid (115 mg, 0.66 mmol, yield = 81%).

^{1}H NMR (400 MHz, $CDCl_3$) δ 7.56 (t, *J* = 1.4 Hz, 1H), 7.33 (dd, *J* = 2.4, 1.5 Hz, 1H), 6.96 (dd, *J* = 2.5, 1.4 Hz, 1H), 3.89 (s, 3H), 3.88 (s, 2H), 3.04 (s, 1H).

^{13}C NMR (101 MHz, $CDCl_3$) δ 169.1, 148.9, 134.1, 126.2, 125.8, 124.8, 119.1, 85.5, 54.9.

HRMS (ESI+) *m/z* calc. for $C_{10}H_{10}N_1O_2$ $[M+H]^+$: 176.0706. Found: 176.0702.

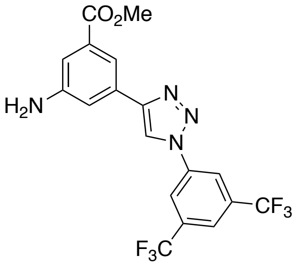

Methyl 3-amino-5-(1-(3,5-bis(trifluoromethyl)phenyl)-1*H*-1,2,3-triazol-4-yl)benzoate (S7). **S6** (214 mg, 1.22 mmol) and 1-azido-3,5-bis(trifluoromethyl)benzene[7] (405 mg, 1.59 mmol) were disolved in a 1:1 mixture of CH_2Cl_2:*t*BuOH (12 mL). A solution of $CuSO_4·5H_2O$ (15 mg, 0.06 mmol) in H_2O (6 mL) was subsequently added followed by a solution of sodium ascorbate (36 mg, 0.18 mmol) in H_2O (6 mL), and the reaction mixture was stirred at 50 ºC for 6 h. After cooling to rt CH_2Cl_2 (20 mL), an aqueous solution of KCN (10 wt%, 20 mL) and a saturated solution of Na_2CO_3 (5 mL) were added. The resulting mixture was stirred at rt for 10 min and then the phases were separated and the aqueous layer was extracted with CH_2Cl_2 (2x20 mL). The combined organic layers were dried over $MgSO_4$, filtered, and concentrated under reduced pressure. Purification by flash column chromatography (pentane:EtOAc 95:5 to 1:1) afforded the product as a yellow solid (394 mg, 0.92 mmol, yield = 75%).

^{1}H NMR (400 MHz, $CDCl_3$) δ 8.37 (s, 1H), 8.31 (s, 1H), 7.97 (s, 1H), 7.81 (t, *J* = 1.4 Hz, 1H), 7.58 (dd, *J* = 2.6, 1.3 Hz, 1H), 7.37 (dd, *J* = 2.4, 1.4 Hz, 1H), 3.93 (s, 3H), 3.92 (s, 2H).

8 Prepared according to: Pošta, M.; Soós, V.; Beier, P. *Tetrahedron* **2016**, *72*, 3809–3817.

^{13}C NMR (101 MHz, $CDCl_3$) δ 169.4, 151.3, 149.9, 140.6, 136.3 (q, *J* = 34.3 Hz), 134.6, 133.2, 125.2 (q, *J* = 273.4 Hz), 124.8 (hept, *J* = 7.9, 3.8 Hz), 122.9 (q, *J* = 3.2 Hz), 120.3, 119.7, 118.9, 118.8, 54.9.

^{19}F NMR (376 MHz, $CDCl_3$) δ -63.03.

HRMS (ESI+) *m/z* calc. for $C_{18}H_{13}F_6N_4O_2$ $[M+H]^+$: 431.0937. Found: 431.0940.

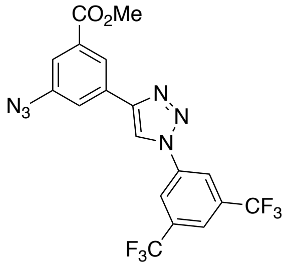

Methyl 3-azido-5-(1-(3,5-bis(trifluoromethyl)phenyl)-1*H*-1,2,3-triazol-4-yl)benzoate (5c). To a stirred solution of **S7** (146 mg, 0.34 mmol) in TFA (3.4 mL) at 0 ºC was added $NaNO_2$ (28 mg, 0.41 mmol) in one portion and the reaction mixture was vigorously stirred at that temperature for 1 h. NaN_3 (33 mg, 0.51 mmol) was subsequently added and the reaction mixture was allowed to warm to rt and stirred for an additional hour before diluting with H_2O (10 mL). The product was extracted with CH_2Cl_2 (15 mL) and the organic layer was washed with a saturated solution of $NaHCO_3$ (10 mL), brine (10 mL) and H_2O (10 mL), then dried over $MgSO_4$, filtered, and concentrated under reduced pressure. Purification by flash column chromatography (pentane:EtOAc 95:5) afforded the product as a white solid (126 mg, 0.28 mmol, yield = 81%).

^{1}H NMR (400 MHz, $CDCl_3$) δ 8.43 (s, 1H), 8.32 (s, 2H), 8.26 (t, *J* = 1.5 Hz, 1H), 8.00 (s, 2H), 7.88 (dd, *J* = 2.3, 1.5 Hz, 1H), 7.73 (dd, *J* = 2.3, 1.4 Hz, 1H), 3.98 (s, 3H).

^{13}C NMR (151 MHz, $CDCl_3$) δ 165.7, 147.6, 141.8, 137.8, 133.8 (q, *J* = 34.4 Hz), 132.7, 131.5, 123.3, 122.5 (q, *J* = 273.1 Hz), 122.5 (hept, *J* = 3.6 Hz), 120.5, 120.4 (q, *J* = 3.9 Hz), 120.1, 118.1, 52.6.

^{19}F NMR (376 MHz, $CDCl_3$) δ -63.03.

HRMS (ESI+) *m/z* calc. for $C_{18}H_{11}F_6N_6O_2$ $[M+H]^+$: 457.0842. Found: 457.0835.

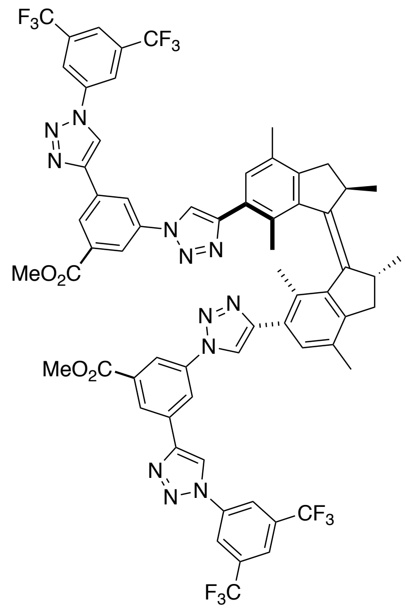

Dimethyl 5,5'-(((2*R*,2'*R*,*Z*)-2,2',4,4',7,7'-hexamethyl-2,2',3,3'-tetrahydro-[1,1'-biindenylidene]-6,6'-diyl)bis(1*H*-1,2,3-triazole-4,1-diyl))bis(3-(1-(3,5-bis(trifluoromethyl)phenyl)-1*H*-1,2,3-triazol-4-yl)benzoate) ((*R*,*R*)-(*P*,*P*)-*cis*-1c). (*R*,*R*)-(*P,P*)-*cis*-**4** (30 mg, 0.08 mmol) and **5c** (91 mg, 0.2 mmol) were dissolved in a 1:1 mixutre of CH_2Cl_2:*t*BuOH (2 mL). A solution of $CuSO_4{\cdot}5H_2O$ (40 mg, 0.16 mmol) in H_2O (1 mL) was subsequently added followed by a solution of sodium ascorbate (64 mg, 0.32 mmol) in H_2O (1 mL), and the reaction mixture was stirred at 70 ºC for 16 h. After cooling to rt, CH_2Cl_2 (5 mL), an aqueous solution of KCN (10 wt%, 5 mL) and a saturated solution of Na_2CO_3 (2 mL) were added. The resulting mixture was stirred at rt for 10 min and then the phases were separated and the aqueous layer was extracted with CH_2Cl_2 (2x10 mL). The combined organic layers were washed with an aqueous solution of KCN (10%wt, 10 mL), and then dried over $MgSO_4$, filtered, and concentrated under reduced pressure. Purification by flash column chromatography (pentane:CH_2Cl_2:EtOAc 7:3:0 to 0:1:0 to 0:95:5) afforded the product as a white solid (72 mg, 0.057 mmol, yield = 71%).

^{1}H NMR (400 MHz, CD_2Cl_2) δ 8.65 (s, 2H), 8.55 (s, 2H), 8.54 (t, *J* = 1.6 Hz, 2H), 8.45 – 8.41 (m, 4H), 8.26 (d, *J* = 1.7 Hz, 4H), 7.97 (s, 2H), 7.45 (s, 2H), 3.92 (s, 6H), 3.50 (p, *J* = 6.7 Hz, 2H), 3.23 (dd, *J* = 15.5, 6.5 Hz, 2H), 2.61 (d, *J* = 15.4 Hz, 2H), 2.38 (s, 6H), 1.72 (s, 6H), 1.21 (d, *J* = 6.8 Hz, 6H).

^{13}C NMR (101 MHz, CD_2Cl_2) δ 167.9, 151.8, 149.5, 147.7, 144.8, 144.0, 140.4, 135.9 (q, *J* = 34.4 Hz), 135.4, 134.4, 133.9, 133.8, 131.9, 130.8, 128.6, 125.3 (q, *J* = 273.0 Hz), 124.9 (q, *J* = 3.8 Hz), 122.9, 122.8, 122.7, 122.7, 121.6, 55.1, 43.9, 41.3, 22.9, 22.6, 20.6.

^{19}F NMR (376 MHz, CD_2Cl_2) δ -63.37.

HRMS (ESI+) *m/z* calc. for $C_{64}H_{49}F_{12}N_{12}O_4$ $[M+H]^+$: 1277.3803. Found: 1277.3814.

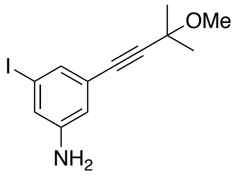

3-Iodo-5-(3-methoxy-3-methylbut-1-yn-1-yl)aniline (S8). To a solution of 3,5-diiodoaniline (1.50 g, 4.36 mmol) in degassed triethylamine (15 mL) were sequentially added $PdCl_2(PPh_3)_2$ (154 mg, 0.22 mmol), CuI (84 mg, 0.44 mmol), and a solution of 3-methoxy-3-methylbut-1-yne[9] (447 mg, 4.55 mmol) in degassed triethylamine (5 mL) under a N_2 atmosphere. The reaction mixture was stirred at rt for 16 h, then diluted with EtOAc (15 mL) and filtered through a short pad of silica. The volatiles were removed under reduced pressure and the crude mixture purified by column chromatography (pentane:EtOAc 9:1 to 1:1) to give the product as a yellow oil (577 mg, 1.83 mmol, yield = 42%).

^{1}H NMR (400 MHz, $CDCl_3$) δ 7.19 (t, *J* = 1.4 Hz, 1H), 7.02 (dd, *J* = 2.2, 1.5 Hz, 1H), 6.72 (dd, *J* = 2.2, 1.3 Hz, 1H), 4.04 (s, 2H), 3.40 (s, 3H), 1.51 (s, 6H).

^{13}C NMR (101 MHz, $CDCl_3$) δ 149.2, 133.5, 127.9, 126.7, 120.2, 96.8, 94.4, 85.4, 73.5, 54.4, 30.9.

HRMS (APCI+) *m/z* calc. for $C_{12}H_{15}INO$ $[M+H]^+$: 316.0193. Found: 316.0193.

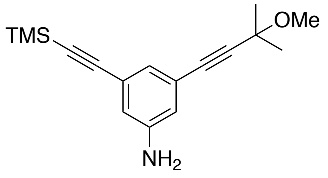

3-(3-Methoxy-3-methylbut-1-yn-1-yl)-5-((trimethylsilyl)ethynyl)aniline (S9). To a solution of **S8** (540 mg, 1.71 mmol) in degassed triethylamine (10 mL) were sequentially added $PdCl_2(PPh_3)_2$ (60 mg, 0.09 mmol), CuI (33 mg, 0.17 mmol), and trimethylsilylacetylene (0.26 mL, 1.88 mmol) under a N_2 atmosphere. The reaction mixture was stirred at 50 ºC for 16 h, then cooled to rt, diluted with EtOAc (15 mL) and filtered through a short pad of silica. The volatiles were removed under reduced pressure and the crude mixture purified by column chromatography (pentane:EtOAc 95:5 to 8:2) to give the product as a brown oil (457 mg, 1.61 mmol, yield = 94%).

^{1}H NMR (600 MHz, $CDCl_3$) δ 7.00 (s, 1H), 6.76 (s, 1H), 6.74 (s, 1H), 3.91 (s, 2H), 3.43 (s, 3H), 1.54 (s, 6H), 0.25 (s, 9H).

^{13}C NMR (151 MHz, $CDCl_3$) δ 145.8, 126.1, 124.1, 123.9, 118.4, 118.3, 104.4, 94.3, 91.0, 83.7, 70.9, 51.8, 28.4, 0.0.

HRMS (APCI+) *m/z* calc. for $C_{17}H_{24}NOSi$ $[M+H]^+$: 286.1622. Found: 286.1619.

---

9 Prepared according to: Pinkerton, D. MN.; Banwell, M. G.; Willis, A. C. *Org. Lett.* **2009**, *11*, 4290–4293.

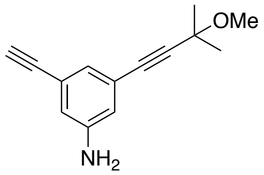

3-Ethynyl-5-(3-methoxy-3-methylbut-1-yn-1-yl)aniline (S10). TBAF (1 M solution in THF, 1.7 mL, 1.74 mmol) was added to a solution of **S9** (450 mg, 0.81 mmol) in THF (15 mL) at 0 ºC and the resulting mixture was allowed to warm to rt and stirred for 1 h. The reaction mixture was diluted with Et_2O (15 mL) and washed with brine (20 mL). The aqueous layer was extracted with Et_2O (20 mL) and the combined organic layers were dried over $MgSO_4$, filtered, and concentrated under reduced pressure. Purification by flash column chromatography (pentane:EtOAc 9:1 to 8:2) afforded the product as a yellow oil (262 mg, 0.58 mmol, yield = 72%).

^{1}H NMR (400 MHz, $CDCl_3$) δ 7.00 (t, *J* = 1.4 Hz, 1H), 6.77 (dd, *J* = 2.3, 1.4 Hz, 1H), 6.76 (dd, *J* = 2.3, 1.4 Hz, 1H), 4.06 (s, 2H), 3.41 (s, 3H), 3.01 (s, 1H), 1.52 (s, 6H).

^{13}C NMR (151 MHz, $CDCl_3$) δ 146.2, 125.8, 123.9, 123.0, 118.5, 118.3, 91.1, 83.6, 83.0, 77.0, 70.9, 51.7, 28.3.

HRMS (ESI+) *m/z* calc. for $C_{14}H_{16}NO$ $[M+H]^+$: 214.1226. Found: 214.1225.

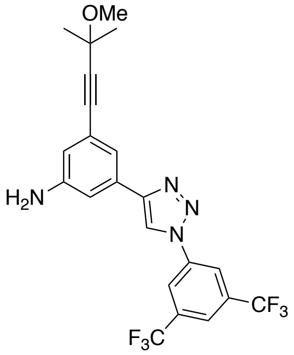

3-(1-(3,5-Bis(trifluoromethyl)phenyl)-1*H*-1,2,3-triazol-4-yl)-5-(3-methoxy-3-methylbut-1-yn-1-yl)aniline (S11). **S10** (260 mg, 1.22 mmol) and 1-azido-3,5-bis(trifluoromethyl)benzene[7] (405 mg, 1.59 mmol) were disolved in a 1:1 mixutre of CH_2Cl_2:*t*BuOH (12 mL). A solution of $CuSO_4 \cdot 5H_2O$ (15 mg, 0.06 mmol) in H_2O (6 mL) was subsequently added followed by a solution of sodium ascorbate (36 mg, 0.18 mmol) in H_2O (6 mL), and the reaction mixture was stirred at 50 ºC for 6 h. After cooling to rt CH_2Cl_2 (20 mL), an aqueous solution of KCN (10%wt, 20 mL) and a saturated aqueous solution of Na_2CO_3 (5 mL) were added. The resulting mixture was stirred at rt for 10 min and then the phases were separated and the aqueous layer was extracted with CH_2Cl_2 (2x20 mL). The combined organic layers were dried over $MgSO_4$, filtered, and concentrated under reduced pressure. Purification by flash column chromatography (pentane:EtOAc 9:1 to 1:1) afforded the product as a yellow solid (520 mg, 1.11 mmol, yield = 91%).

^{1}H NMR (400 MHz, $CDCl_3$) δ 8.30 (s, 2H), 8.28 (s, 1H), 7.97 (s, 2H), 7.31 (s, 1H), 7.30 (s, 1H), 6.79 (t, *J* = 1.8 Hz, 1H), 3.82 (s, 2H), 3.44 (s, 3H), 1.55 (s, 6H).

^{13}C NMR (151 MHz, $CDCl_3$) δ 148.8, 147.0, 138.0, 133.7 (q, *J* = 34.4 Hz), 130.4, 122.6 (q, *J* = 273.2 Hz), 124.4, 122.2 (hept, *J* = 3.7 Hz), 120.3 (q, *J* = 3.8 Hz), 119.6, 118.3, 117.4, 112.4, 91.1, 83.9, 70.9, 51.8, 28.4.

^{19}F NMR (376 MHz, $CDCl_3$) δ -63.03.

HRMS (APCI+) *m/z* calc. for $C_{22}H_{19}F_6N_4O$ $[M+H]^+$: 469.1458. Found: 469.1418.

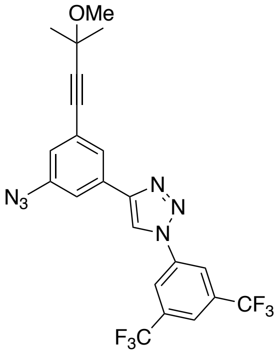

4-(3-Azido-5-(3-methoxy-3-methylbut-1-yn-1-yl)phenyl)-1-(3,5-bis(trifluoromethyl)phenyl)-1*H*-1,2,3-triazole (5d). To a stirred solution of **S10** (159 mg, 0.34 mmol) in TFA (3.4 mL) at 0 ºC was added $NaNO_2$ (28 mg, 0.41 mmol) in one portion and the reaction mixture was vigorously stirred at that temperature for 1 h. NaN_3 (33 mg, 0.51 mmol) was subsequently added and the reaction was allowed to warm to rt and stirred for an additional hour before diluting with H_2O (10 mL). The product was extracted with CH_2Cl_2 (15 mL) and the organic layer was washed with a saturated aqueous solution of $NaHCO_3$ (10 mL), brine (10 mL) and H_2O (10 mL), then dried over $MgSO_4$, filtered, and concentrated under reduced pressure. Purification by flash column chromatography (pentane:EtOAc 9:1) afforded the product as a yellow solid (163 mg, 0.33 mmol, yield = 97%).

^{1}H NMR (400 MHz, $CDCl_3$) δ 8.34 (s, 1H), 8.30 (s, 2H), 7.98 (s, 1H), 7.74 (t, *J* = 1.4 Hz, 1H), 7.56 (dd, *J* = 2.2, 1.5 Hz, 1H), 7.11 (dd, *J* = 2.2, 1.3 Hz, 1H), 3.44 (s, 3H), 1.56 (s, 6H).

^{13}C NMR (151 MHz, $CDCl_3$) δ 147.8, 141.3, 137.8, 133.8 (q, *J* = 34.4 Hz), 131.2, 125.6, 125.4, 122.4 (hept, *J* = 3.4 Hz), 122.5 (q, *J* = 273.3 Hz), 122.2, 120.4 (q, *J* = 4.0 Hz), 117.8, 116.3, 92.9, 82.7, 70.9, 51.9, 28.3.

^{19}F NMR (376 MHz, $CDCl_3$) δ -63.03.

HRMS (ESI+) *m/z* calc. for $C_{22}H_{17}F_6N_6O$ $[M+H]^+$: 495.1363. Found: 495.1357.

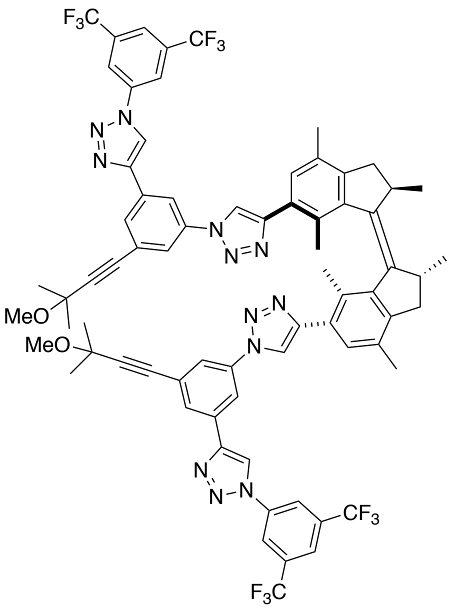

4,4'-((2*R*,2'*R*,*Z*)-2,2',4,4',7,7'-Hexamethyl-2,2',3,3'-tetrahydro-[1,1'-biindenylidene]-6,6'-diyl)bis(1-(3-(1-(3,5-bis(trifluoromethyl)phenyl)-1*H*-1,2,3-triazol-4-yl)-5-(3-methoxy-3-methylbut-1-yn-1-yl)phenyl)-1*H*-1,2,3-triazole) ((*R*,*R*)-(*P*,*P*)-*cis*-1d). (*R*,*R*)-(*P,P*)-*cis*-**4** (30 mg, 0.08 mmol) and **5d** (99 mg, 0.2 mmol) were dissolved in a 1:1 mixutre of CH_2Cl_2:*t*BuOH (2 mL). A solution of $CuSO_4 \cdot 5H_2O$ (40 mg, 0.16 mmol) in H_2O (1 mL) was subsequently added followed by a solution of sodium ascorbate (64 mg, 0.32 mmol) in H_2O (1 mL), and the reaction mixture was stirred at 70 ºC for 16 h. After cooling to rt CH_2Cl_2 (5 mL), an aqueous solution of KCN (10 wt%, 5 mL) and a saturated aqueous solution of Na_2CO_3 (2 mL) were added. The resulting mixture was stirred at rt for 10 min and then the phases were separated and the aqueous layer was extracted with CH_2Cl_2 (2x10 mL). The combined organic layers were washed with an aqueous solution of KCN (10%wt, 10 mL), and then dried over $MgSO_4$, filtered, and concentrated under reduced pressure. Purification by flash column chromatography (pentane:CH_2Cl_2:EtOAc 7:3:0 to 0:1:0 to 0:95:5) afforded the product as a yellow solid (68 mg, 0.050 mmol, yield = 62%).

^{1}H NMR (600 MHz, CD_2Cl_2) δ 8.56 (s, 2H), 8.47 (s, 2H), 8.25 (s, 4H), 8.09 (dd, *J* = 2.0, 1.5 Hz, 2H), 8.03 (t, *J* = 1.5 Hz, 2H), 7.97 (s, 2H), 7.90 (dd, *J* = 2.1, 1.4 Hz, 2H), 7.41 (s, 2H), 3.49 (p, *J* = 6.7 Hz, 2H), 3.40 (s, 6H), 3.21 (dd, *J* = 15.4, 6.5 Hz, 2H), 2.60 (d, *J* = 15.4 Hz, 2H), 2.36 (s, 6H), 1.69 (s, 6H), 1.54 (s, 12H), 1.20 (d, *J* = 6.8 Hz, 6H).

^{13}C NMR (151 MHz, CD_2Cl_2) δ 149.1, 147.0, 145.1, 142.1, 141.3, 137.7, 137.5, 133.3 (q, *J* = 34.2 Hz), 131.5, 131.3, 131.1, 129.3, 128.3, 125.6, 122.7 (q, *J* = 273.2 Hz), 122.3, 122.3 (q, *J* = 3.7 Hz), 120.2 (q, *J* = 3.9 Hz), 119.9, 119.0, 115.8, 93.7, 82.2, 70.8, 51.6, 41.2, 38.6, 27.9, 20.3, 19.9, 18.0.

^{19}F NMR (565 MHz, CD_2Cl_2) δ -63.27.

HRMS (APCI+) *m/z* calc. for $C_{72}H_{61}F_{12}N_{12}O_2$ $[M+H]^+$: 1353.4843. Found: 1353.4824.

2.3. Synthesis of motor (*R,R*)-(*P,P*)-*cis*-7

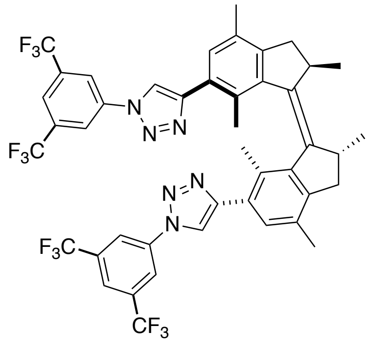

4,4'-((2*R*,2'*R*,*Z*)-2,2',4,4',7,7'-Hexamethyl-2,2',3,3'-tetrahydro-[1,1'-biindenylidene]-6,6'-diyl)bis(1-(3,5-bis(trifluoromethyl)phenyl)-1*H*-1,2,3-triazole) ((*R*,*R*)-(*P,P*)-*cis*-7). In a pressure tube, (*R,R*)-(*P,P*)-*cis*-**4** (50 mg, 0.14 mmol) and 1-azido-3,5-bis(trifluoromethyl)benzene[7] (87 mg, 0.342 mmol) were disolved in a 1:1 mixture of CH_2Cl_2:*t*BuOH (3 mL). A solution of $CuSO_4 \cdot 5H_2O$ (35 mg, 0.14 mmol) in H_2O (1.5 mL) was next added followed by a solution of sodium ascorbate (83 mg, 0.42 mmol) in H_2O (1.5 mL) and the resulting mixture was stirred at 70 ºC for a 16 h. After cooling to rt, the reaction mixture was diluted with CH_2Cl_2 (10 mL) and a 1 M aqueous solution of KCN (10 mL) was added. The mixture was stirred at rt for 30 min and then the layers were separated. The organic phase was further washed with a 1 M aqueous solution of KCN (2x10 mL), then dried over $MgSO_4$, filtered, and concentrated under reduced pressure. Purification by column chromatography (pentane:CH_2Cl_2:EtOAc 70:30:0 to 0:100:0, then 0:98:2) afforded the product as a pale yellow solid (81 mg, 0.09 mmol, yield = 66%).

^{1}H NMR (400 MHz, CD_2Cl_2) δ 8.69 (s, 2H), 8.45 (s, 4H), 7.97 (s, 2H), 7.36 (s, 2H), 3.49 (p, J = 6.7 Hz, 2H), 3.21 (dd, J = 15.5, 6.5 Hz, 2H), 2.60 (d, J = 15.4 Hz, 2H), 2.36 (s, 6H), 1.67 (s, 6H), 1.20 (d, J = 6.8 Hz, 6H).

^{13}C NMR (151 MHz, CD_2Cl_2) δ 149.5, 145.1, 142.2, 141.3, 138.3, 133.1 (q, J = 34.2 Hz), 131.5, 131.2, 129.3, 128.0, 122.8 (q, J = 272.9 Hz), 121.8 (hept, J = 4.4 Hz), 120.4 (q, J = 3.4 Hz), 120.4, 41.1, 38.7, 20.2, 19.9, 18.0.

^{19}F NMR (565 MHz, CD_2Cl_2) δ -63.37.

HRMS (ESI+) *m/z* calc. for $C_{44}H_{35}F_{12}N_6$ $[M+H]^+$: 875.2726. Found: 875.2727.

2.4. Synthesis of motor (*R*,*R*)-(*P*,*P*)-*trans*-**1b**

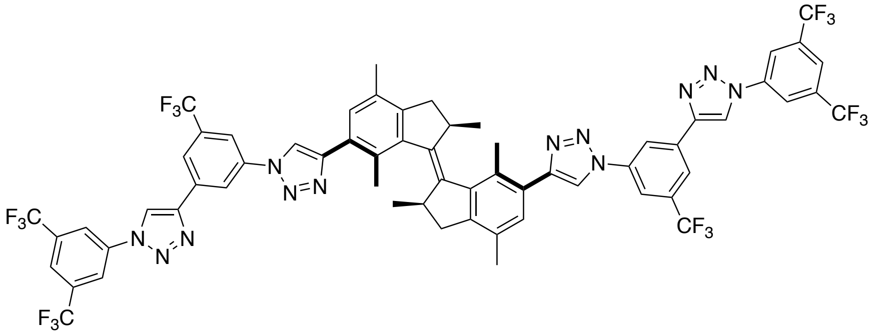

4,4'-((2*R*,2'*R*,*E*)-2,2',4,4',7,7'-Hexamethyl-2,2',3,3'-tetrahydro-[1,1'-biindenylidene]-6,6'-diyl)bis(1-(3-(1-(3,5-bis(trifluoromethyl)phenyl)-1*H*-1,2,3-triazol-4-yl)-5-(trifluoromethyl)phenyl)-1*H*-1,2,3-triazole) ((*R*,*R*)-(*P*,*P*)-*trans*-1b). In a pressure tube, (*R*,*R*)-(*P*,*P*)-*trans*-**4** (50 mg, 0.14 mmol) and **5b** (87 mg, 0.342 mmol) were dissolved in a 1:1 mixture of CH_2Cl_2:*t*BuOH (3 mL). A solution of $CuSO_4 \cdot 5H_2O$ (35 mg, 0.14 mmol) in H_2O (1.5 mL) was next added followed by a solution of sodium ascorbate (83 mg, 0.42 mmol) in H_2O (1.5 mL) and the resulting mixture was stirred at 70 ºC for a 16 h. After cooling to rt, the reaction mixture was diluted with CH_2Cl_2 (10 mL) and a 1 M aqueous solution of KCN (10 mL) was added. The mixture was stirred at rt for 30 min and then the layers were separated. The organic phase was further washed with a 1 M aqueous solution of KCN (2x10 mL), then dried over $MgSO_4$, filtered, and concentrated under reduced pressure. Purification by column chromatography (pentane:CH_2Cl_2:EtOAc 70:30:0 to 0:100:0, then 0:98:2) afforded the product as a white solid (94 mg, 0.073 mmol, yield = 52%).

^{1}H NMR (400 MHz, CD_2Cl_2) δ 8.73 (s, 1H), 8.68 (s, 1H), 8.41 (s, 2H), 8.40 (s, 1H), 8.30 (s, 1H), 8.21 (s, 1H), 8.07 (s, 1H), 7.61 (s, 1H), 3.07 (p, *J* = 6.4 Hz, 1H), 2.79 (dd, *J* = 14.7, 5.7 Hz, 1H), 2.65 (s, 3H), 2.40 (d, *J* = 14.9 Hz, 1H), 2.32 (s, 3H), 1.21 (d, *J* = 6.6 Hz, 3H).

^{13}C NMR (151 MHz, CD_2Cl_2) δ 148.9, 146.7, 143.4, 142.5, 142.1, 138.4, 137.8, 133.5 (q, *J* = 34.4 Hz), 133.1 (q, *J* = 33.4 Hz), 132.7, 132.1, 129.2, 129.0, 128.1, 123.4 (d, *J* = 272.7 Hz), 122.7 (hept, *J* = 3.7 Hz), 122.2 (d, *J* = 4.1 Hz), 122.7 (q, *J* = 273.0 Hz), 120.7 (q, *J* = 4.2, 3.8 Hz), 120.5, 119.9, 119.1, 117.1 (q, *J* = 3.8 Hz), 42.4, 39.0, 21.4, 18.7, 18.0.

^{19}F NMR (376 MHz, CD_2Cl_2) δ -63.19 (s, 6H), -63.38 (s, 12H).

HRMS (ESI+) *m/z* calc. for $C_{62}H_{41}F_{18}N_{12}$ $[M]^+$: 1295.3284. Found: 1295.3285.

Recrystallization from pentane at -25 ºC afforded the enantiopure product (*R*,*R*)-(*P*,*P*)-*trans*-**1b** (e.r. > 99:1). SFC analysis: Chiralpak IB (70% CO_2:30% MeOH), 160 bar, 40 °C, 3 mL/min, t_1 = 16.5 min, t_2 = 19.5 min.

3. Isomerization of motor 1b

3.1. UV-vis irradiation studies

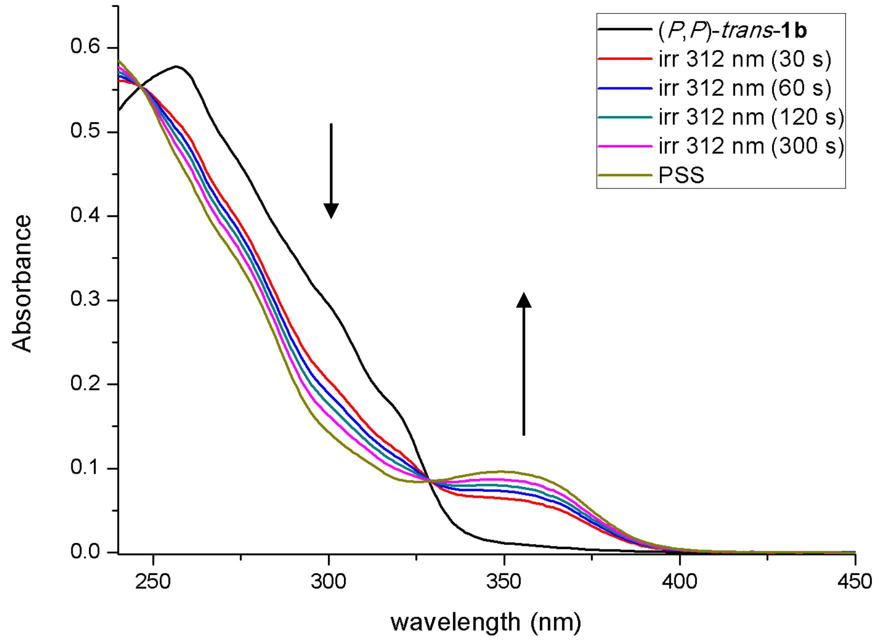

Figure S1. UV-vis spectral changes of (*P,P*)-*trans*-**1b** (10^{-5} M in THF) upon irradiation with 312 nm light at 20 ºC.

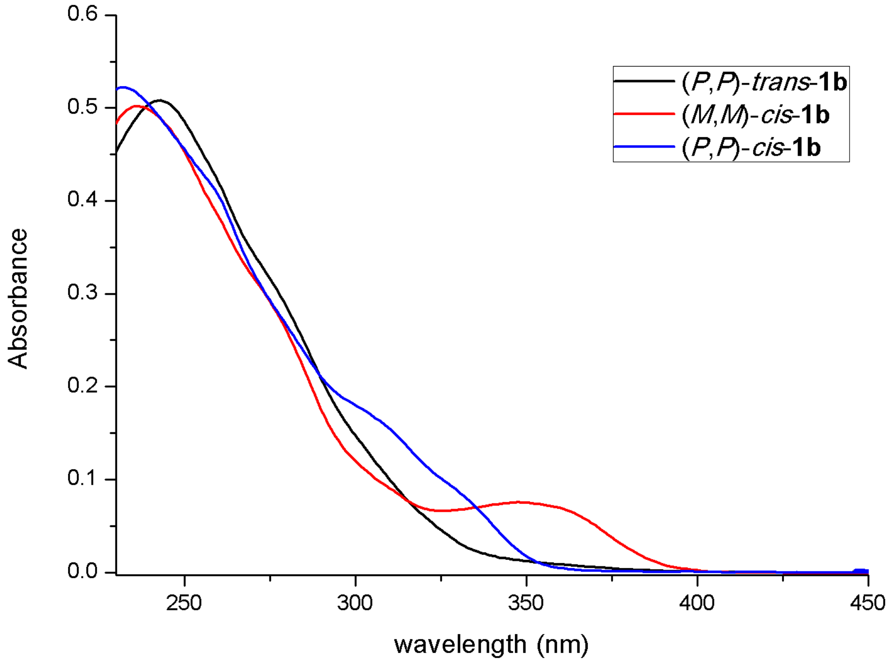

Figure S2. UV-vis spectra of (*P,P*)-*trans*-**1b**, (*M,M*)-*cis*-**1b**, and (*P,P*)-*cis*-**1b** (10^{-5} M in THF) at 10 ºC.

3.2. NMR irradiation studies

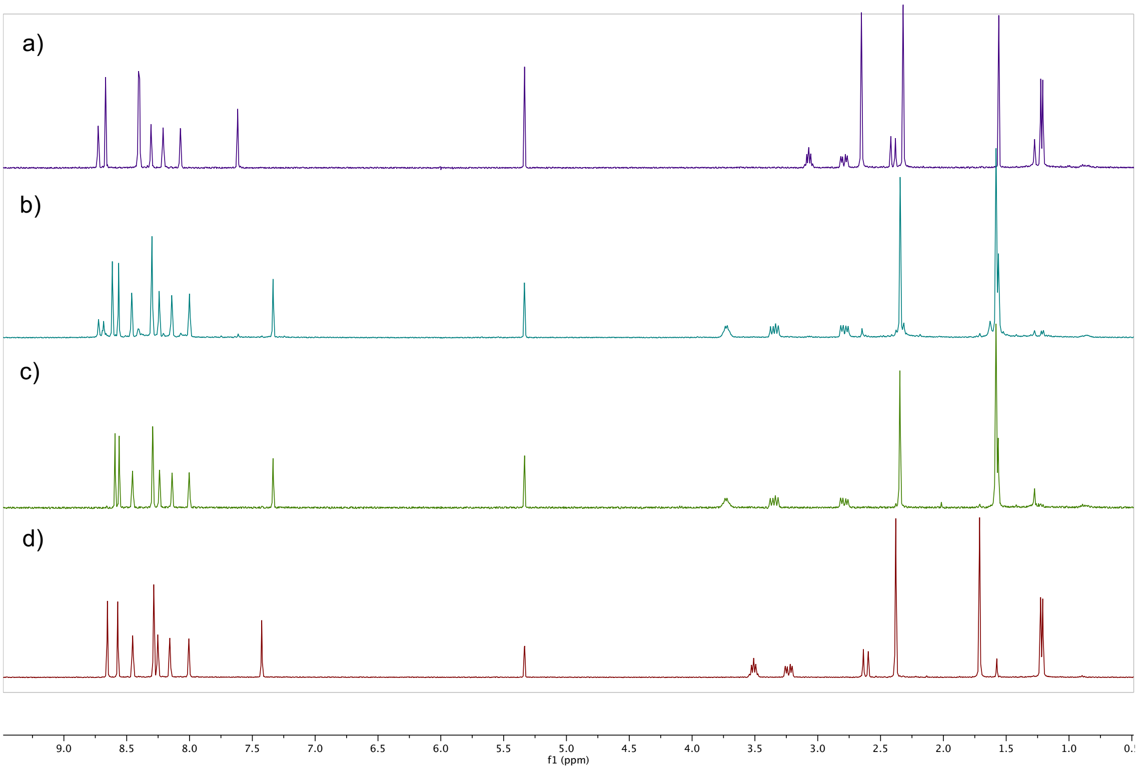

Figure S3. ^{1}H NMR spectra (CD_2Cl_2) of a) (*P,P*)-*trans*-**1b**; b) (*M,M*)-*cis*-**1b**:(*P,P*)-*trans*-**1b** 90:10 obtained upon irradiation of a solution of (*P,P*)-*trans*-**1b** in THF with 312 nm light at 5 ºC; c) (*M,M*)-*cis*-**1b** after purification of the mixture in b) by prepTLC; and d) (*P,P*)-*cis*-**1b** after thermal helix inversion upon heating at 60 ºC for 16 h in THF.

3.3. Kinetic studies on the THI step

A solution of (*P*,*P*)-*trans*-**1b** in degassed THF (10^{-5} M) in a 1 cm cuvette was irradiated to PSS with 312 nm light. The rate of the thermal relaxation was followed by monitoring the absorbance at 360 nm at 5 different temperatures (50, 55, 60, 65, 70 ºC).

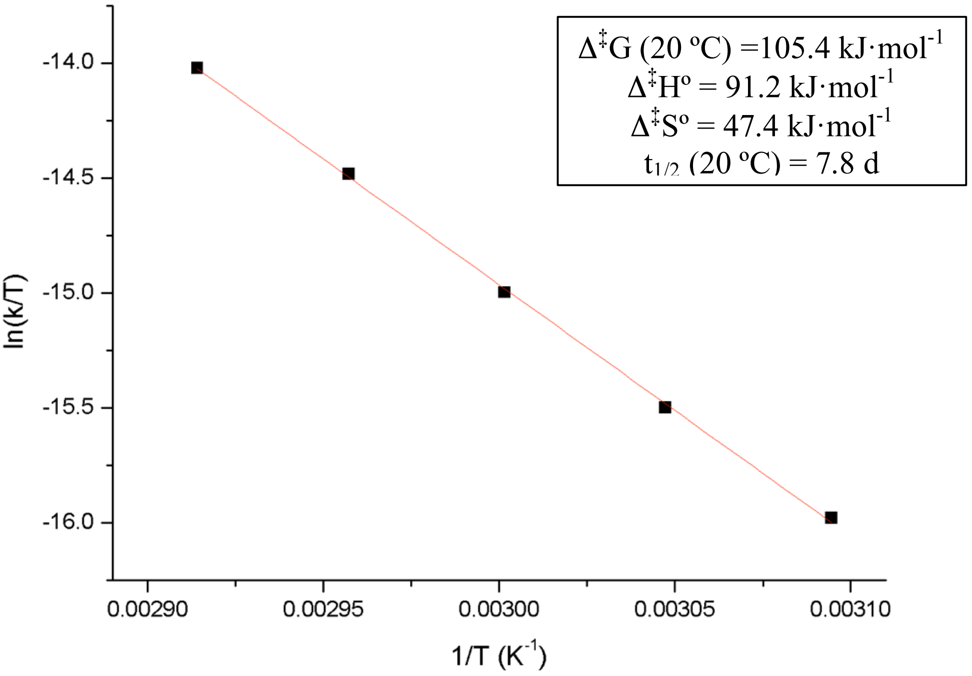

Figure S4. Eyring plot of the THI of (*M*,*M*)-*cis*-**1b** to (*P*,*P*)-*cis*-**1b**.

4. Job Plot analysis of motor 1b with TBACl

All titration data were obtained by UV-vis spectroscopy. A stock solution of the corresponding isomer of **1b** ($2\cdot10^{-5}$ M in THF) was titrated with a stock solution of TBACl ($2\cdot10^{-5}$ M in THF) keeping a constant [**1b**] + [TBACl] = 10^{-5} M.

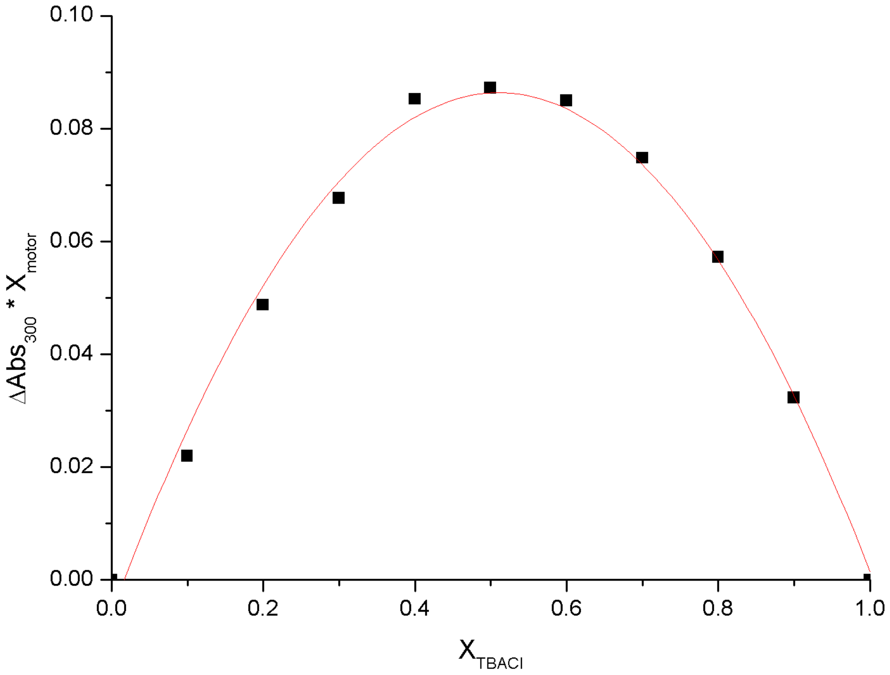

Figure S5. Job plot analysis of (*P*,*P*)-*cis*-**1b** with TBACl. The position of the maximum reveals a 1:1 stoichiometry.

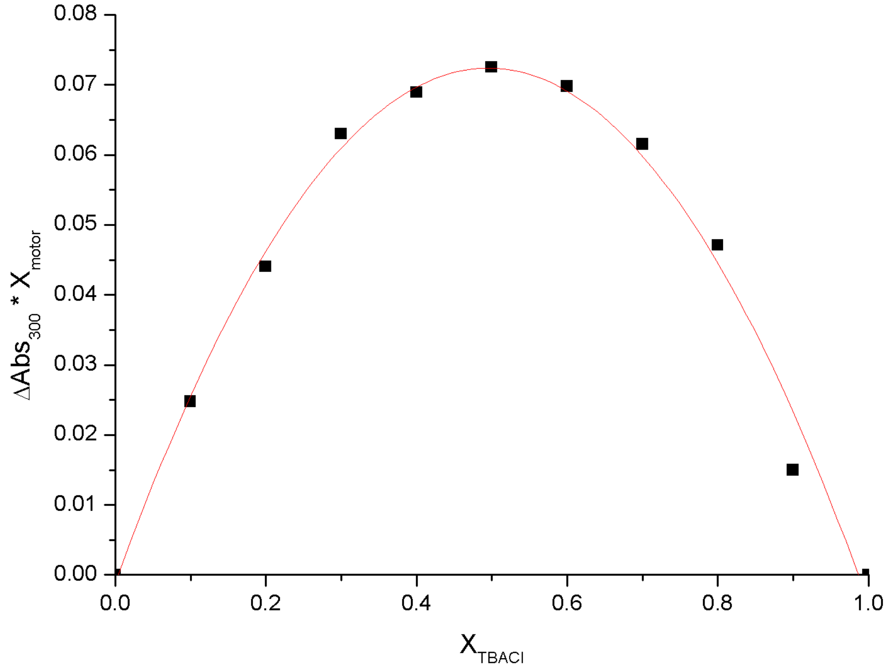

Figure S6. Job plot analysis of (*M*,*M*)-*cis*-**1b** with TBACl. The position of the maximum reveals a 1:1 stoichiometry.

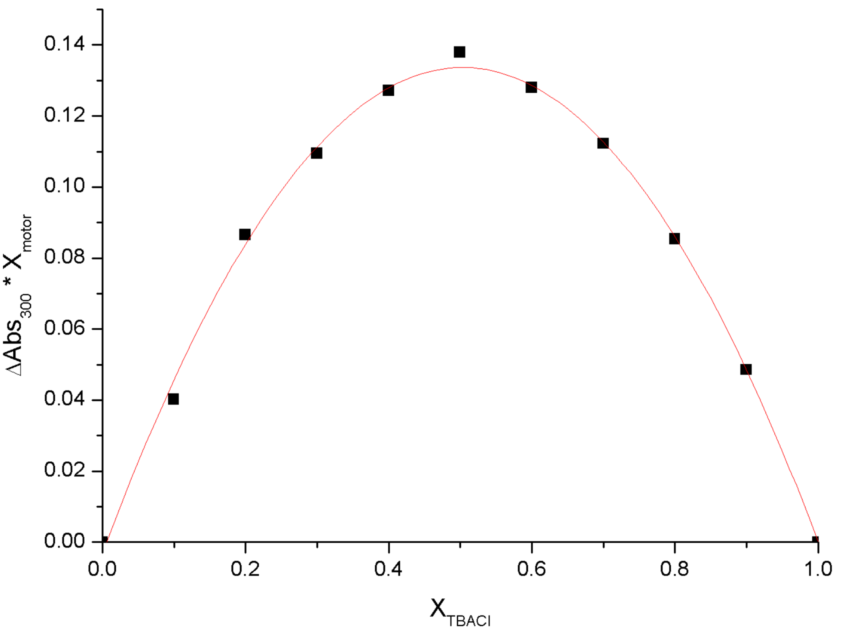

Figure S7. Job plot analysis of (*P*,*P*)-*trans*-**1b** with TBACl. The position of the maximum reveals a 1:1 stoichiometry.

5. Catalysis experiments

1-Cloroisochroman (**8a**),[10] 1-methoxyisochroman (**11a**),[11] 1-methoxy-5-methylisochroman (**11b**),[11] 1-methoxy-7-methylisochroman (**11c**),[11] 7-fluoro-1-methoxyisochroman (**11d**),[12] 7-chloro-1-methoxyisochroman (**11e**),[12] and 4-methoxy-1,4-dihydro-2*H*-benzo[*f*]isochroman (**11f**)[13] were prepared according to literature procedures.

*5.1. Optimization of the addition of nucleophiles to **8a***

Table S1. Influence of the solvent.

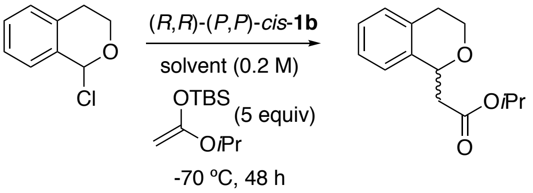

entry	solvent	NMR conversion to product (%)	e.r. (210 nm)
1	MTBE	71	86:14
2	THF	20	50:50
3	Et_2O	32	64:36
4	*t*AmylOMe	81	85:15
5	CH_2Cl_2	32	54:46
6	toluene	30	78:22

Table S2. Influence of the nucleophile.

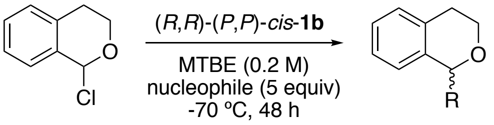

entry	nucleophile	NMR conversion to product (%)	e.r. (210 nm)
1	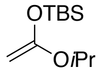	71	86:14
2	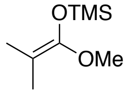	67	75:25
3	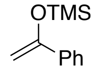	0	-
4	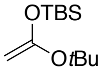	66	85:15
5	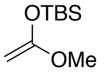	70	77:23

---

10 Berkessel, A.; Das, S.; Pekel, D.; Neudörfl, J.-M. *Angew. Chem. Int. Ed.* **2014**, *53*, 11660–11664.

11 Reisman, S. E.; Doyle, A. G.; Jacobsen, E. N. *J. Am. Chem. Soc.* **2008**, *130*, 7198–7199.

12 Li, Z.; Yu, H.; Liu, Y.; Zhou, L.; Sun, Z.; Guo, H. *Adv. Synth. Catal.* **2016**, *358*, 1880–1885.

13 Maity, P.; Srinivas, H. D.; Watson, M. P. *J. Am. Chem. Soc.* **2011**, *133*, 17142–17145.

*5.2. General procedure for the addition of **9a** to **8***

An oven-dried Schlenk flask was charged with the corresponding 1-methoxyisochroman derivative **11** (0.1 mmol) and dry CH_2Cl_2 (0.15 mL) under N_2 atmosphere. The solution was cooled to 0 ºC and BCl_3 (1.0 M solution in hexane, 45 µL, 0.045 mmol) was added dropwise. The reaction mixture was allowed to warm to rt and stirred for 1.5 h, which led to quantitative formation of the corresponding 1-chloroisochroman derivative. The volatiles were removed under reduced pressure, then anhydrous MTBE (0.1 mL) was added and the volatiles were removed again under reduced pressure. The resulting crude 1-chloroisochroman **8** was dissolved in anhydrous MTBE (0.4 mL) and the solution cooled to -80 ºC followed by the addition of a solution of the required isomer of motor **1b** (0.01 mmol) in anhydrous MTBE (0.27 mL). After stirring at -80 ºC for 5 min, **9a** (0.5 mmol) was added dropwise and the resulting mixture was stirred at that temperature for 5 days. The reaction was quenched by the addition of a sodium methoxide solution in methanol (30 wt.%, 10 equiv) at -80 ºC, which caused the quantitative conversion of the unreacted 1-chloroisochroman into 1-methoxyisochroman. The mixture was diluted with a 1:1 mixture of pentane:Et_2O (2 mL) and then filtered through a short plug of silica. Removal of the volatiles under reduced pressure afforded the crude product, which was purified by column chromatography (pentane:Et_2O 95:5 to 9:1).

Note 1: The conversions during the optimization studies were calculated from the crude ^{1}H NMR spectrum with respect to the remaining 1-chloroisochroman, which appears as methyl acetal after quenching.

Note 2: (*R*,*R*)-(*P*,*P*)-*trans*-**1b** was used as obtained from the synthetic sequence. (*R*,*R*)-(*M*,*M*)-*cis*-**1b** was obtained by irradiation of (*R*,*R*)-(*P*,*P*)-*trans*-**1b** with 312 nm light in degassed THF for 3 h followed by purification by prep-TLC eluted with CH_2Cl_2:EtOAc 98:2. (*R*,*R*)-(*P*,*P*)-*cis*-**1b** obtained by heating of a solution of (*R*,*R*)-(*M*,*M*)-*cis*-**1b** in THF at 60 ºC for 16 h, and the one obtained directly from the synthesis provided comparable results.

*5.3. Characterization of products **10***

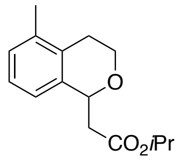

Isopropyl 2-(5-methylisochroman-1-yl)acetate (10b). Clear oil, yield = 53% (50:50 e.r.) with (*R*,*R*)-(*P*,*P*)-*trans*-**1b**, yield = 41% (65:35 e.r.) with (*R*,*R*)-(*M*,*M*)-*cis*-**1b**, and yield = 81% (14:86 e.r.) with (*R*,*R*)-(*P*,*P*)-*cis*-**1b**. HPLC analysis: OD-H, 40 ºC, 1.0 mL/min, heptane:IPA = 98:2, t_1 = 8.2 min, t_2 = 9.2 min.

^{1}H NMR (600 MHz, $CDCl_3$) δ 7.02 (t, *J* = 7.5 Hz, 1H), 6.98 (d, *J* = 6.9 Hz, 1H), 6.83 (d, *J* = 7.6 Hz, 1H), 5.16 (dd, *J* = 9.5, 3.6 Hz, 1H), 5.02 (hept, *J* = 6.3 Hz, 1H), 4.08 (ddd, *J* = 11.5, 5.4, 4.5 Hz, 1H), 3.77 (ddd, *J* = 11.5, 8.6, 4.4 Hz, 1H), 2.77 (dd, *J* = 15.1, 3.7 Hz, 1H), 2.71 (ddd, *J* = 16.4, 8.6, 5.5 Hz, 1H), 2.66 (dd, *J* = 15.1, 9.5 Hz, 1H), 2.54 (dt, *J* = 16.5, 4.5 Hz, 1H), 2.16 (s, 3H), 1.19 (d, *J* = 6.2 Hz, 3H), 1.18 (d, *J* = 6.2 Hz, 3H).

^{13}C NMR (151 MHz, $CDCl_3$) δ 170.9, 136.8, 136.5, 132.5, 128.0, 125.8, 122.2, 73.2, 68.0, 62.8, 42.1, 26.4, 21.9, 21.8, 19.1.

HRMS (ESI+) *m/z* calc. for $C_{20}H_{15}O_3Na$ $[M+Na]^+$: 271.1305. Found: 271.1305.

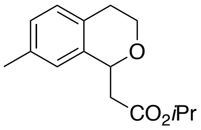

Isopropyl 2-(7-methylisochroman-1-yl)acetate (10c). Clear oil, yield = 58% (50:50 e.r.) with (*R*,*R*)-(*P*,*P*)-*trans*-**1b**, yield = 45% (80:20 e.r.) with (*R*,*R*)-(*M*,*M*)-*cis*-**1b**, and yield = 82% (9:91 e.r.) with (*R*,*R*)-(*P*,*P*)-*cis*-**1b**. HPLC analysis: OD-H, 40 ºC, 0.5 mL/min, heptane:IPA = 99:1, t_1 = 18.9 min, t_2 = 19.8 min.

^{1}H NMR (600 MHz, $CDCl_3$) δ 7.02 (t, *J* = 7.5 Hz, 1H), 6.98 (d, *J* = 7.4 Hz, 1H), 6.84 (d, *J* = 7.6 Hz, 1H), 5.16 (dd, *J* = 9.5, 3.6 Hz, 1H), 5.02 (hept, *J* = 6.3 Hz, 1H), 4.09 (ddd, *J* = 11.5, 5.4, 4.5 Hz, 1H), 3.77 (ddd, *J* = 11.5, 8.6, 4.3 Hz, 1H), 2.77 (dd, *J* = 15.1, 3.7 Hz, 1H), 2.72 (ddd, *J* = 16.4, 8.6, 5.5 Hz, 1H), 2.66 (dd, *J* = 15.1, 9.5 Hz, 1H), 2.55 (dt, *J* = 16.5, 4.5 Hz, 1H), 2.17 (s, 3H), 1.19 (d, *J* = 6.2 Hz, 3H), 1.18 (d, *J* = 6.2 Hz, 3H).

^{13}C NMR (151 MHz, $CDCl_3$) δ 170.9, 136.8, 136.5, 132.5, 128.0, 125.8, 122.2, 73.2, 68.0, 62.8, 42.1, 26.4, 21.9, 21.8, 19.1.

HRMS (ESI+) *m/z* calc. for $C_{20}H_{15}O_3Na$ $[M+Na]^+$: 271.1305. Found: 271.1305.

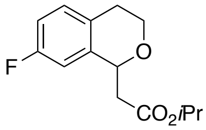

Isopropyl 2-(7-fluoroisochroman-1-yl)acetate (10d). Clear oil, yield = 50% (50:50 e.r.) with (*R*,*R*)-(*P*,*P*)-*trans*-**1b**, and yield = 50% (25:75 e.r.) with (*R*,*R*)-(*P*,*P*)-*cis*-**1b**. HPLC analysis: OD-H, 40 ºC, 0.5 mL/min, heptane:IPA = 99:1, t_1 = 17.8 min, t_2 = 18.8 min.

^{1}H NMR (600 MHz, $CDCl_3$) δ 7.01 (dd, *J* = 8.4, 5.7 Hz, 1H), 6.81 (tdd, *J* = 8.4, 2.7, 0.8 Hz, 1H), 6.69 (dd, *J* = 9.5, 2.6 Hz, 1H), 5.11 (dd, *J* = 8.7, 4.1 Hz, 1H), 5.02 (hept, *J* = 6.3 Hz, 1H), 4.05 (ddd, *J* = 11.4, 5.2, 4.2 Hz, 1H), 3.72 (ddd, *J* = 11.4, 9.0, 4.0 Hz, 1H), 2.89 – 2.81 (m, 1H), 2.73 (dd, *J* = 15.2, 4.0 Hz, 1H), 2.66 (dd, *J* = 15.2, 8.9 Hz, 1H), 2.61 (dt, *J* = 16.1, 4.1 Hz, 1H), 1.19 (d, *J* = 2.6 Hz, 3H), 1.18 (d, *J* = 2.6 Hz, 3H).

^{13}C NMR (151 MHz, $CDCl_3$) δ 170.5, 161.2 (d, *J* = 244.2 Hz), 138.7 (d, *J* = 6.2 Hz), 130.5 (d, *J* = 7.6 Hz), 129.4 (d, *J* = 3.1 Hz), 113.9 (d, *J* = 21.3 Hz), 111.2 (d, *J* = 22.0 Hz), 72.8 (d, *J* = 2.0 Hz), 68.2, 63.2, 41.8, 28.1, 21.8, 21.8.

^{19}F NMR (565 MHz, $CDCl_3$) δ -115.77 – -116.01 (m).

HRMS (ESI+) *m/z* calc. for $C_{14}H_{17}FO_3Na$ $[M+Na]^+$: 275.1054. Found: 275.1054.

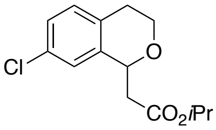

Isopropyl 2-(7-chloroisochroman-1-yl)acetate (10e). Clear oil, yield = 42% (50:50 e.r.) with (*R*,*R*)-(*P*,*P*)-*trans*-**1b**, and yield = 50% (5:95 e.r.) with (*R*,*R*)-(*P*,*P*)-*cis*-**1b**. HPLC analysis: OD-H, 40 ºC, 0.5 mL/min, heptane:IPA = 99:1, t_1 = 18.6 min, t_2 = 20.0 min.

^{1}H NMR (600 MHz, $CDCl_3$) δ 7.05 (ddd, *J* = 8.2, 2.2, 0.8 Hz, 1H), 6.98 – 6.94 (m, 2H), 5.07 (dd, *J* = 8.9, 3.7 Hz, 1H), 4.99 (hept, *J* = 6.3 Hz, 1H), 4.01 (ddd, *J* = 11.5, 5.3, 4.2 Hz, 1H), 3.68 (ddd, *J* = 11.4, 9.0, 4.0 Hz, 1H), 2.82 (dddt, *J* = 16.5, 9.1, 5.3, 1.2 Hz, 1H), 2.72 (dd, *J* = 15.2, 3.9 Hz, 1H), 2.63 (dd, *J* = 15.2, 9.0 Hz, 1H), 2.58 (dt, *J* = 16.4, 4.2 Hz, 1H), 1.15 (d, *J* = 6.3 Hz, 6H).

^{13}C NMR (151 MHz, $CDCl_3$) δ 170.4, 138.7, 132.4, 131.8, 130.4, 126.9, 124.7, 72.7, 68.2, 63.0, 41.8, 28.2, 21.8, 21.8.

HRMS (ESI+) *m/z* calc. for $C_{14}H_{17}ClO_3Na$ $[M+Na]^+$: 291.0758. Found: 291.0759.

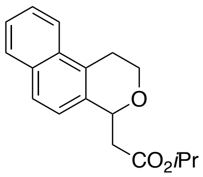

Isopropyl 2-(1,4-dihydro-2*H*-benzo[*f*]isochromen-4-yl)acetate (10f). Clear oil, yield = 36% (50:50 e.r.) with (*R*,*R*)-(*P*,*P*)-*trans*-**1b**, and yield = 60% (18:82 e.r.) with (*R*,*R*)-(*P*,*P*)-*cis*-**1b**. HPLC analysis: OD-H, 40 ºC, 0.5 mL/min, heptane:IPA = 98:2, t_1 = 21.4 min, t_2 = 23.1 min.

^{1}H NMR (600 MHz, $CDCl_3$) δ 7.87 (d, *J* = 8.4 Hz, 1H), 7.75 (dd, *J* = 8.1, 1.4 Hz, 1H), 7.62 (d, *J* = 8.5 Hz, 1H), 7.47 (ddd, *J* = 8.3, 6.7, 1.4 Hz, 1H), 7.42 (ddd, *J* = 8.1, 6.7, 1.2 Hz, 1H), 7.11 (d, *J* = 8.6 Hz, 1H), 5.32 (dd, *J* = 10.5, 3.0 Hz, 1H), 5.04 (hept, *J* = 6.3 Hz, 1H), 4.22 (dt, *J* = 11.6, 5.0 Hz, 1H), 3.90 (ddd, *J* = 11.9, 8.0, 4.4 Hz, 1H), 3.19 – 3.12 (m, 1H), 3.04 (dt, *J* = 16.4, 4.1 Hz, 1H), 2.86 (dd, *J* = 15.2, 3.5 Hz, 1H), 2.71 (dd, *J* = 15.2, 9.6 Hz, 1H), 1.20 (d, *J* = 4.2 Hz, 3H), 1.19 (d, *J* = 4.2 Hz, 3H).

^{13}C NMR (151 MHz, $CDCl_3$) δ 170.8, 133.8, 132.2, 131.9, 129.4, 128.5, 126.6, 126.5, 125.7, 122.8, 122.8, 73.2, 68.1, 62.4, 41.9, 25.4, 21.9, 21.8.

HRMS (ESI+) *m/z* calc. for $C_{18}H_{20}O_3Na$ $[M+Na]^+$: 307.1305. Found: 307.1310.

*5.4. Asignment of absolute configuration of products **10***

The absolute configuration of **10a** obtained using (*R*,*R*)-(*P*,*P*)-*cis*-**1b** as the catalyst was determined following the procedure described by Jacobsen and coworkers.[11] Comparison of the optical rotation of the saponification product with the values reported in the literature led to the assignment of the preferred enantiomer as (*S*)-**10a**, and consequently (*R*)-**10a** was assigned as the major enantiomer formed in the presence of (*R*,*R*)-(*M*,*M*)-*cis*-**1b**.

6. NMR spectra

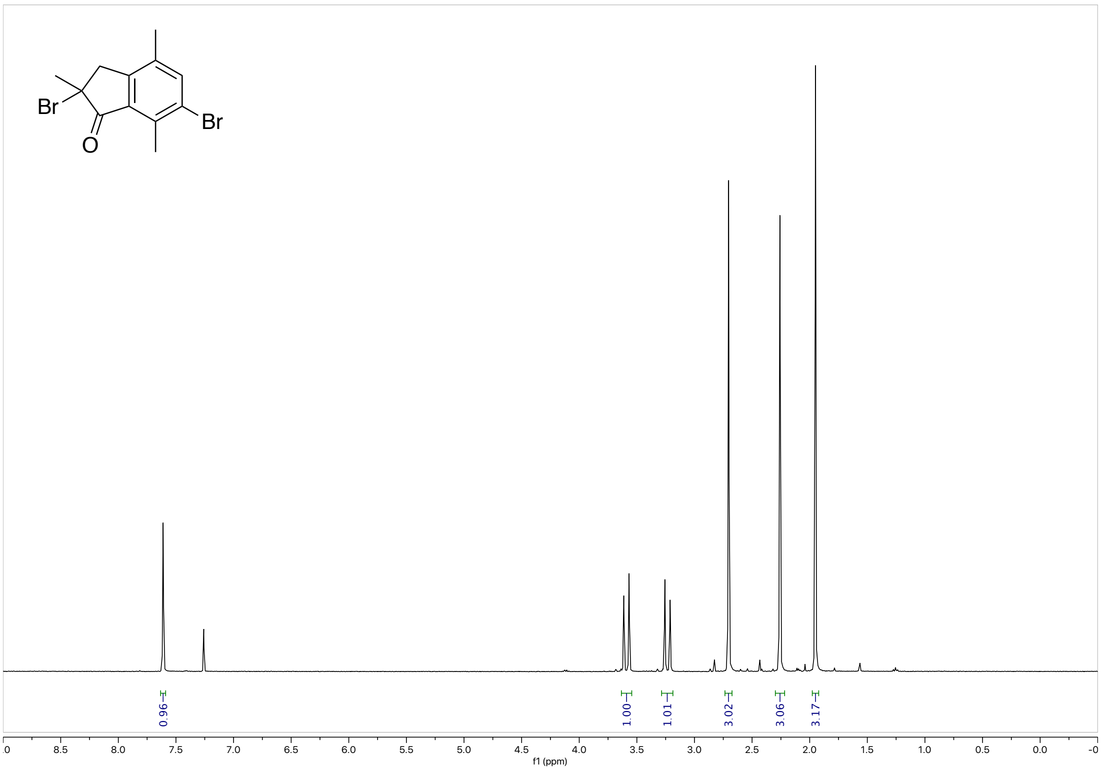

Figure S8. ^{1}H NMR (400 MHz, $CDCl_3$) of **S1**.

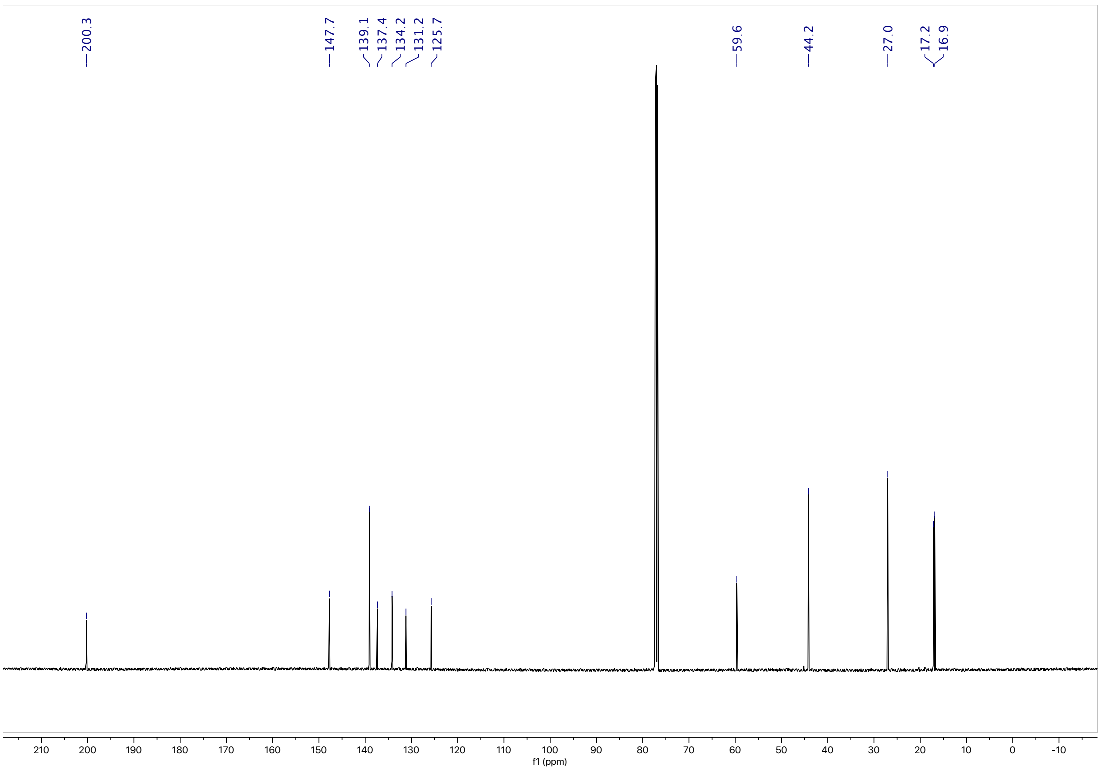

Figure S9. ^{13}C NMR (151 MHz, $CDCl_3$) of **S1**.

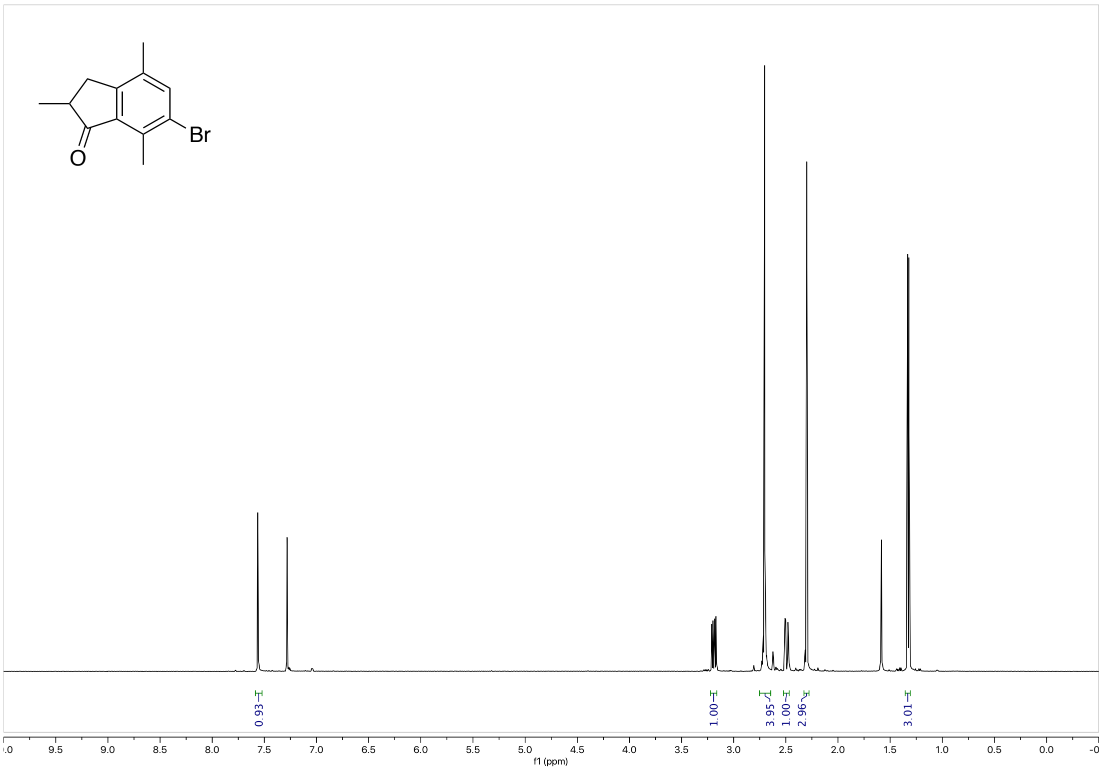

Figure S10. ^{1}H NMR (400 MHz, $CDCl_3$) of **S2**.

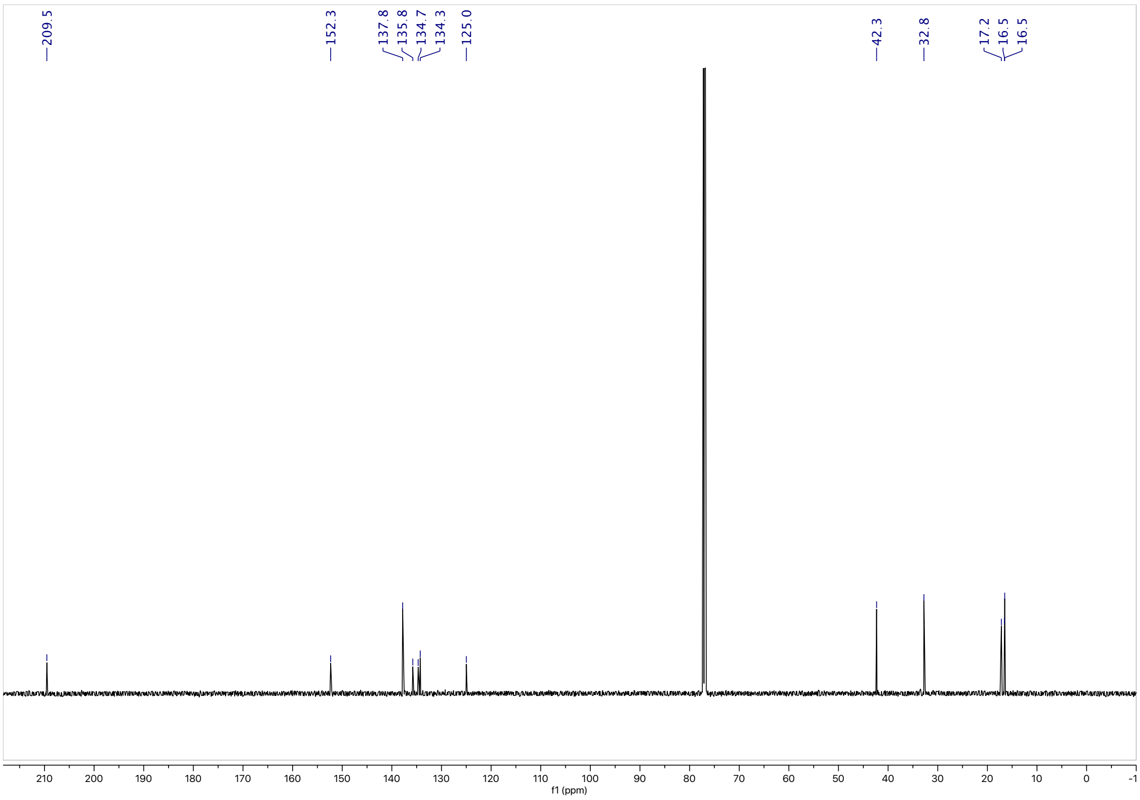

Figure S11. ^{13}C NMR (101 MHz, $CDCl_3$) of **S2**.

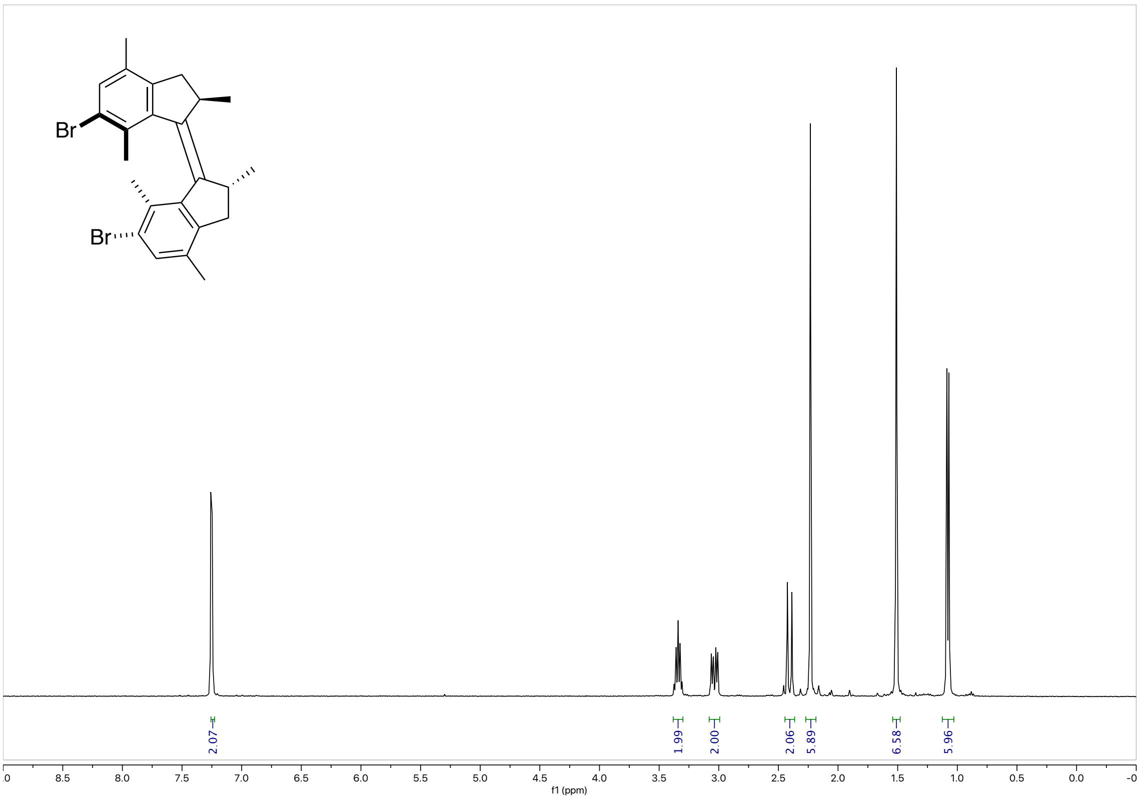

Figure S12. ^{1}H NMR (400 MHz, $CDCl_3$) of ***(P,P)-cis*-2**.

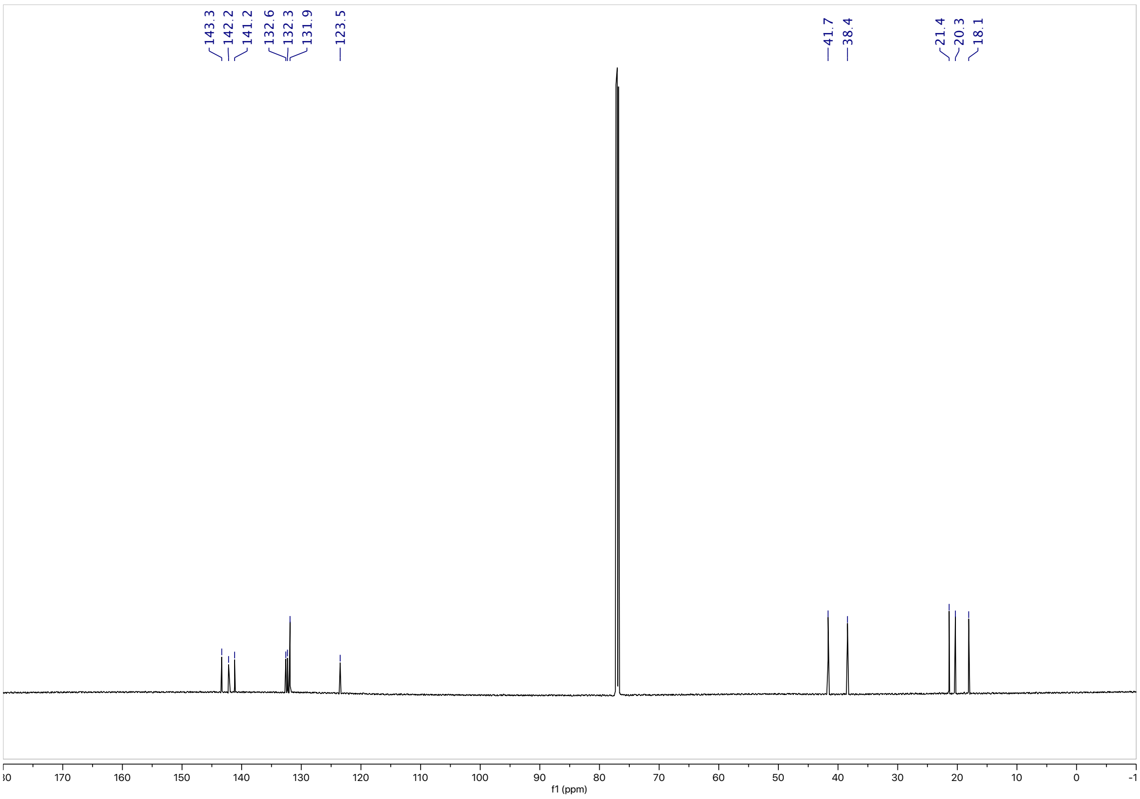

Figure S13. ^{13}C NMR (151 MHz, $CDCl_3$) of ***(P,P)-cis*-2**.

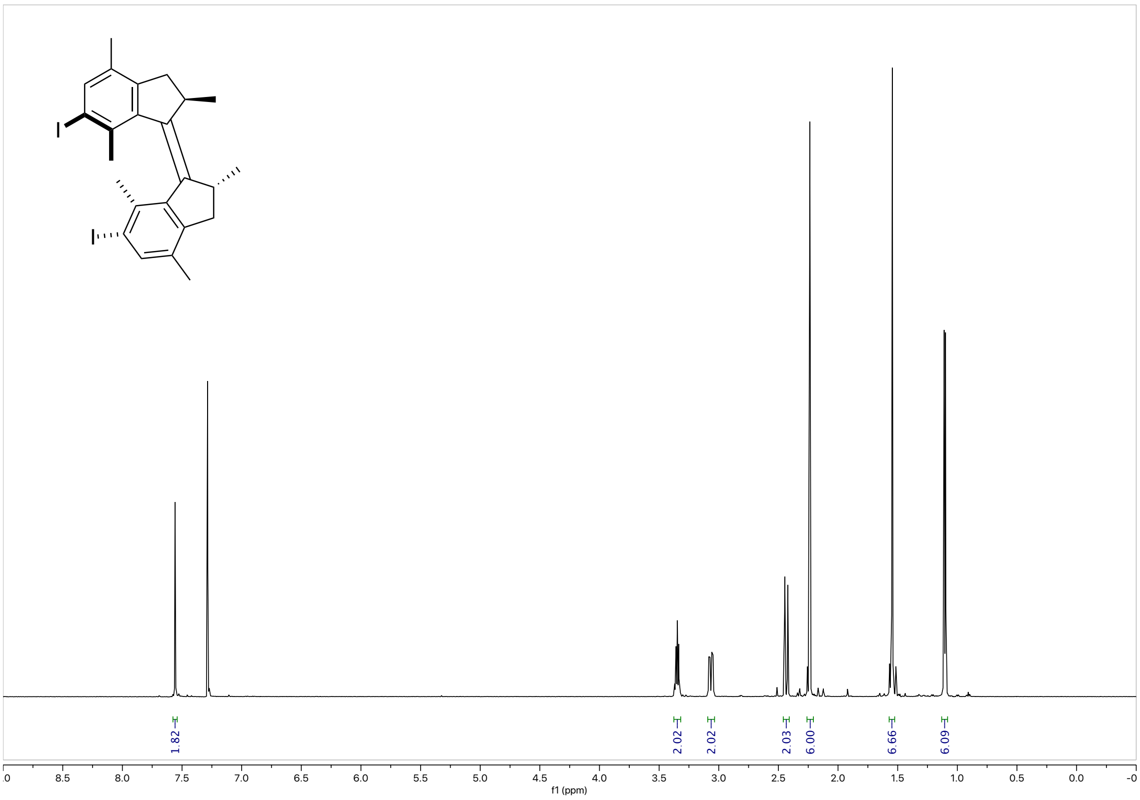

Figure S14. ^{1}H NMR (600 MHz, $CDCl_3$) of ***(P,P)-cis*-3**.

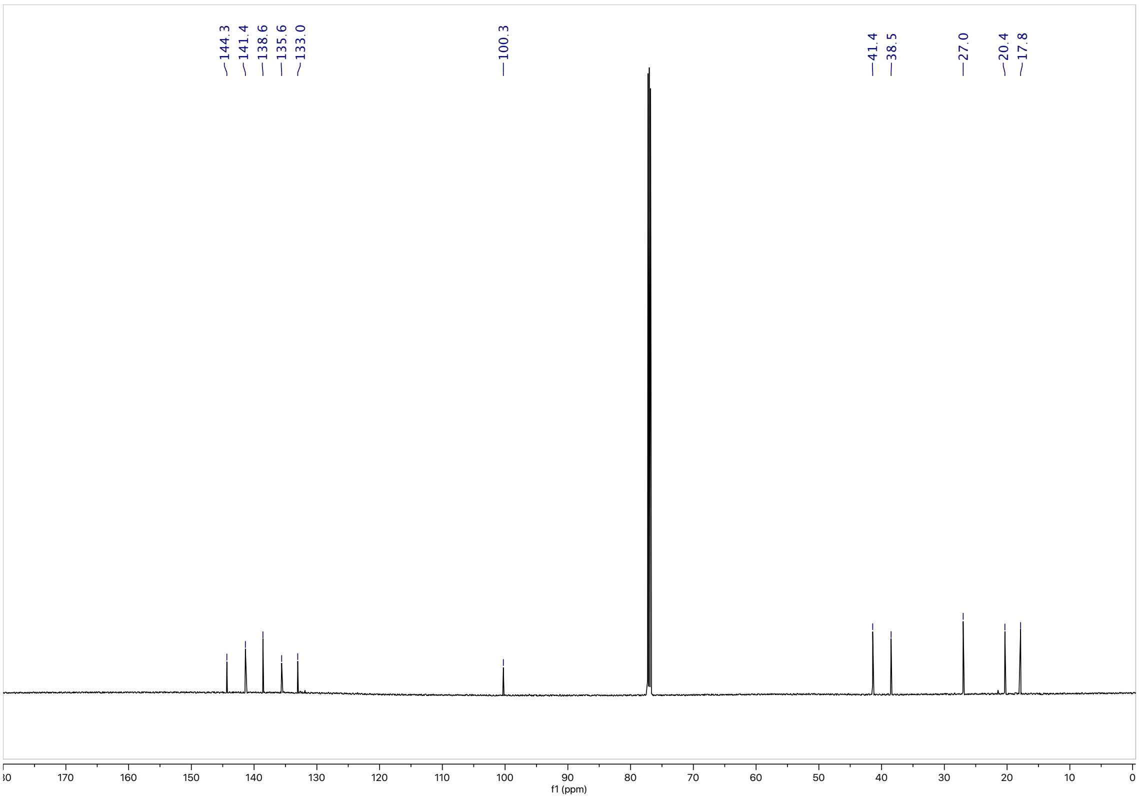

Figure S15. ^{13}C NMR (151 MHz, $CDCl_3$) of ***(P,P)-cis*-3**.

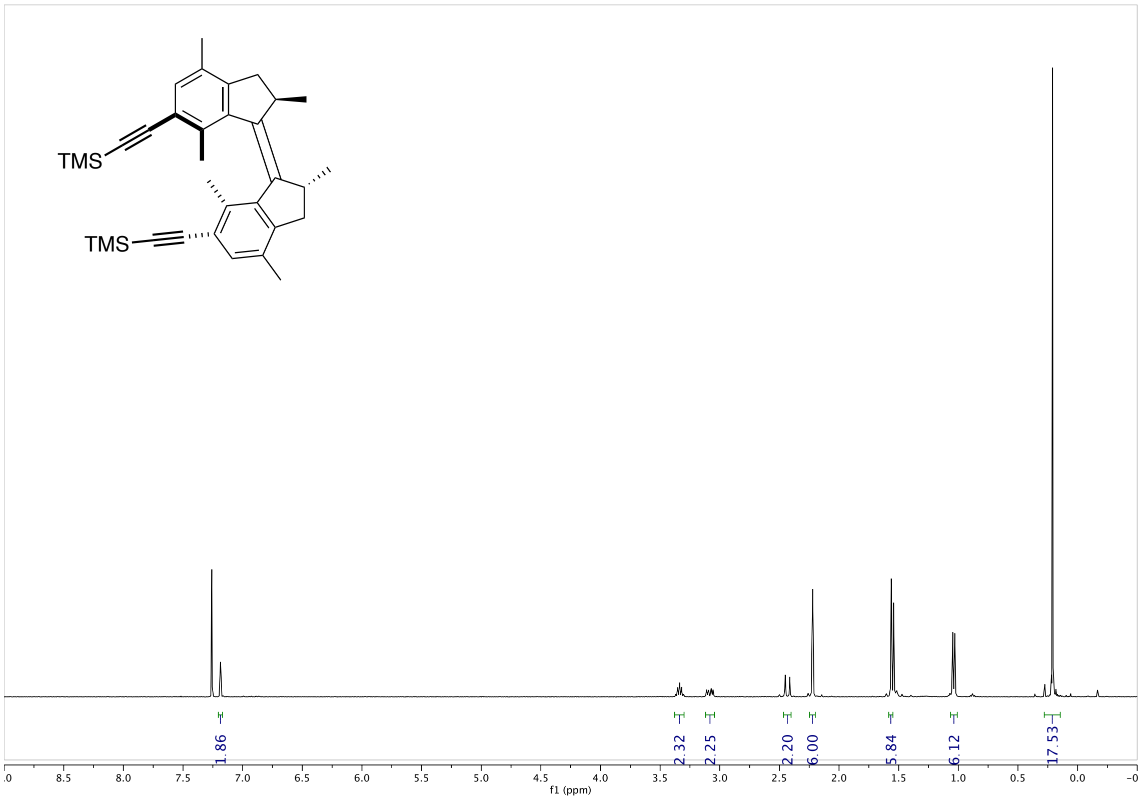

Figure S16. ^{1}H NMR (400 MHz, $CDCl_3$) of ***(P,P)-cis*-S3**.

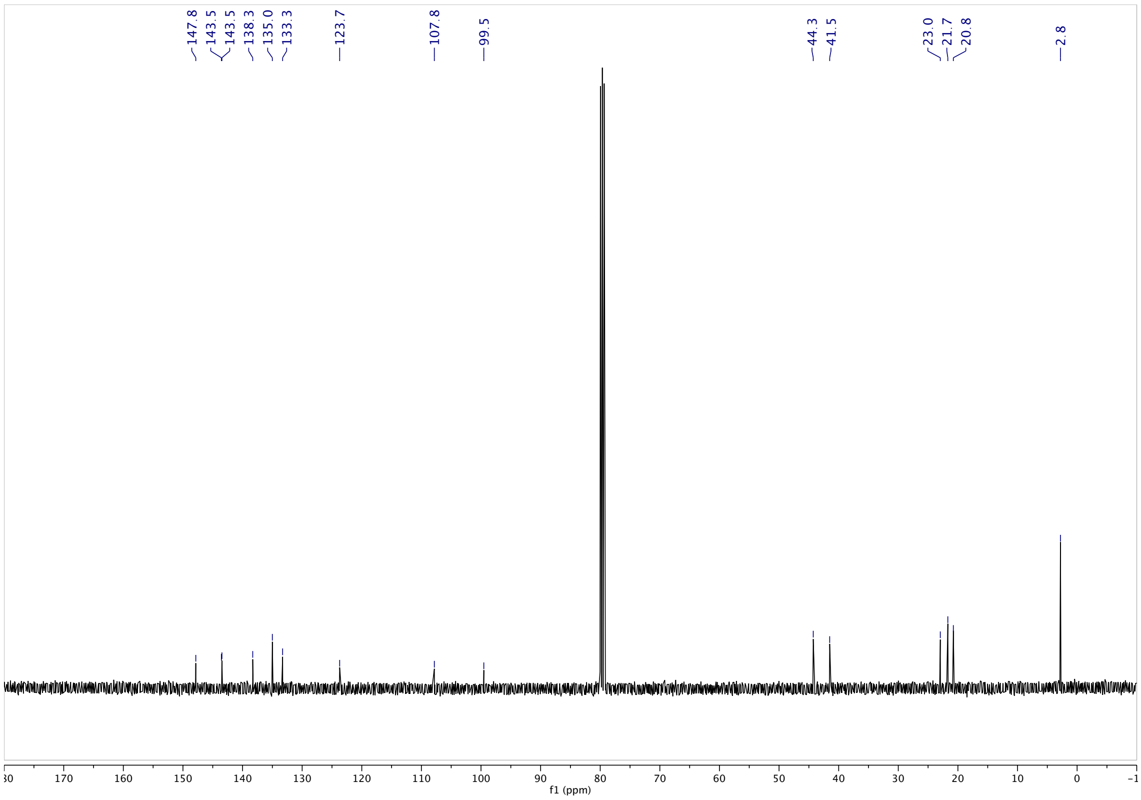

Figure S17. ^{13}C NMR (101 MHz, $CDCl_3$) of ***(P,P)-cis*-S3**.

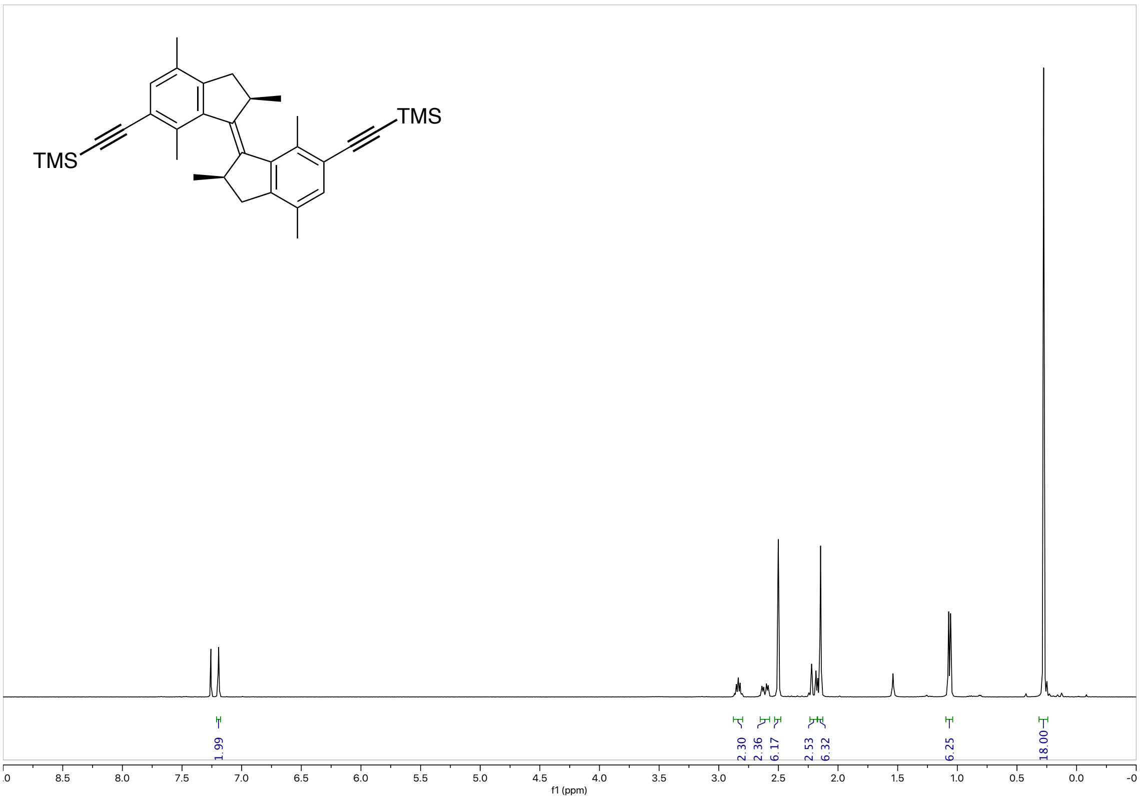

Figure S18. ^{1}H NMR (400 MHz, $CDCl_3$) of ***(P,P)-trans*-S3**.

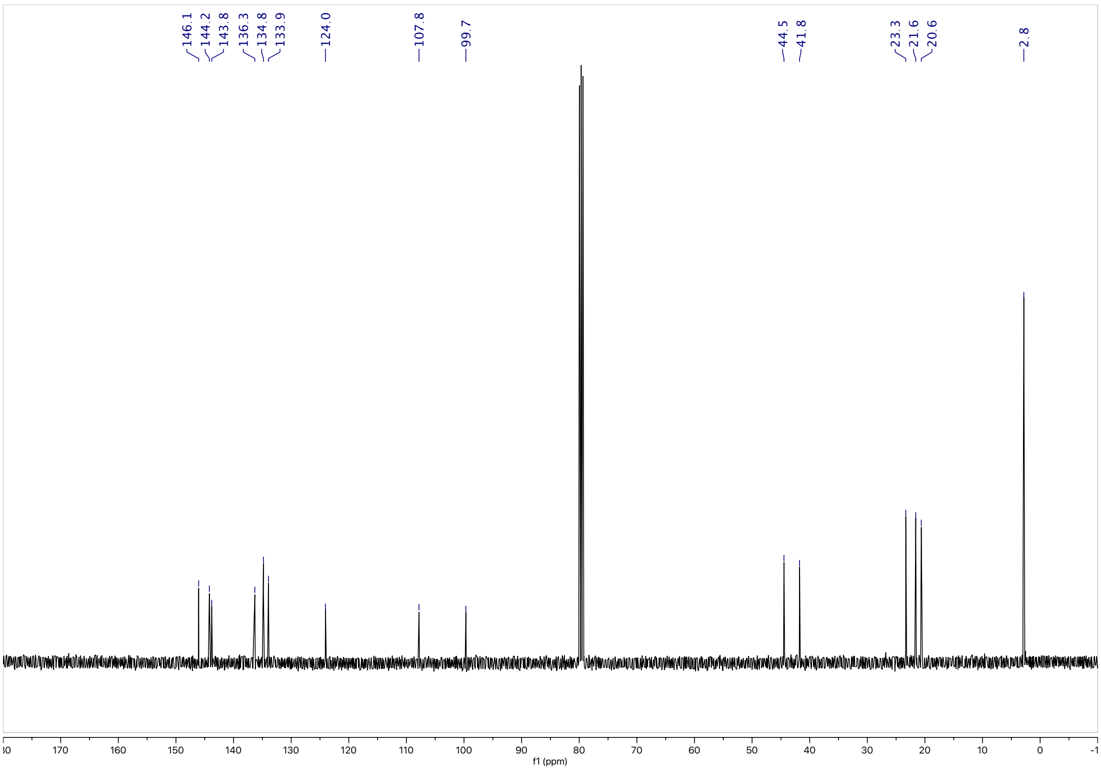

Figure S19. ^{13}C NMR (101 MHz, $CDCl_3$) of ***(P,P)-trans*-S3**.

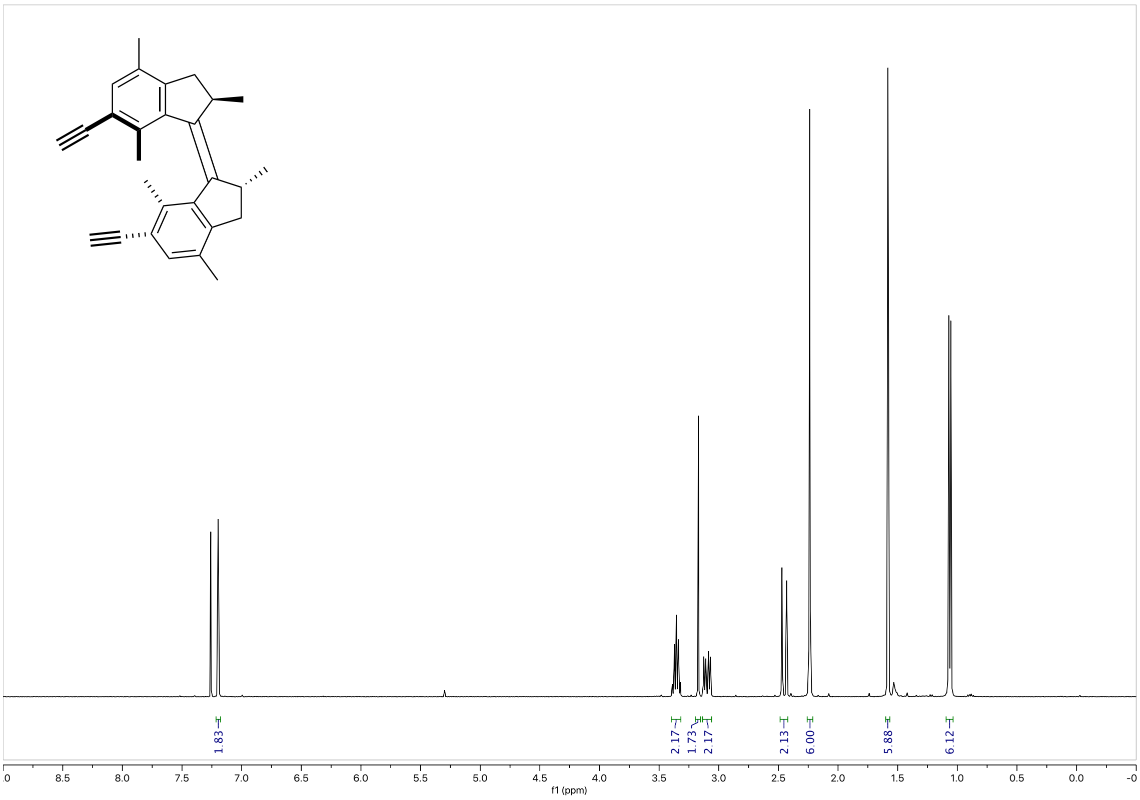

Figure S20. ^{1}H NMR (400 MHz, $CDCl_3$) of ***(P,P)*-*cis*-4**.

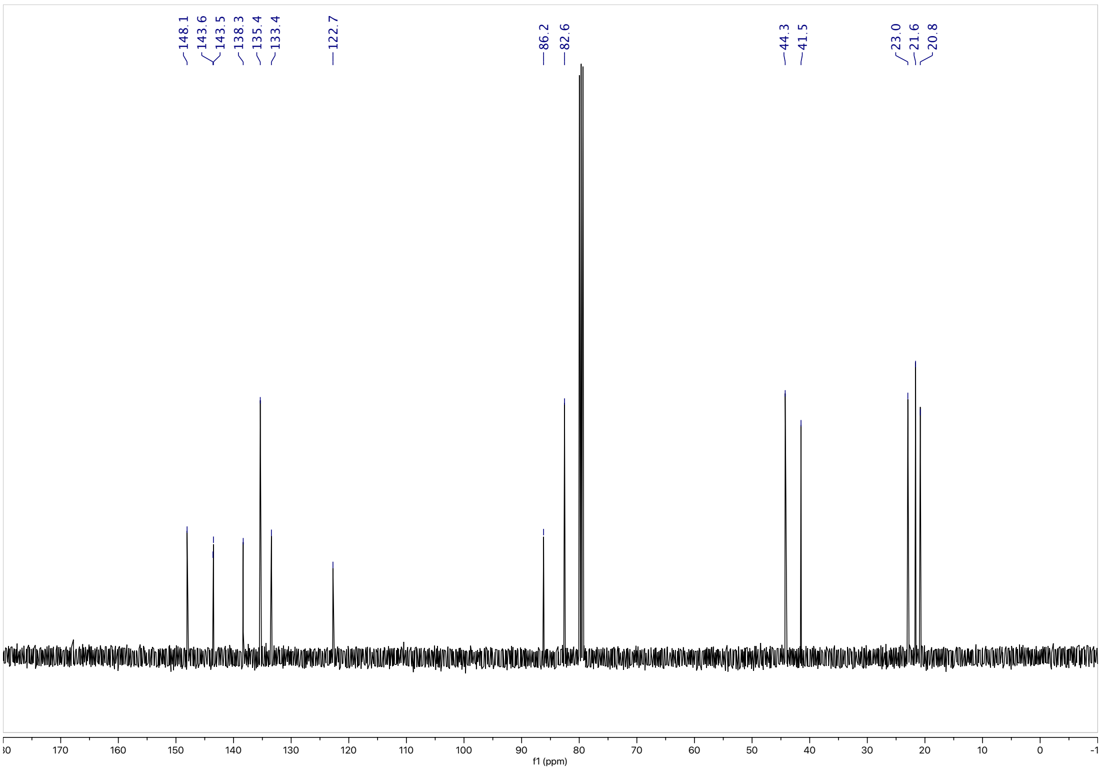

Figure S21. ^{13}C NMR (101 MHz, $CDCl_3$) of ***(P,P)*-*cis*-4**.

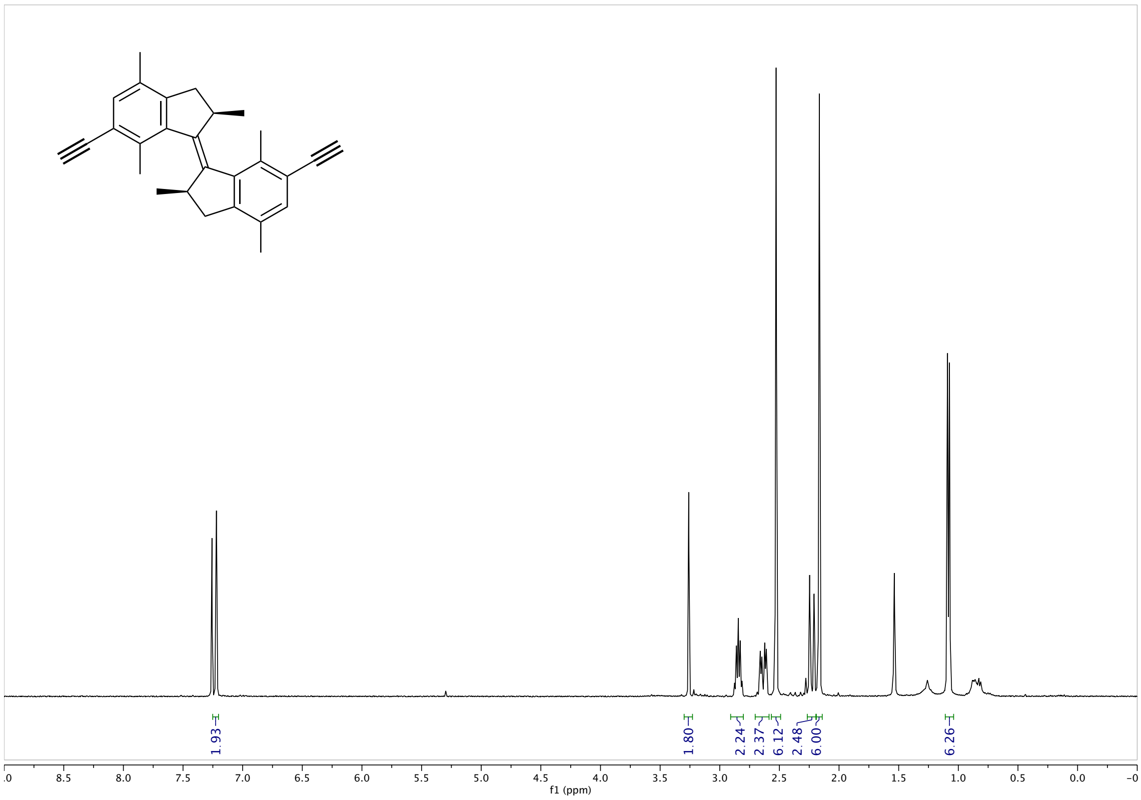

Figure S22. ^{1}H NMR (400 MHz, $CDCl_3$) of ***(P,P)-trans*-4**.

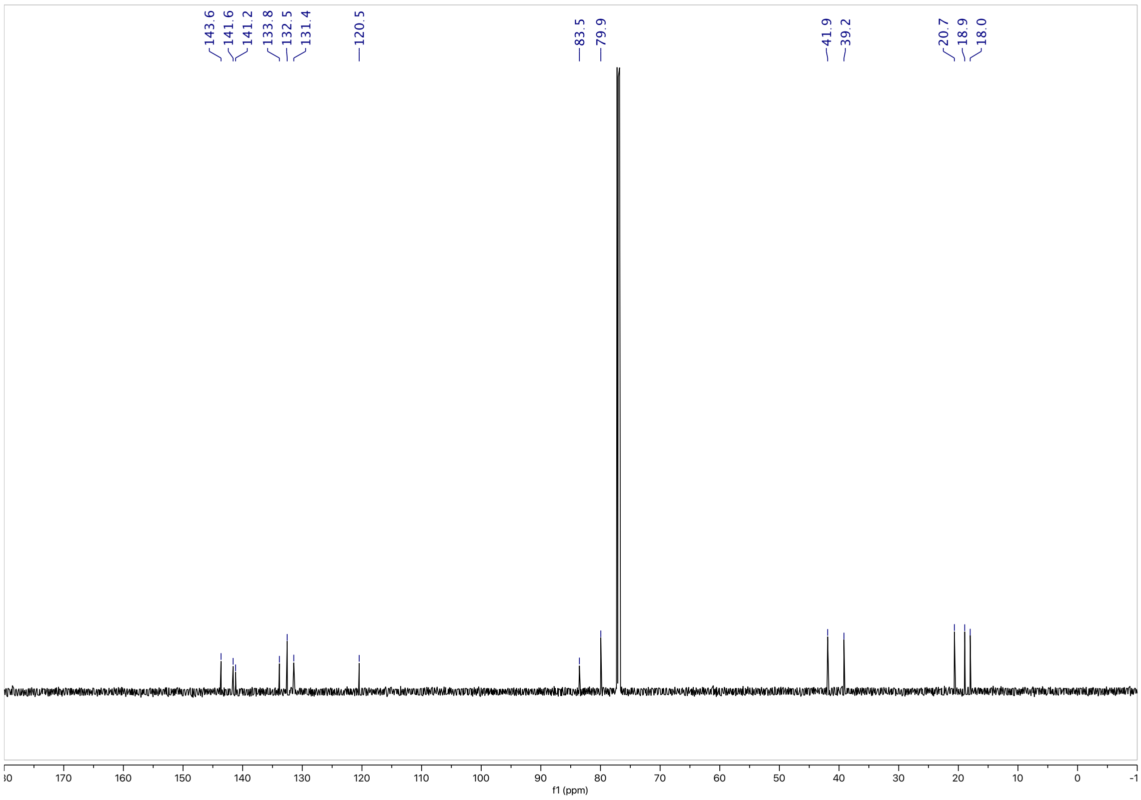

Figure S23. ^{13}C NMR (151 MHz, $CDCl_3$) of ***(P,P)-trans*-4**.

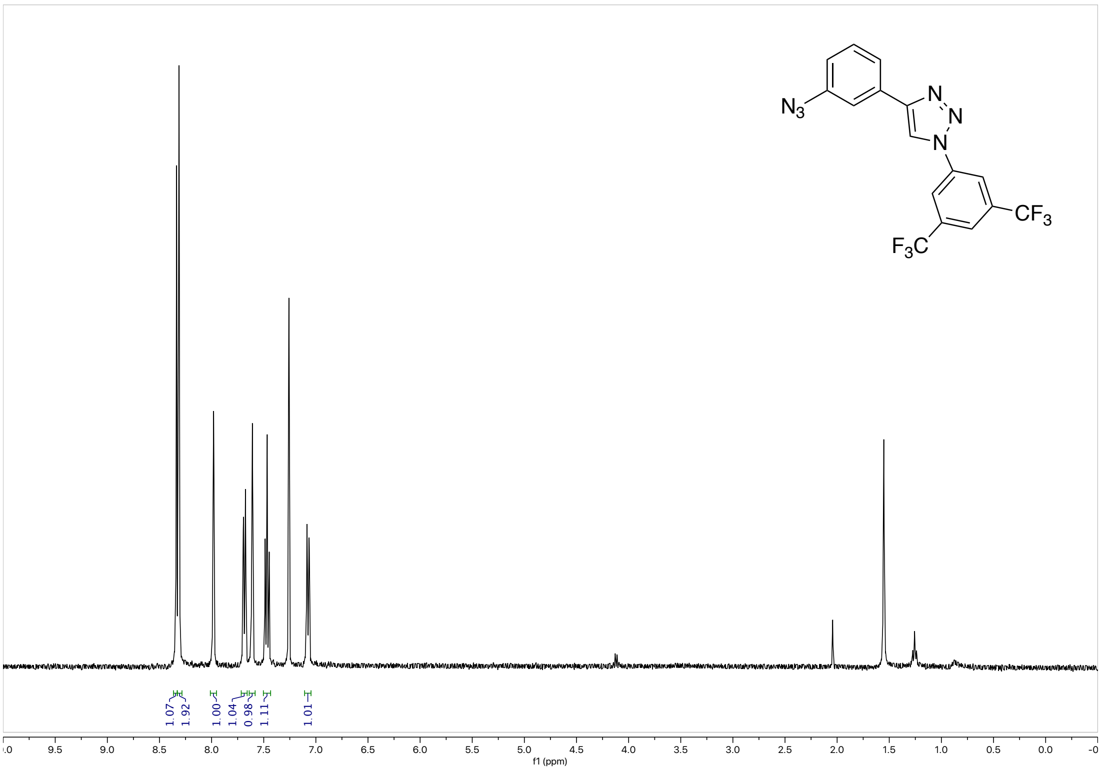

Figure S24. ^{1}H NMR (400 MHz, $CDCl_3$) of **5a**.

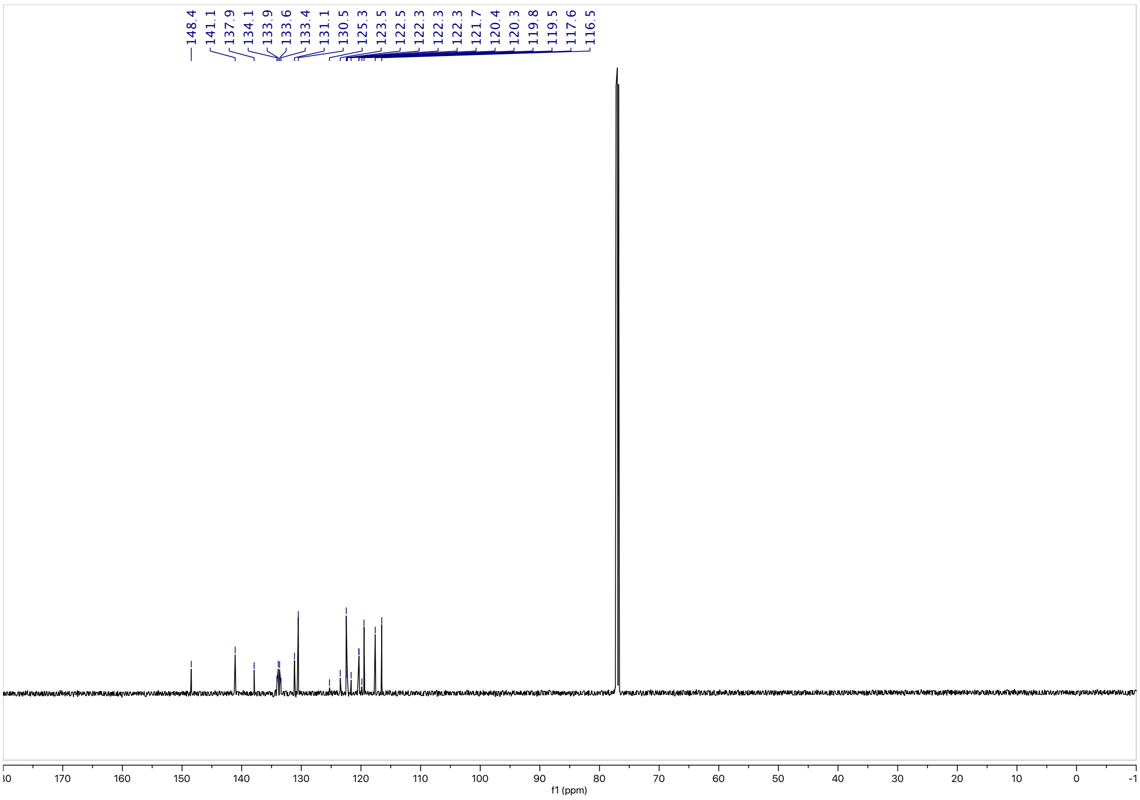

Figure S25. ^{13}C NMR (151 MHz, $CDCl_3$) of **5a**.

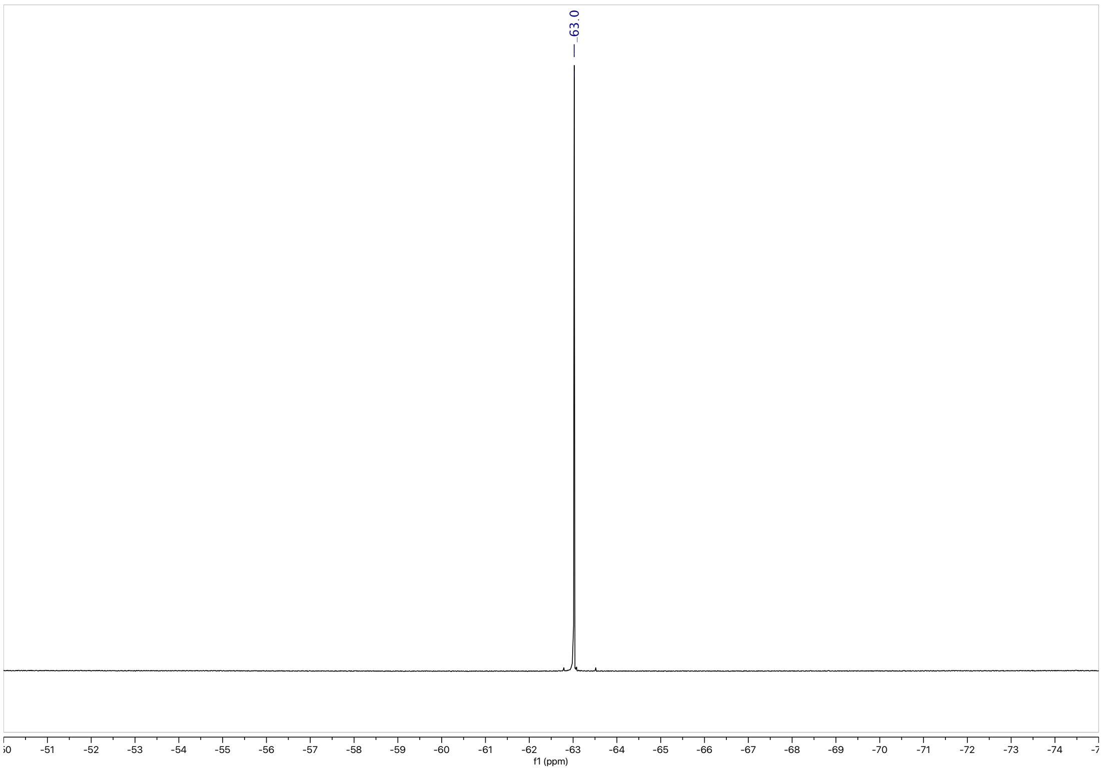

Figure S26. ^{19}F NMR (376 MHz, $CDCl_3$) of **5a**.

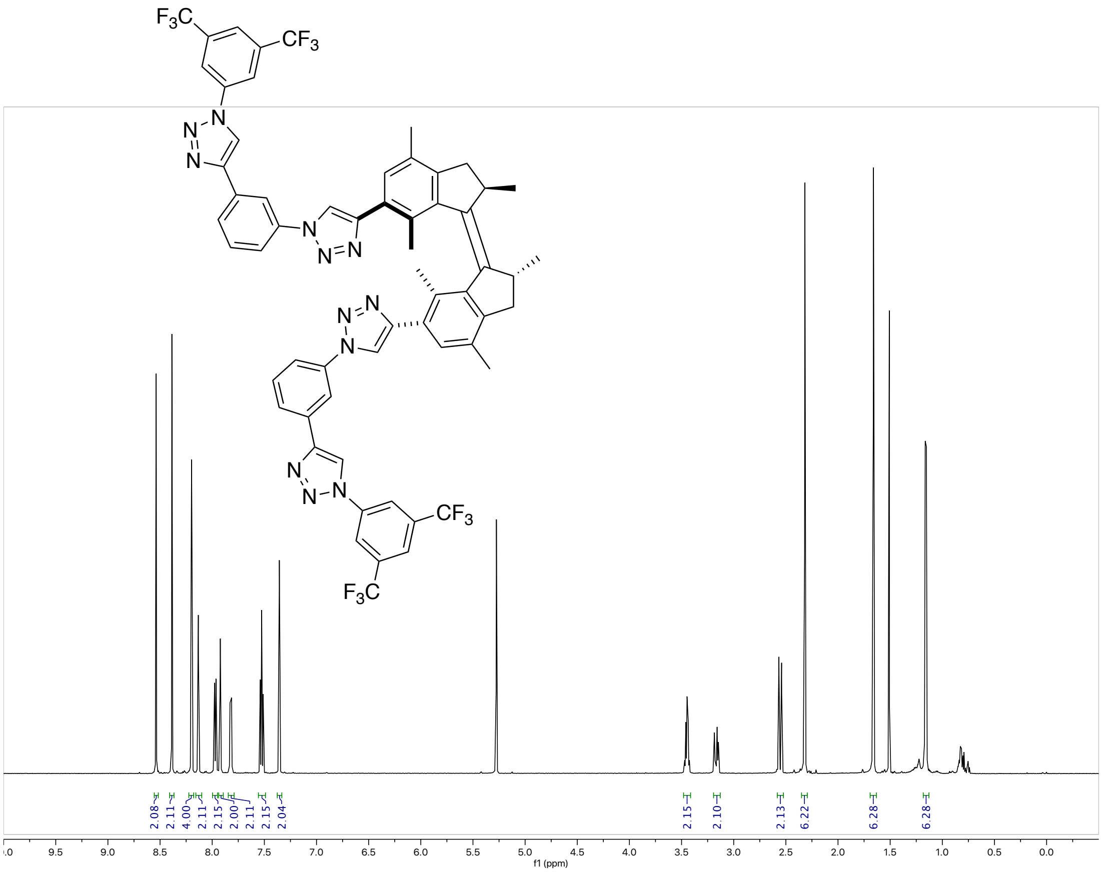

Figure S27. ^{1}H NMR (600 MHz, CD_2Cl_2) of ***(P,P)*-*cis*-1a**.

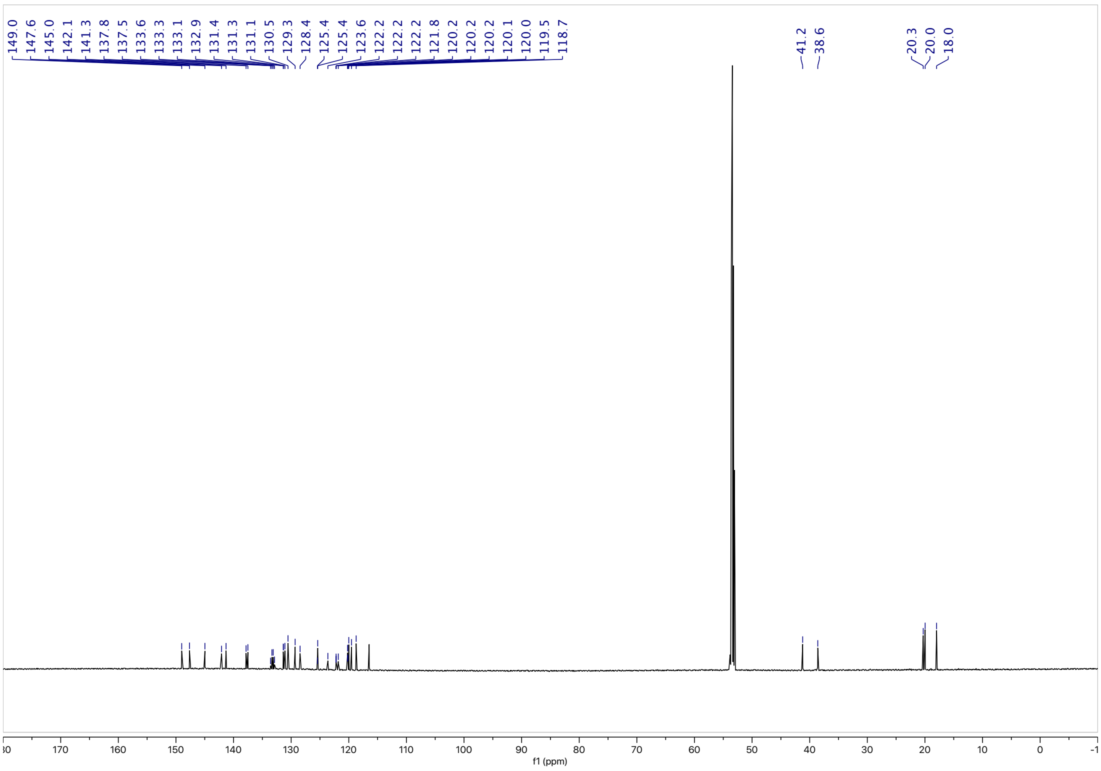

Figure S28. ^{13}C NMR (151 MHz, CD_2Cl_2) of ***(P,P)*-*cis*-1a**.

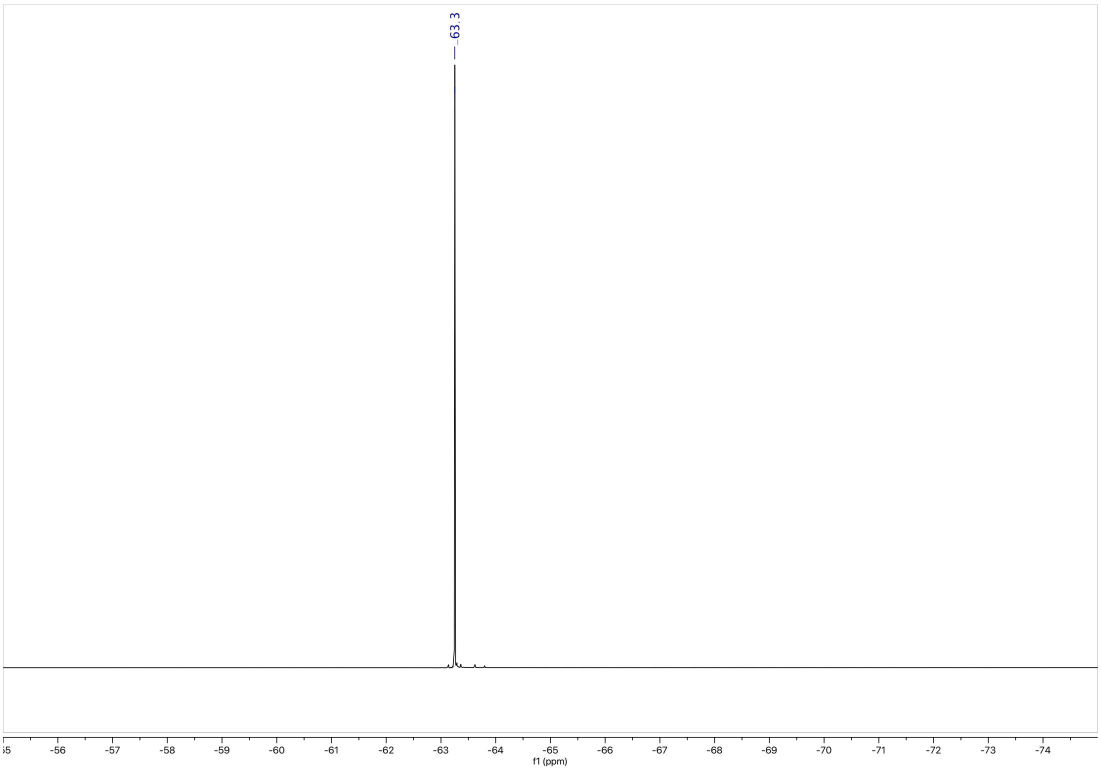

Figure S29. ^{19}F NMR (565 MHz, CD_2Cl_2) of ***(P,P)*-*cis*-1a**.

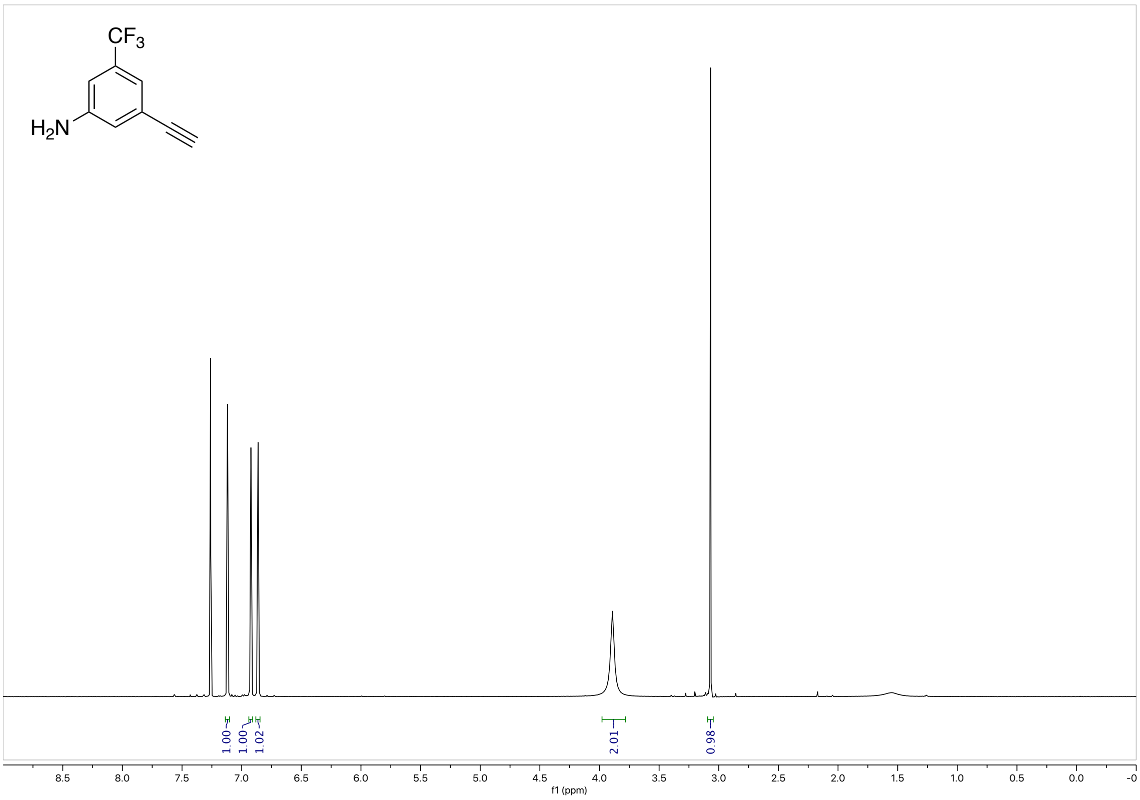

Figure S30. ^{1}H NMR (600 MHz, $CDCl_3$) of **S4**.

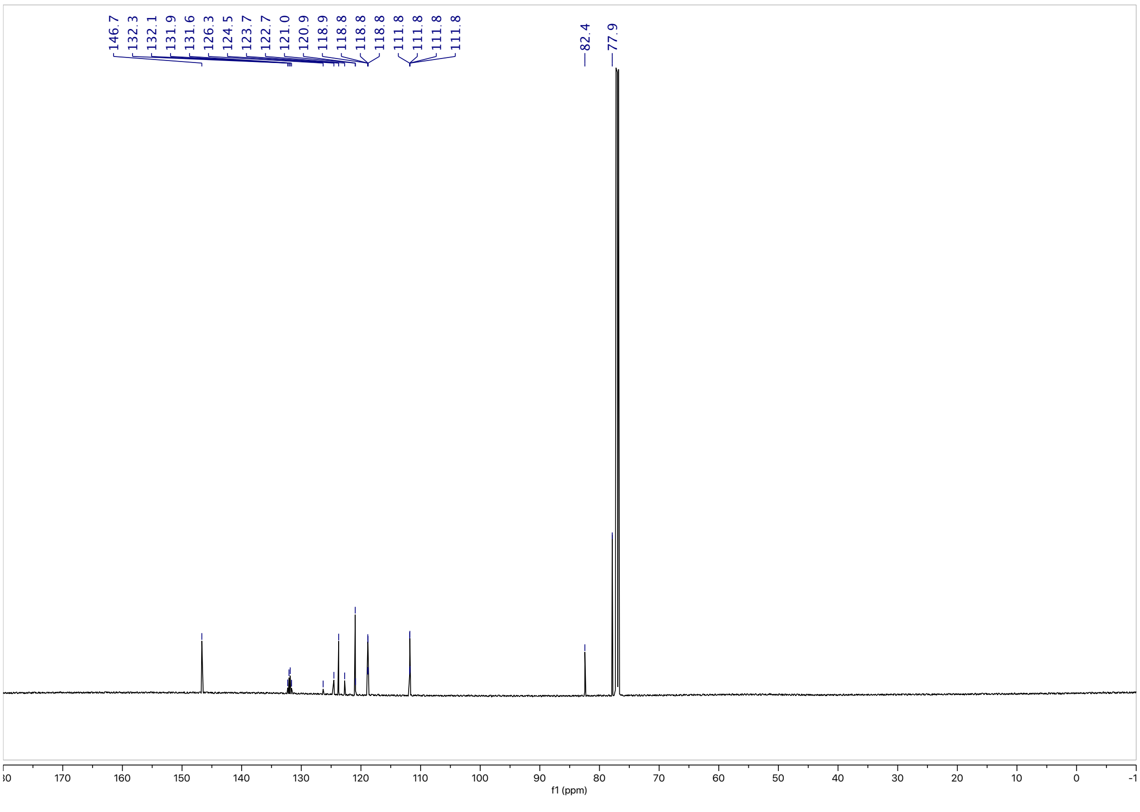

Figure S31. ^{13}C NMR (151 MHz, $CDCl_3$) of **S4**.

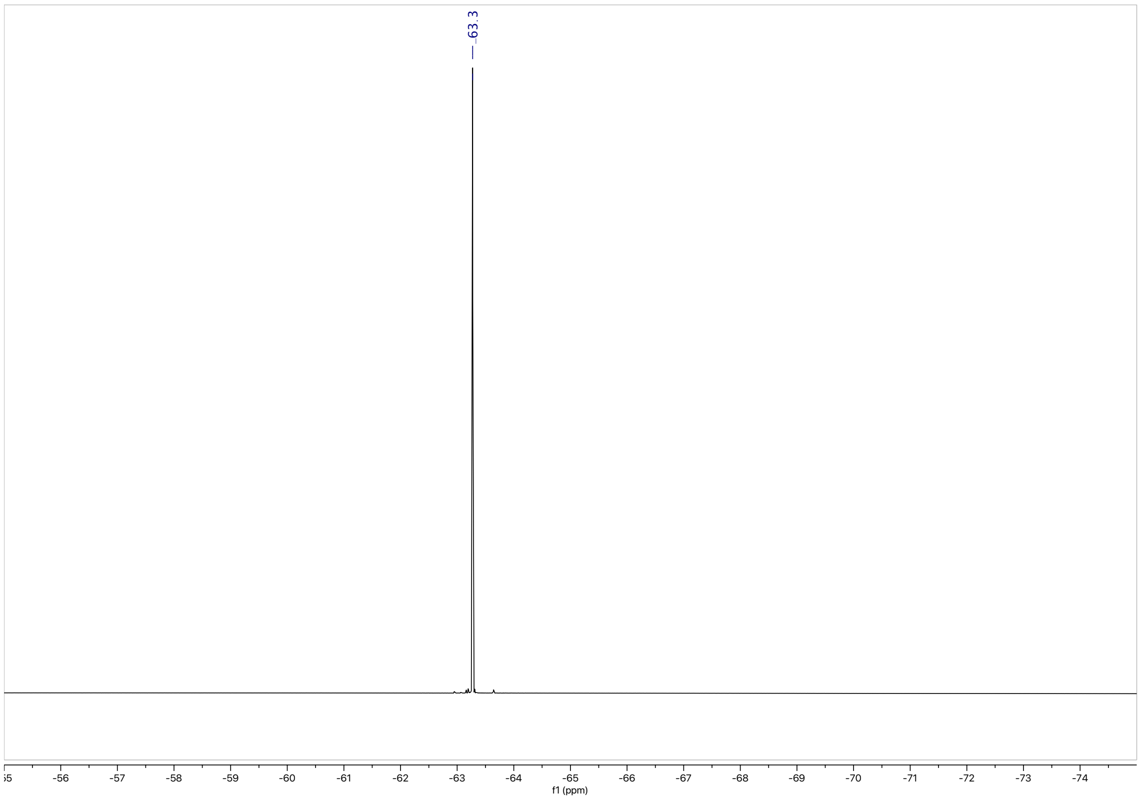

Figure S32. ^{19}F NMR (565 MHz, $CDCl_3$) of **S4**.

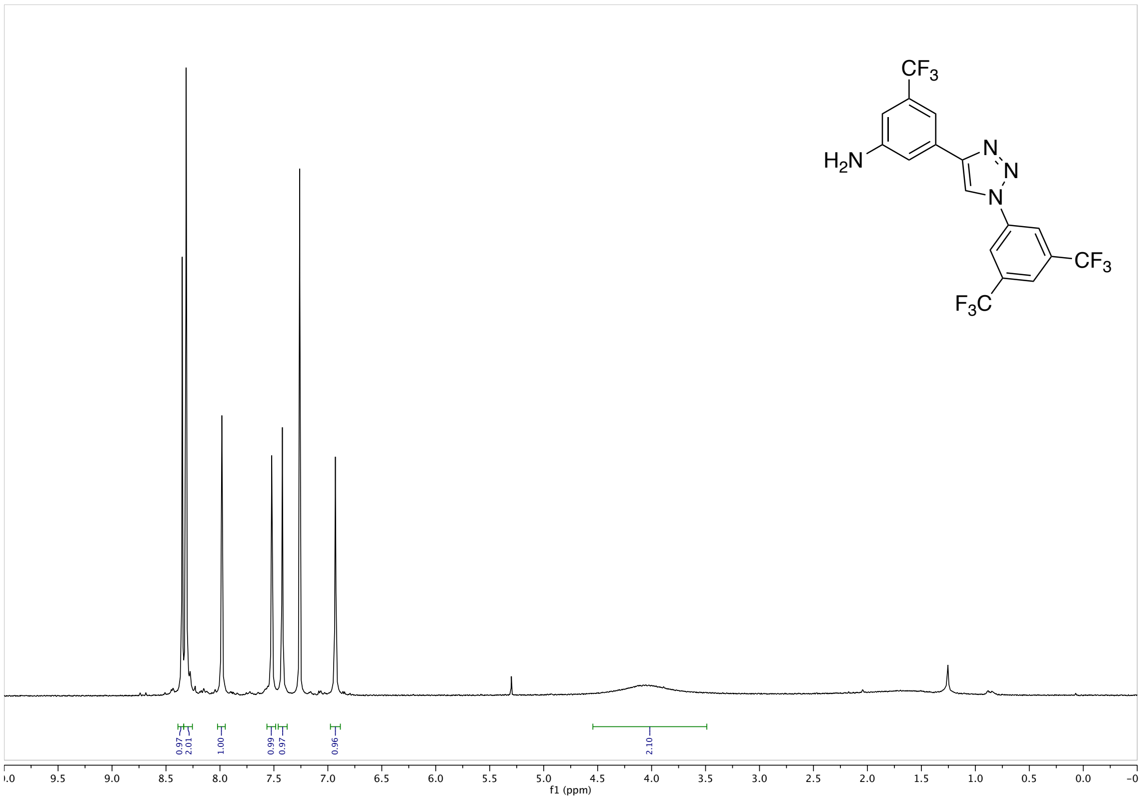

Figure S33. ^{1}H NMR (600 MHz, $CDCl_3$) of **S5**.

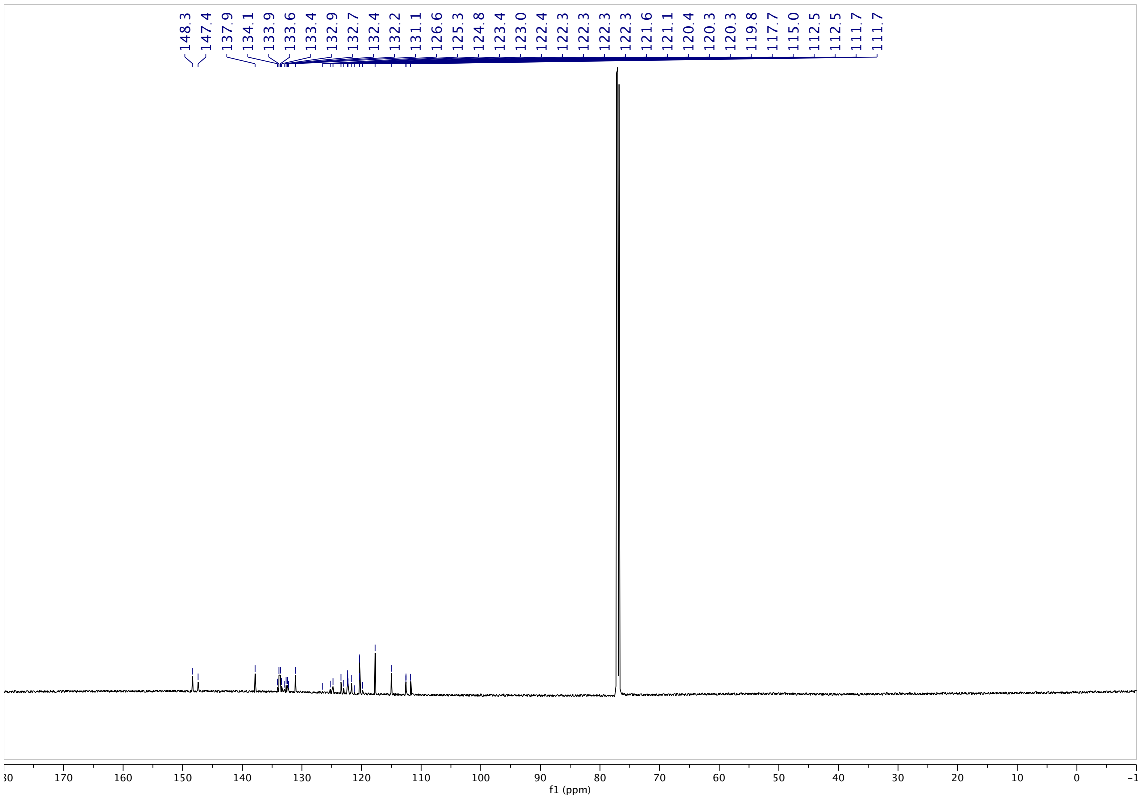

Figure S34. ^{13}C NMR (151 MHz, $CDCl_3$) of **S5**.

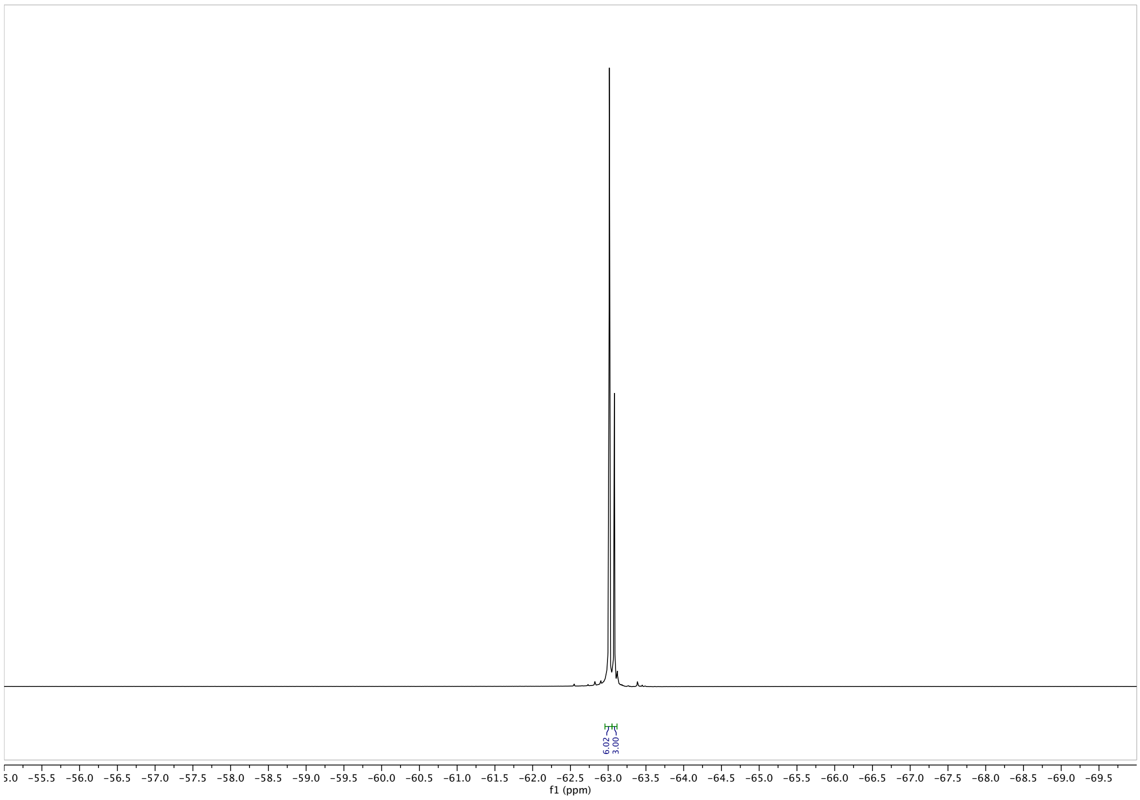

Figure S35. ^{19}F NMR (565 MHz, $CDCl_3$) of **S5**.

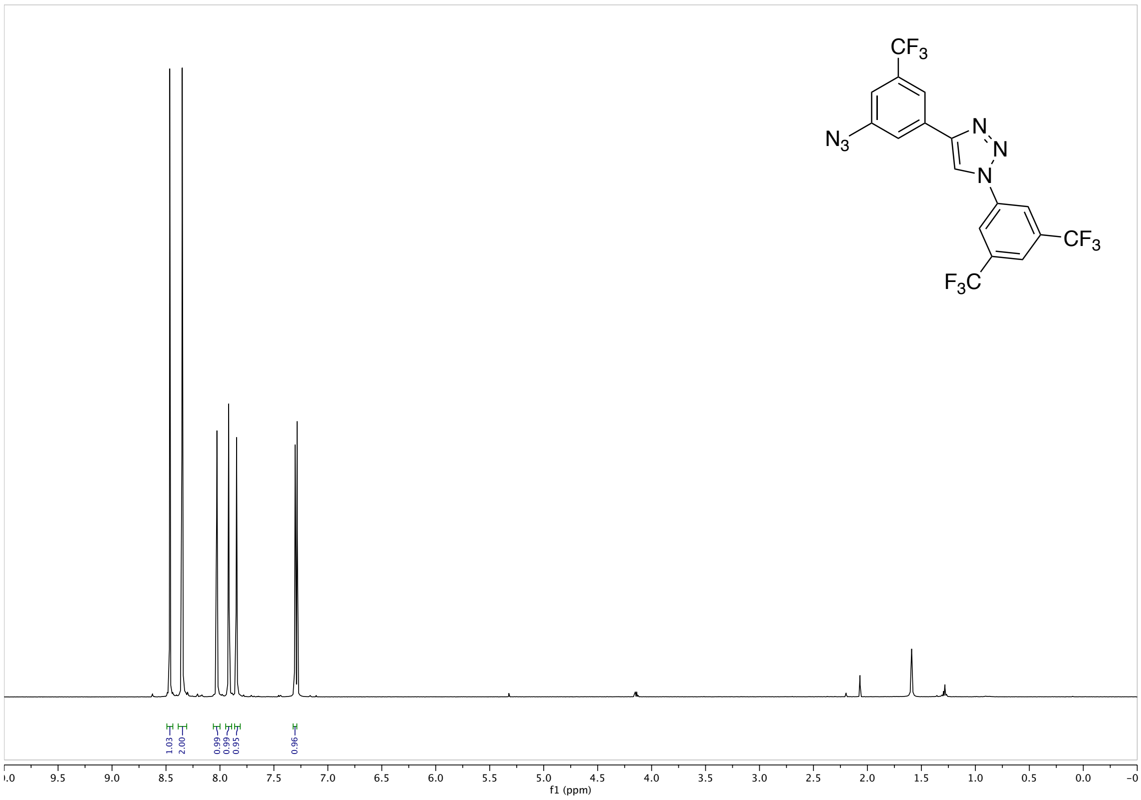

Figure S36. ^{1}H NMR (600 MHz, $CDCl_3$) of **5b**.

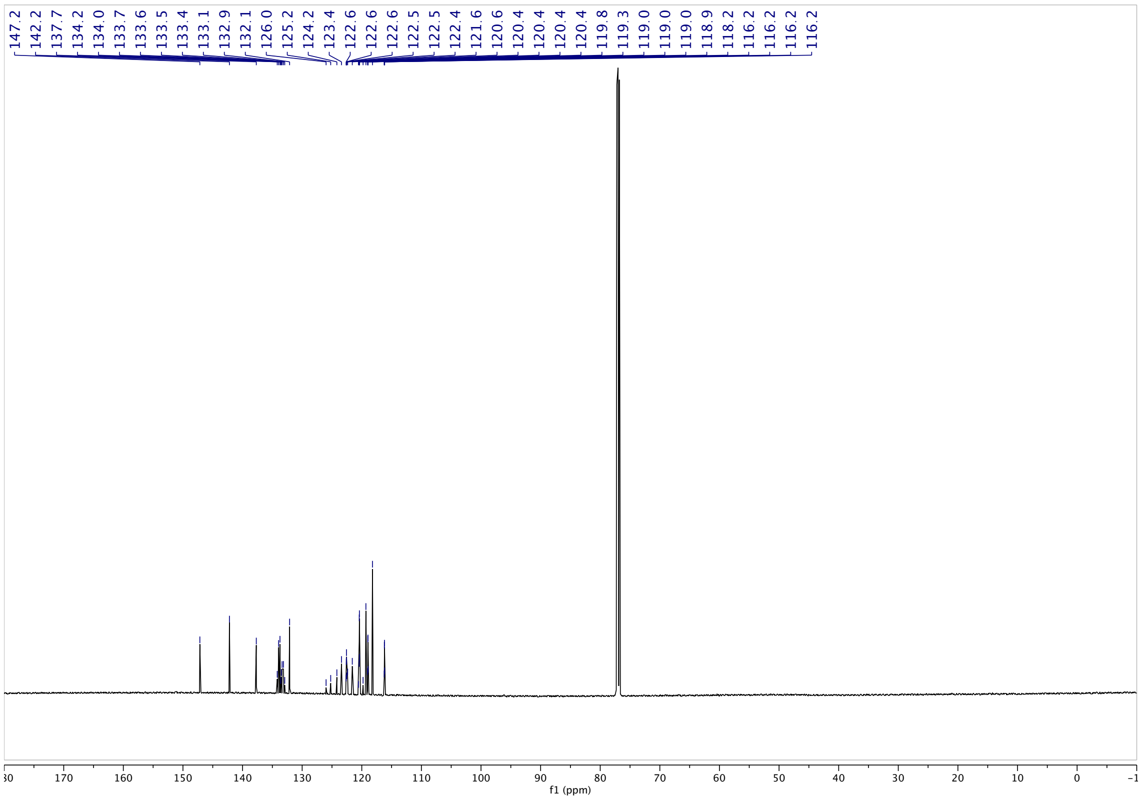

Figure S37. ^{13}C NMR (151 MHz, $CDCl_3$) of **5b**.

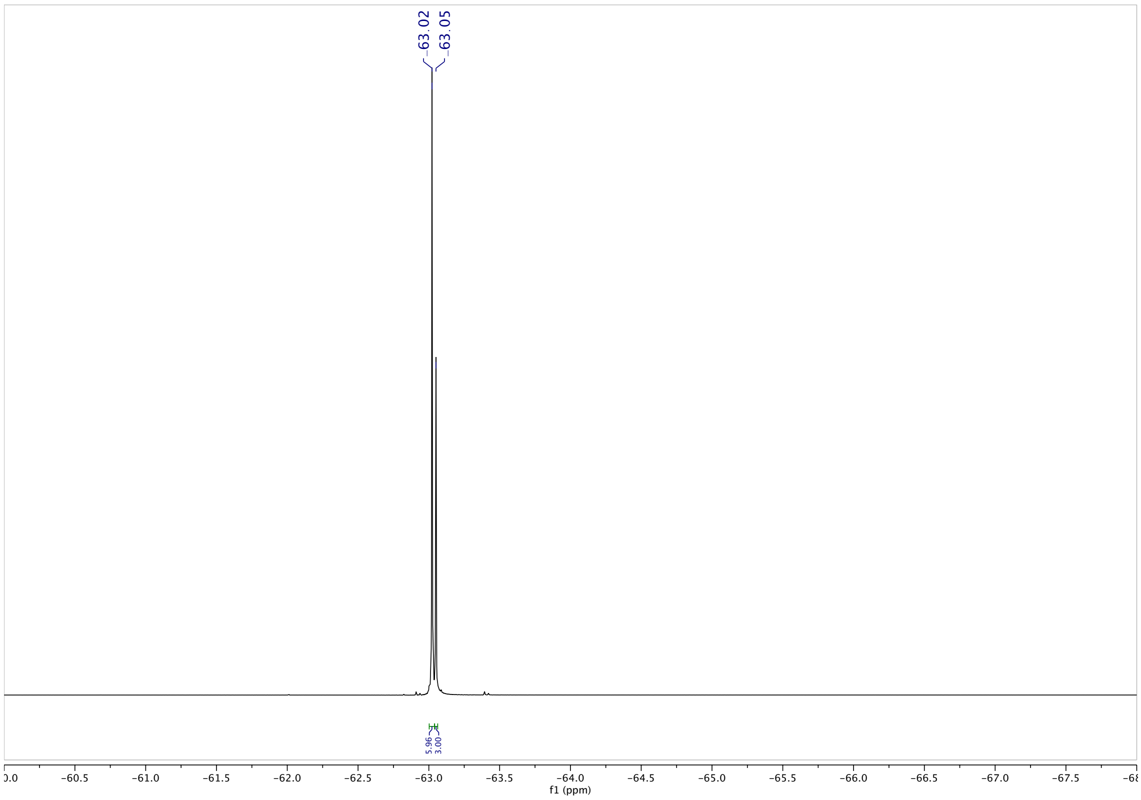

Figure S38. ^{19}F NMR (565 MHz, $CDCl_3$) of **5b**.

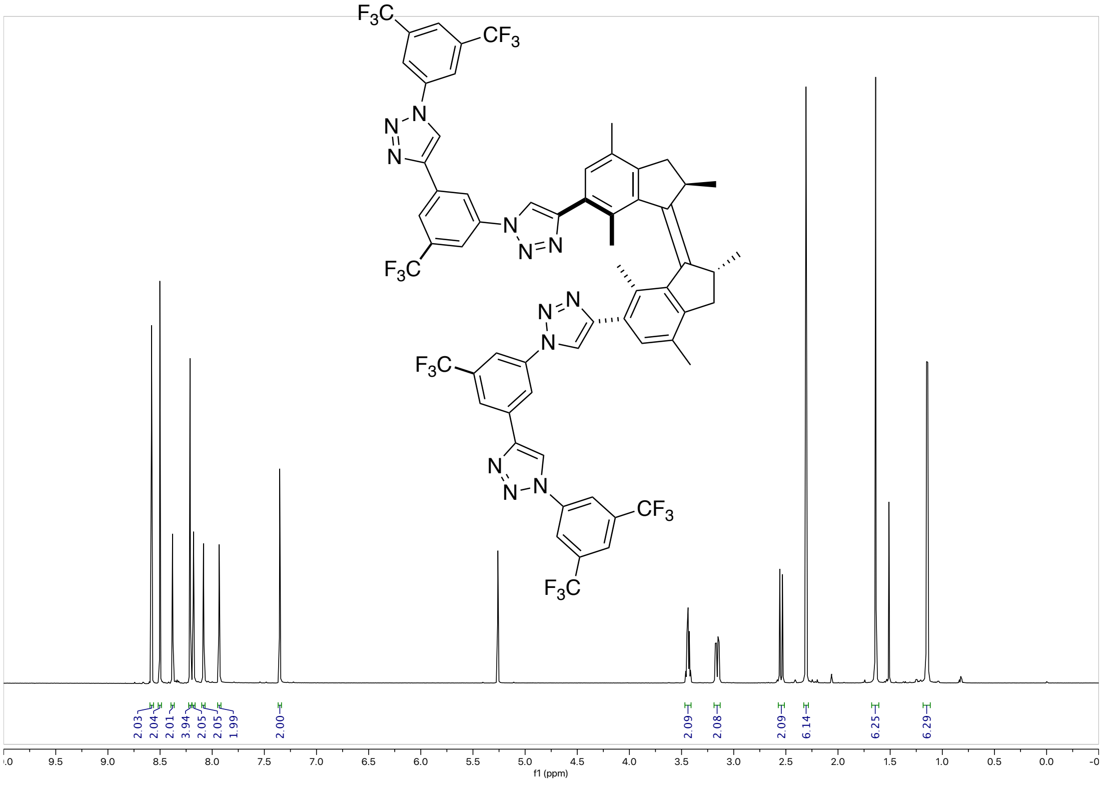

Figure S39. ^{1}H NMR (600 MHz, CD_2Cl_2) of **(*P*,*P*)-*cis*-1b.**

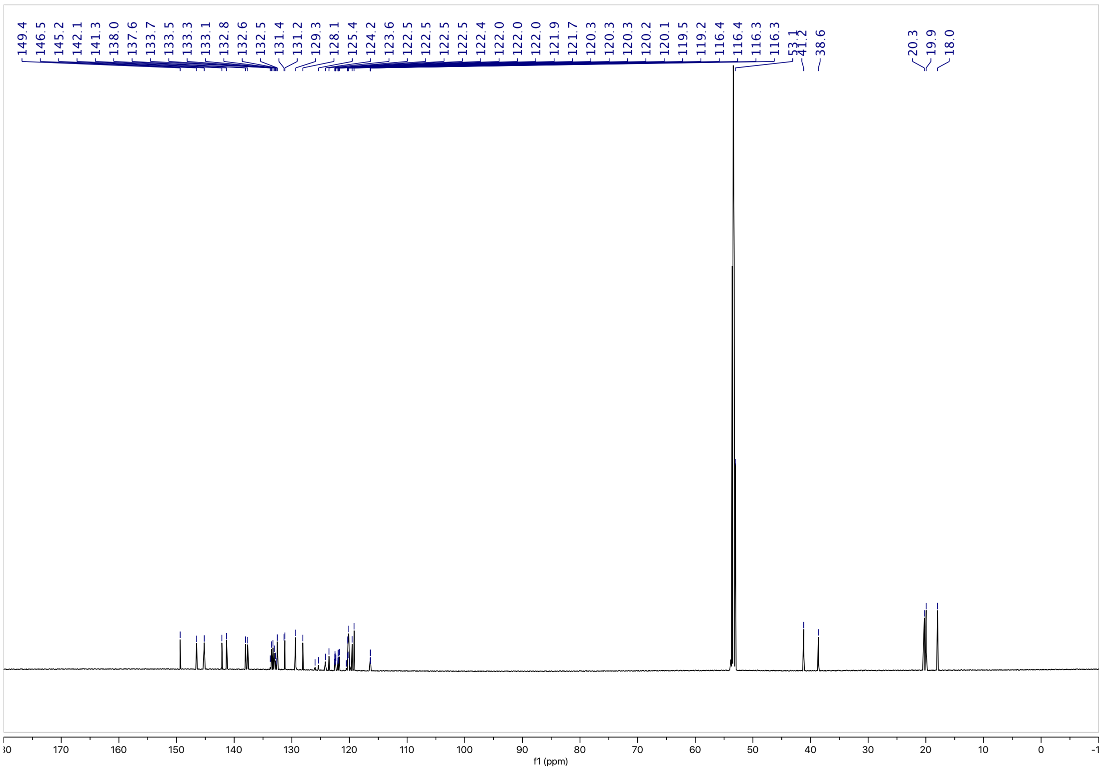

Figure S40. ^{13}C NMR (151 MHz, CD_2Cl_2) of ***(P,P)*-*cis*-1b**.

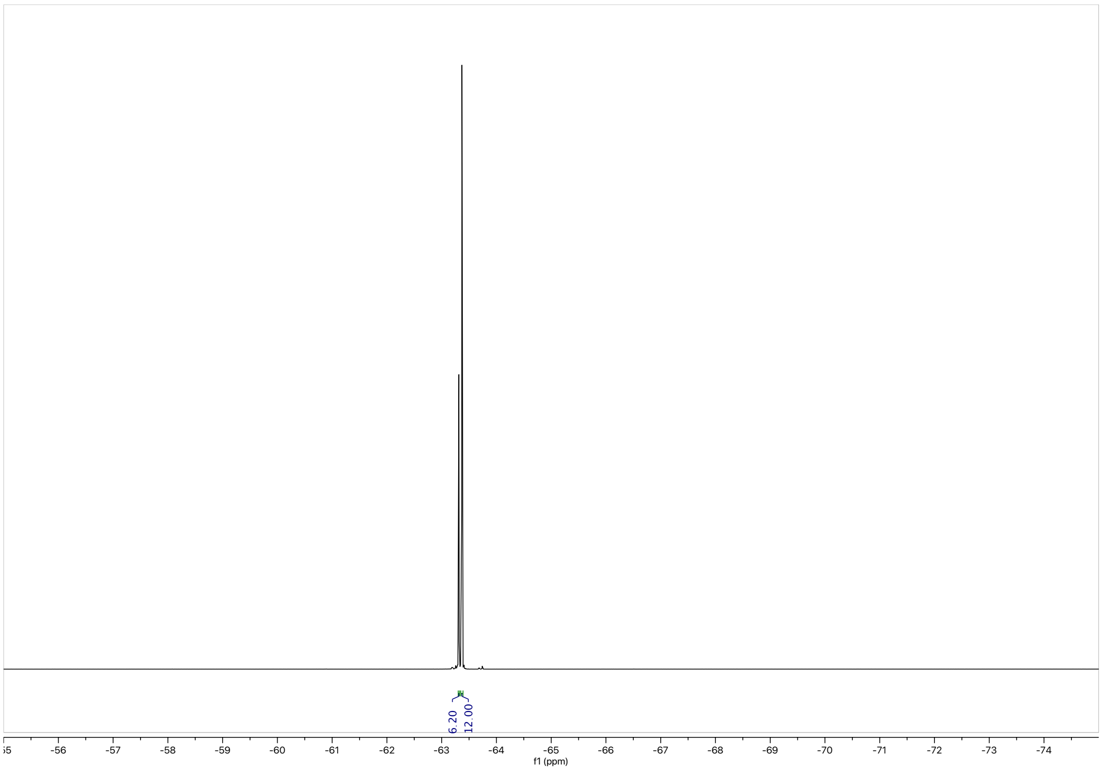

Figure S41. ^{19}F NMR (565 MHz, CD_2Cl_2) of ***(P,P)*-*cis*-1b**.

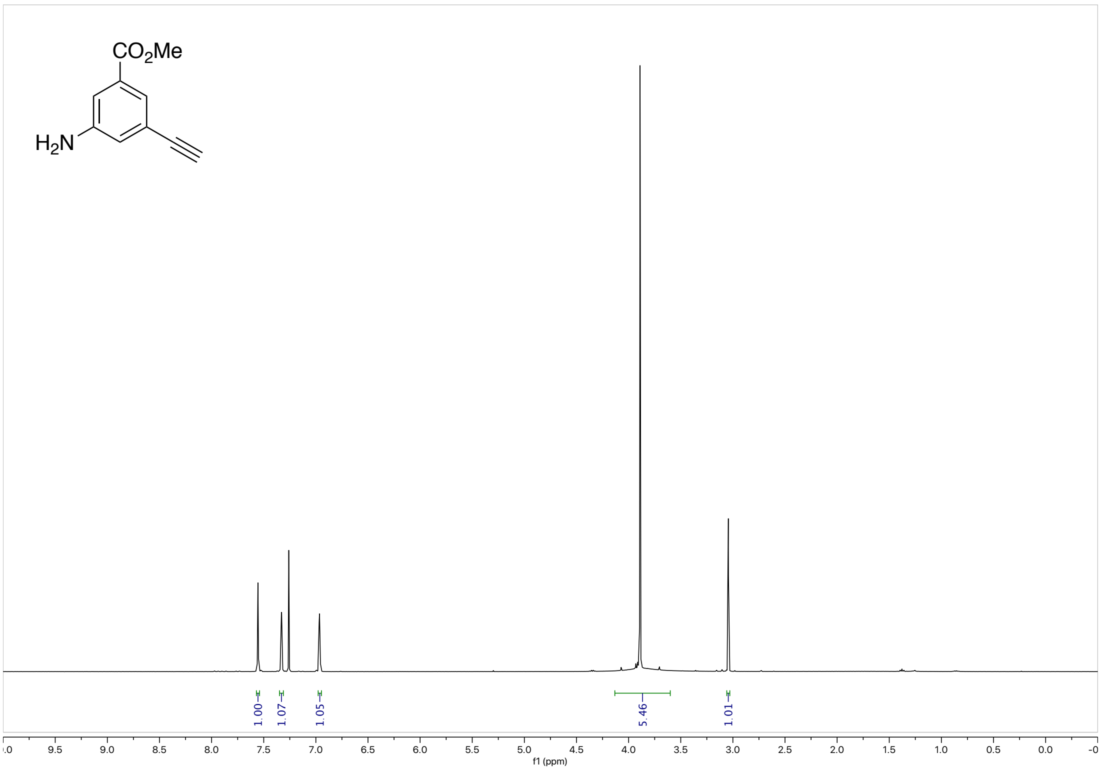

Figure S42. ^{1}H NMR (400 MHz, $CDCl_3$) of **S6.**

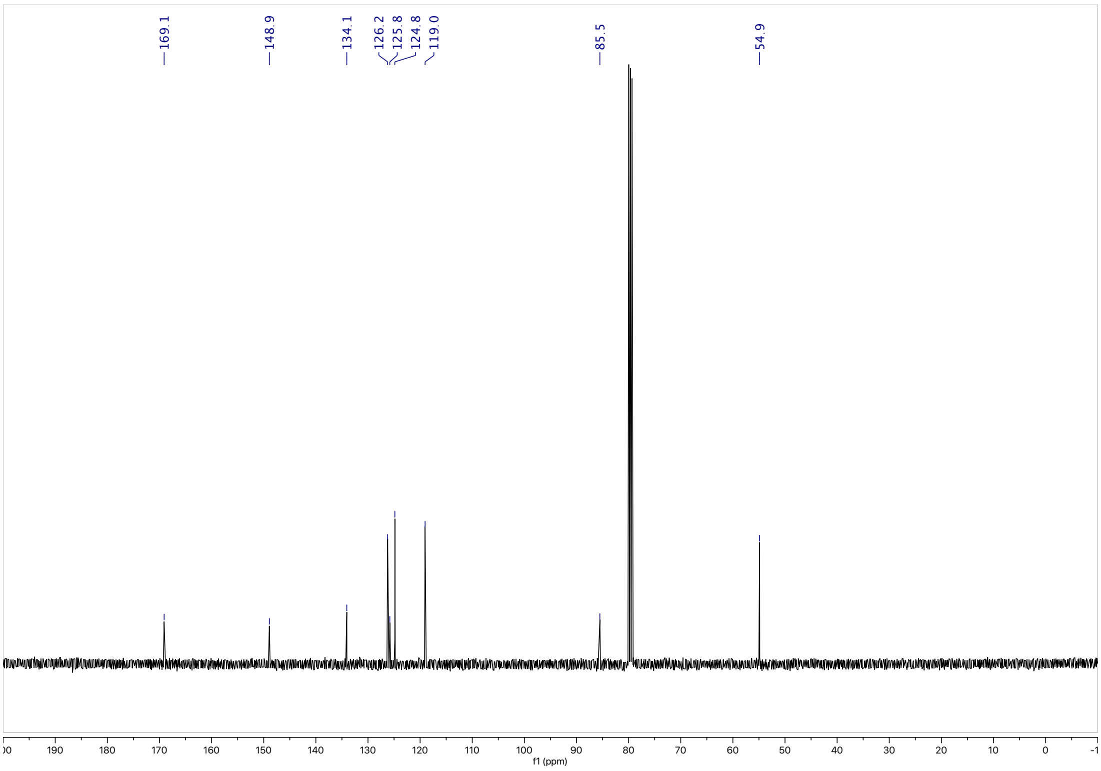

Figure S43. ^{13}C NMR (101 MHz, $CDCl_3$) of **S6**.

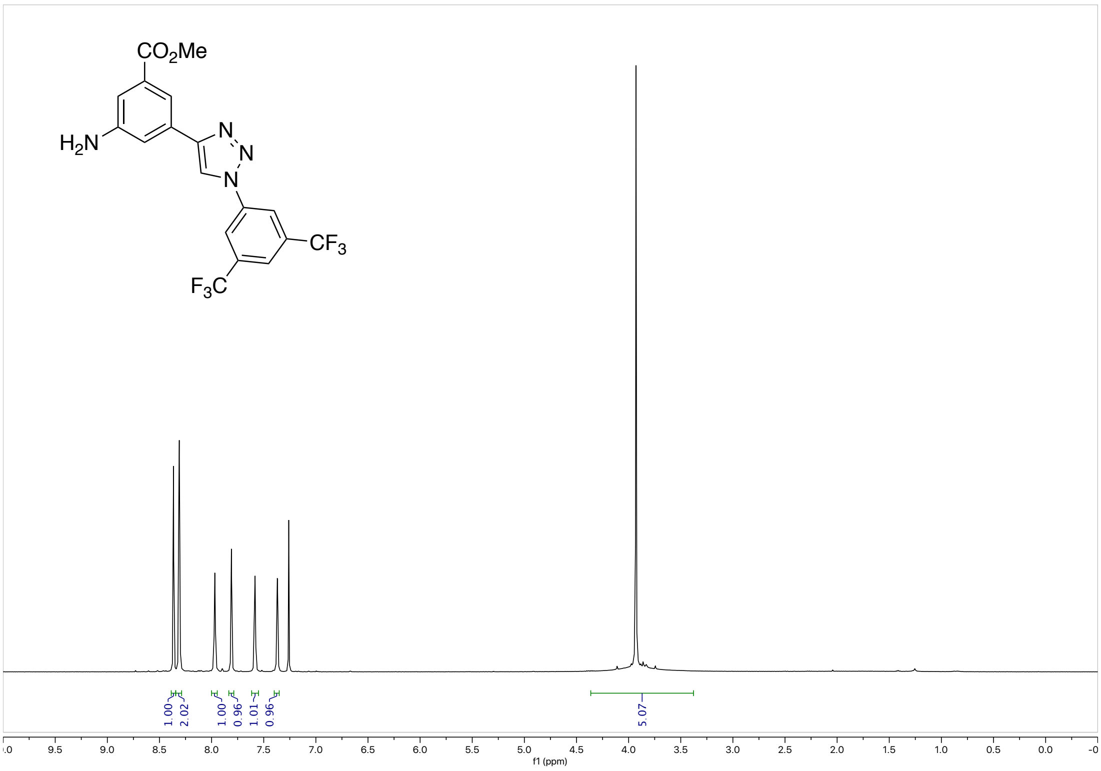

Figure S44. ^{1}H NMR (400 MHz, $CDCl_3$) of **S7.**

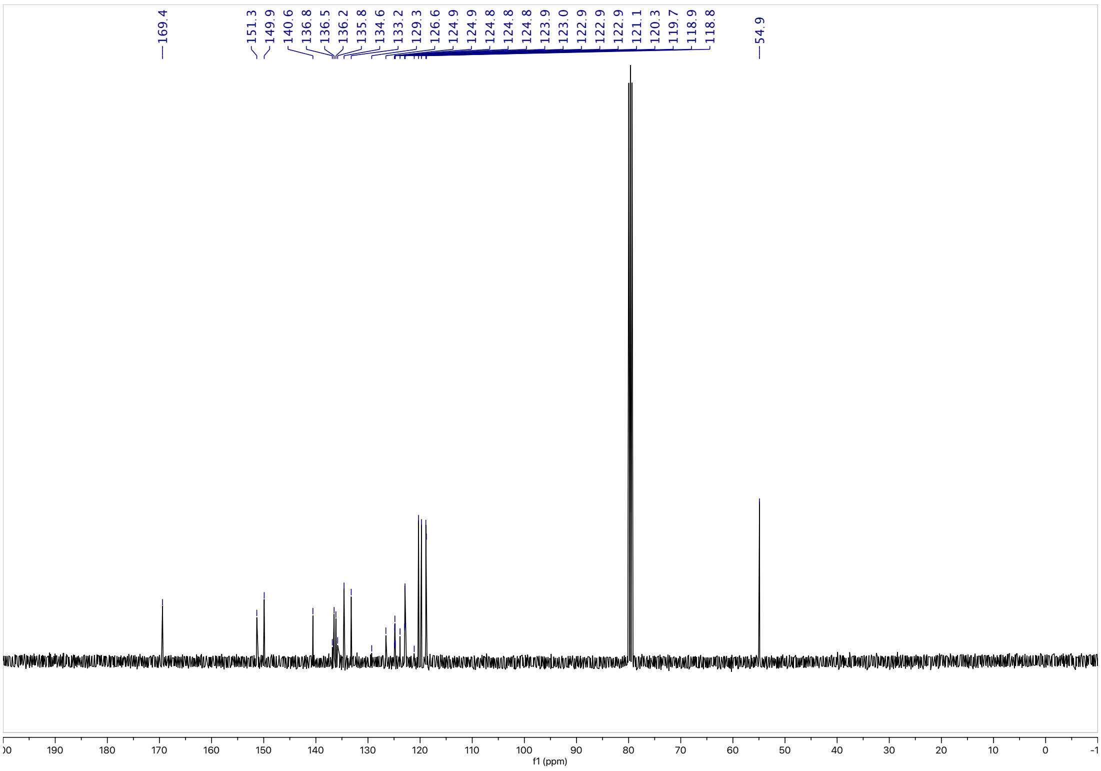

Figure S45. ^{13}C NMR (101 MHz, $CDCl_3$) of **S7**.

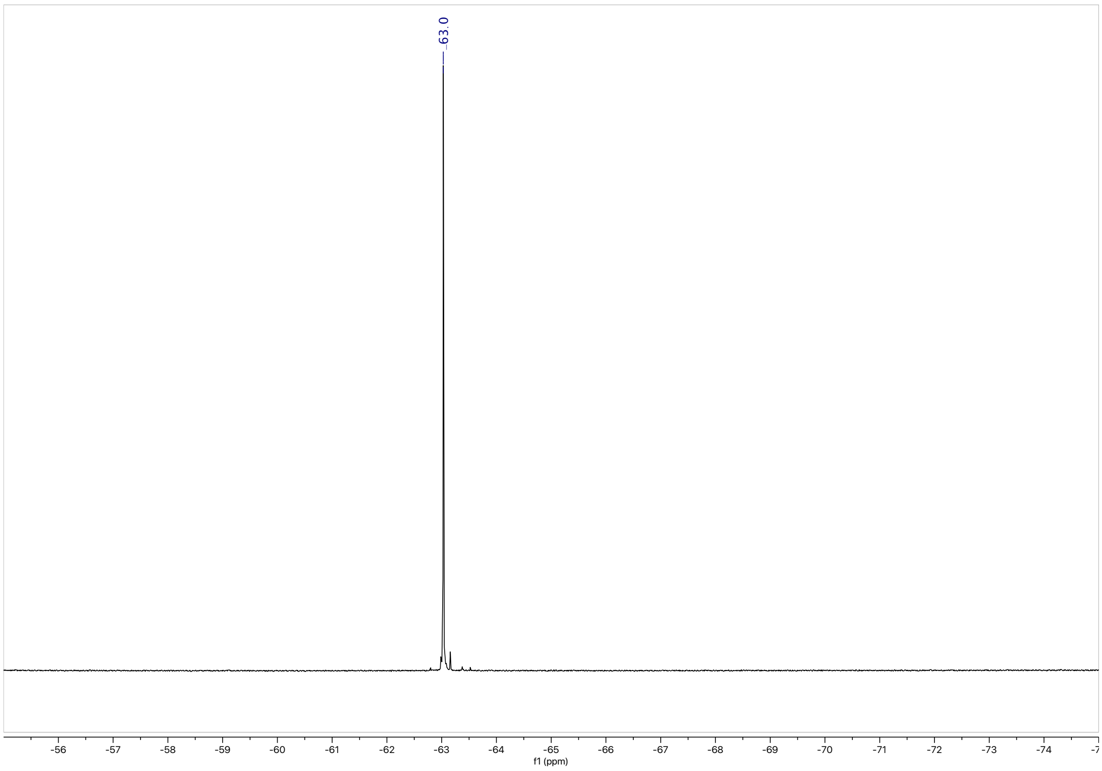

Figure S46. ^{19}F NMR (376 MHz, $CDCl_3$) of **S7**.

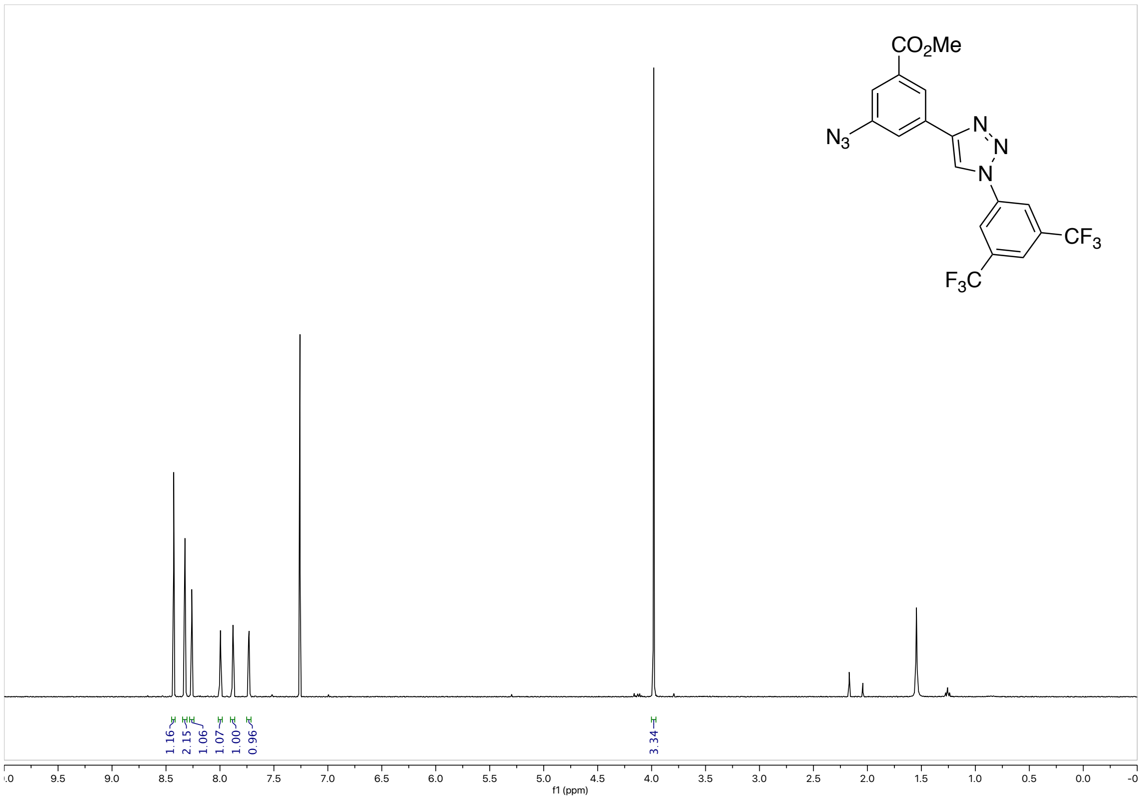

Figure S47. ^{1}H NMR (400 MHz, $CDCl_3$) of **5c.**

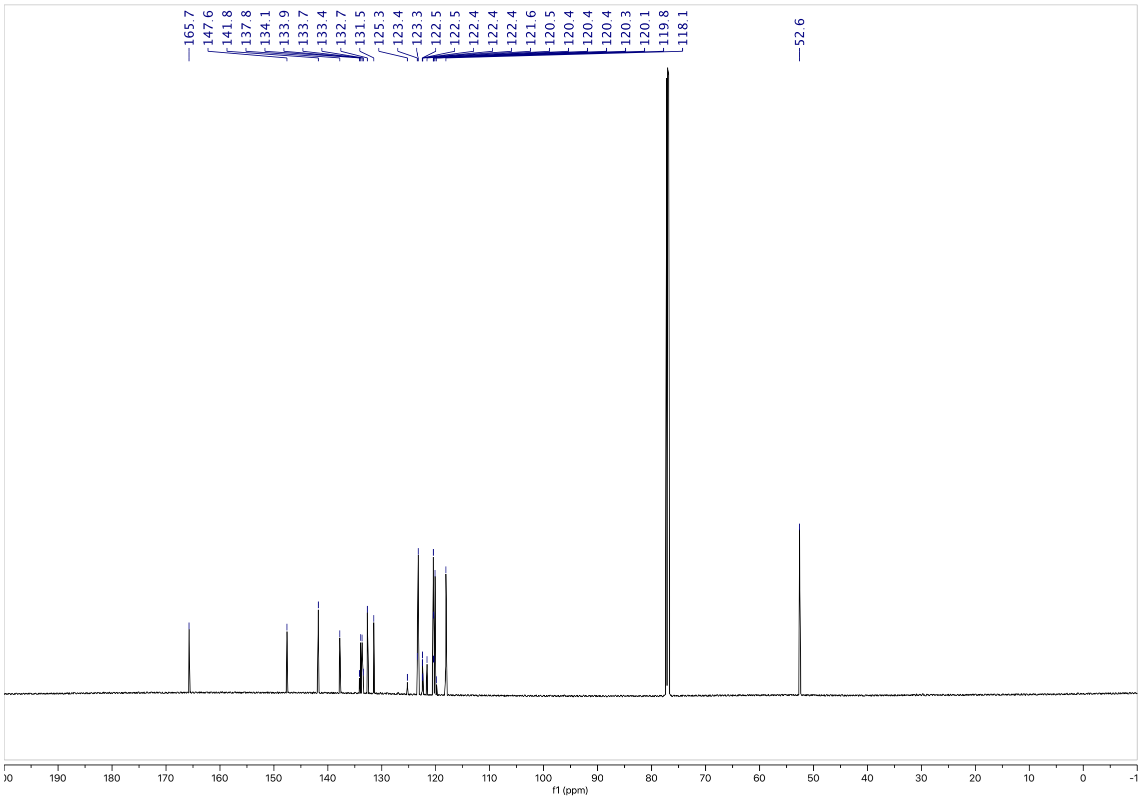

Figure S48. ^{13}C NMR (151 MHz, $CDCl_3$) of **5c**.

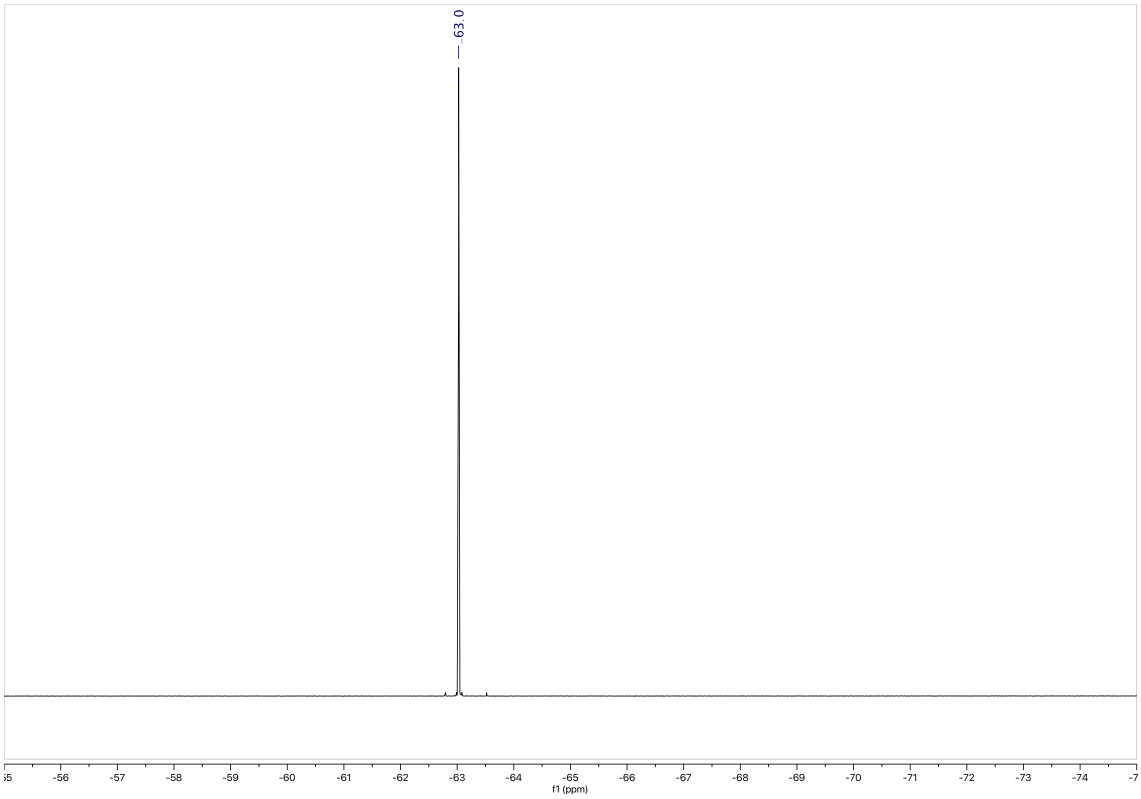

Figure S49. ^{19}F NMR (376 MHz, $CDCl_3$) of **5c**.

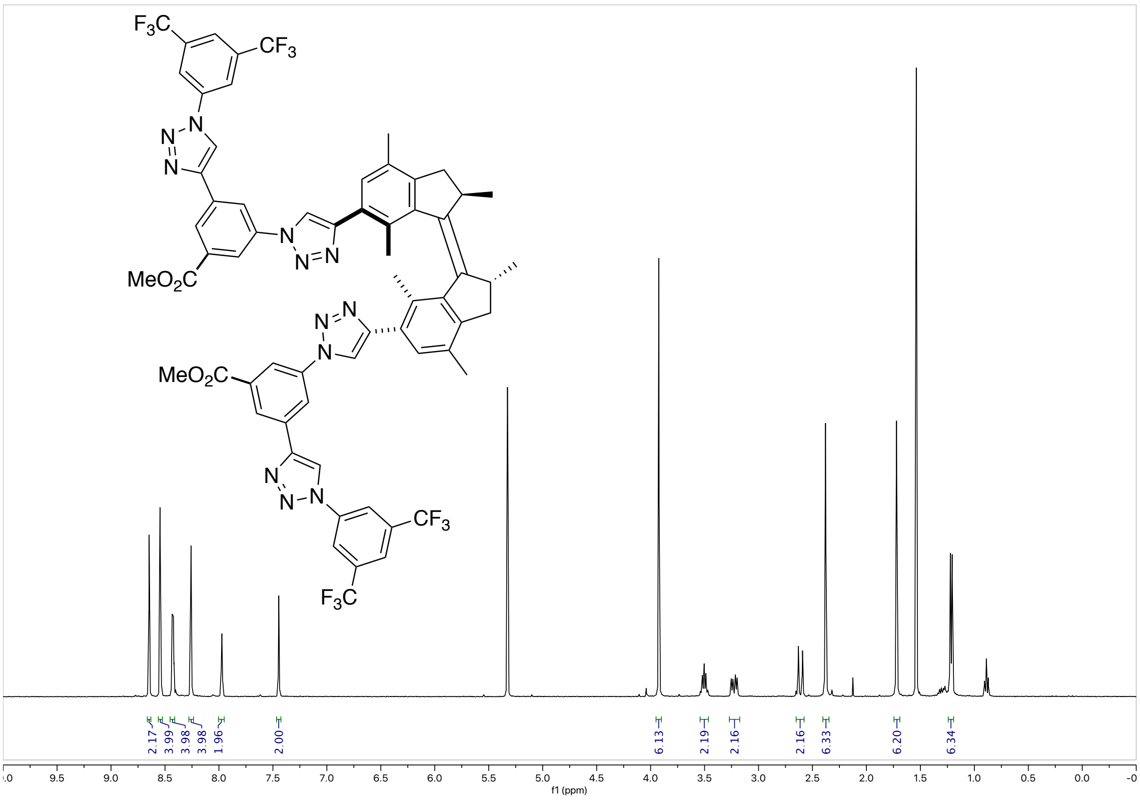

Figure S50. ^{1}H NMR (600 MHz, CD_2Cl_2) of ***(P,P)-cis*-1c.**

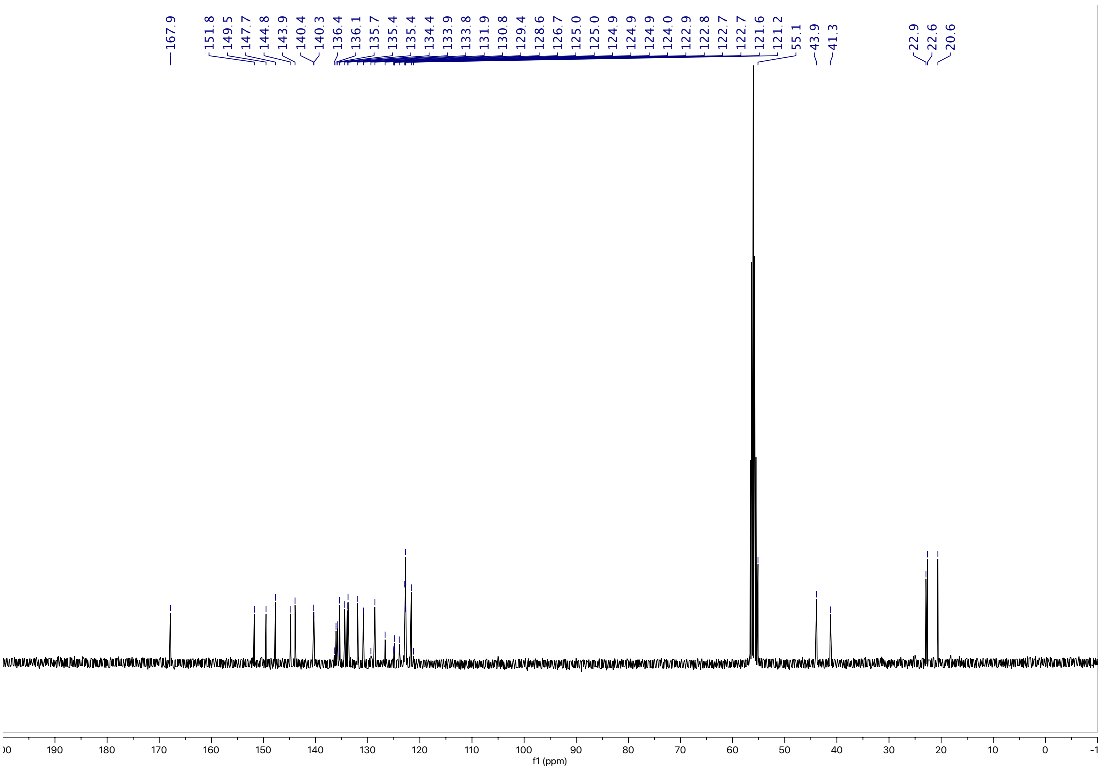

Figure S51. ^{13}C NMR (101 MHz, CD_2Cl_2) of ***(P,P)-cis*-1c**.

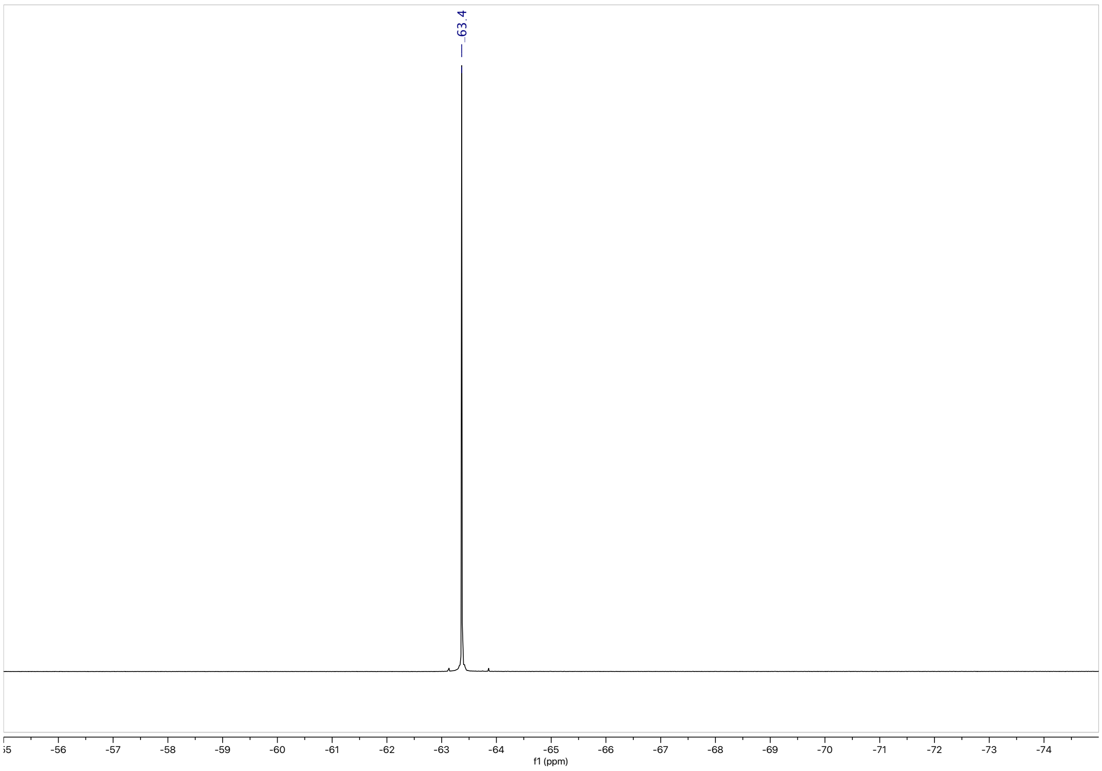

Figure S52. ^{19}F NMR (376 MHz, CD_2Cl_2) of ***(P,P)-cis*-1c**.

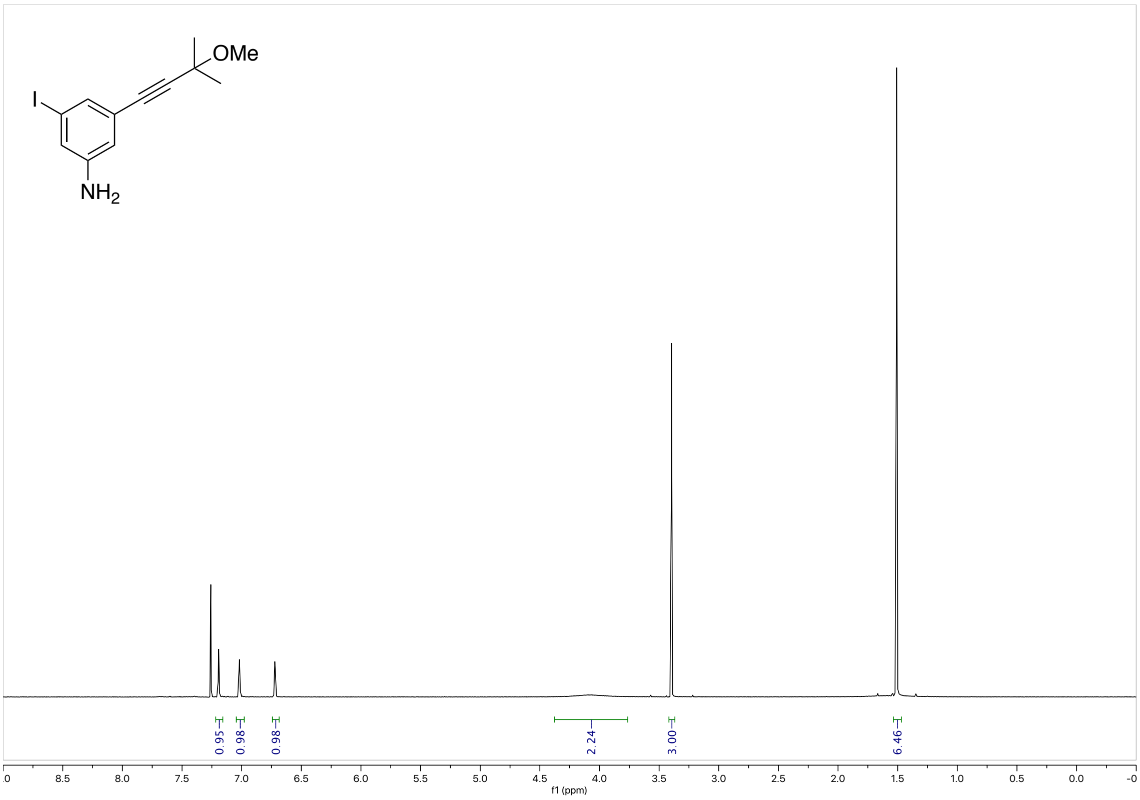

Figure S53. ^{1}H NMR (400 MHz, $CDCl_3$) of **S8.**

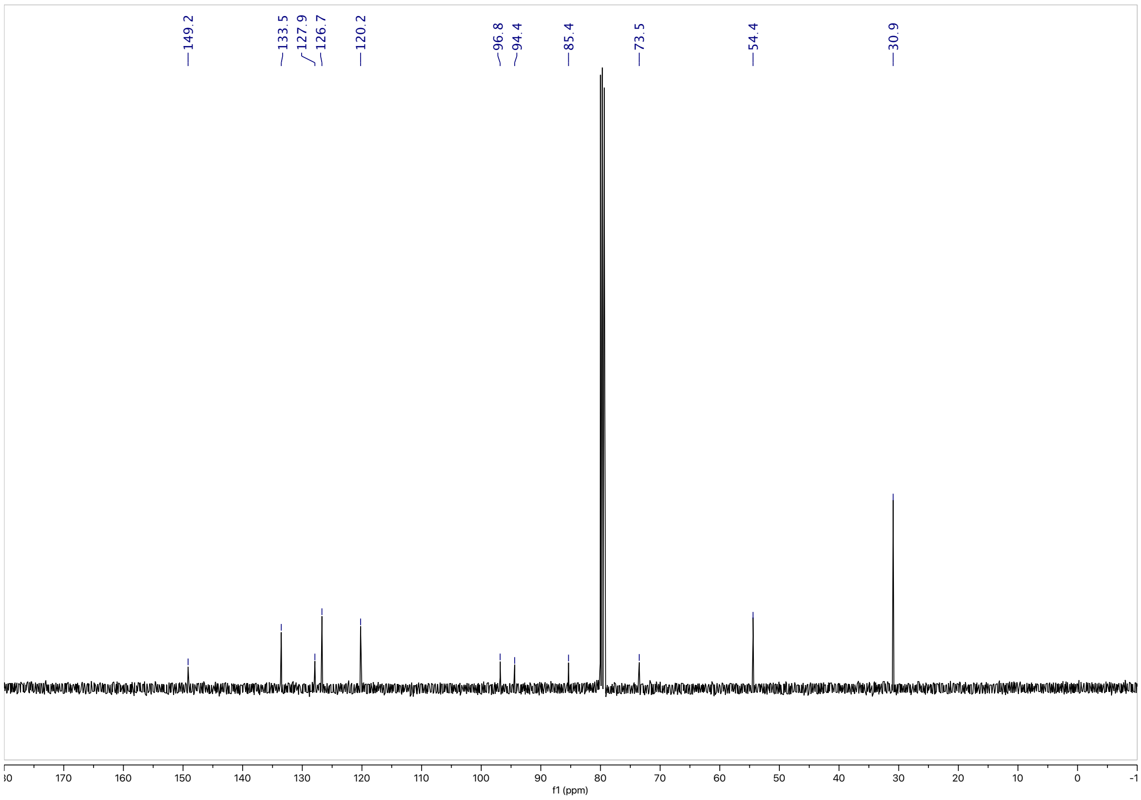

Figure S54. ^{13}C NMR (101 MHz, $CDCl_3$) of **S8.**

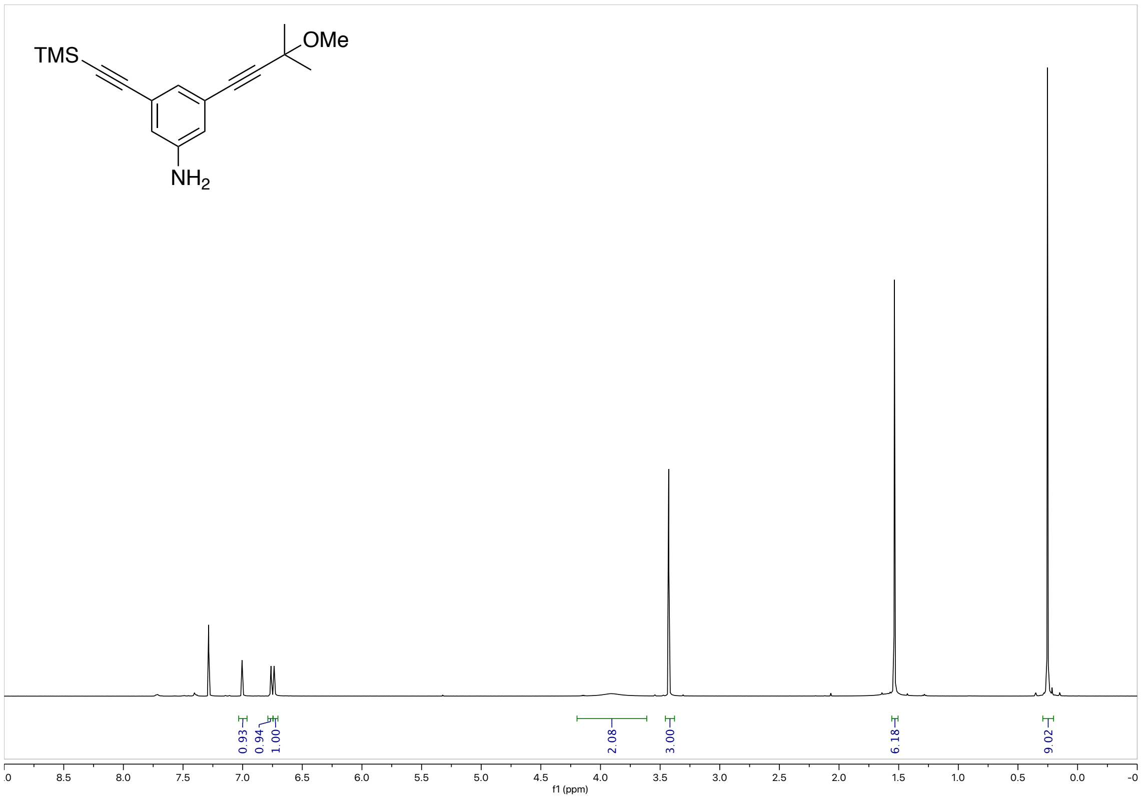

Figure S55. ^{1}H NMR (600 MHz, $CDCl_3$) of **S9.**

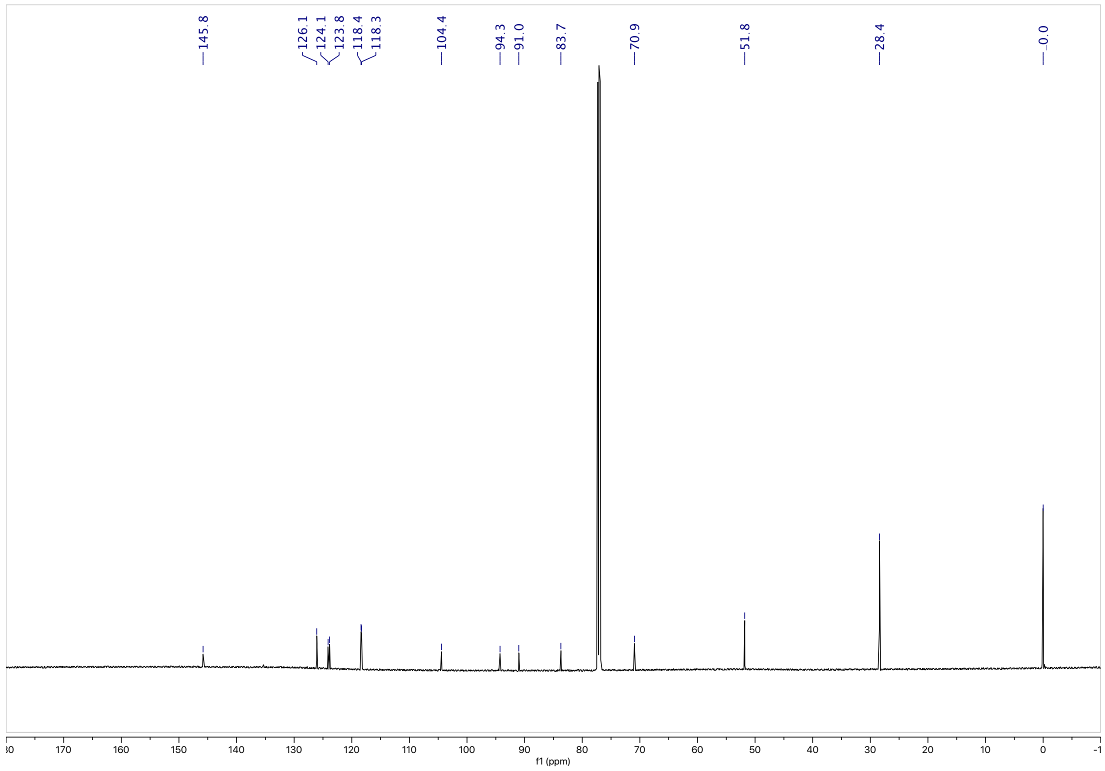

Figure S56. ^{13}C NMR (151 MHz, $CDCl_3$) of **S9.**

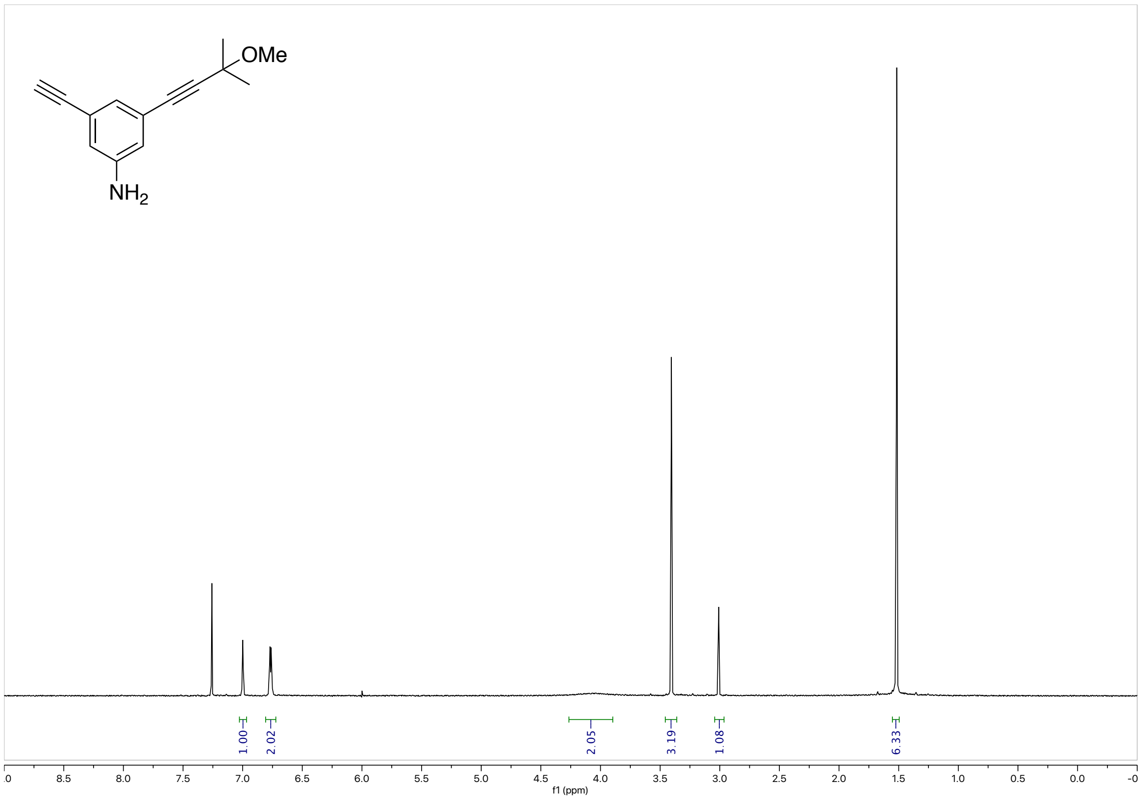

Figure S57. ^{1}H NMR (400 MHz, $CDCl_3$) of **S10.**

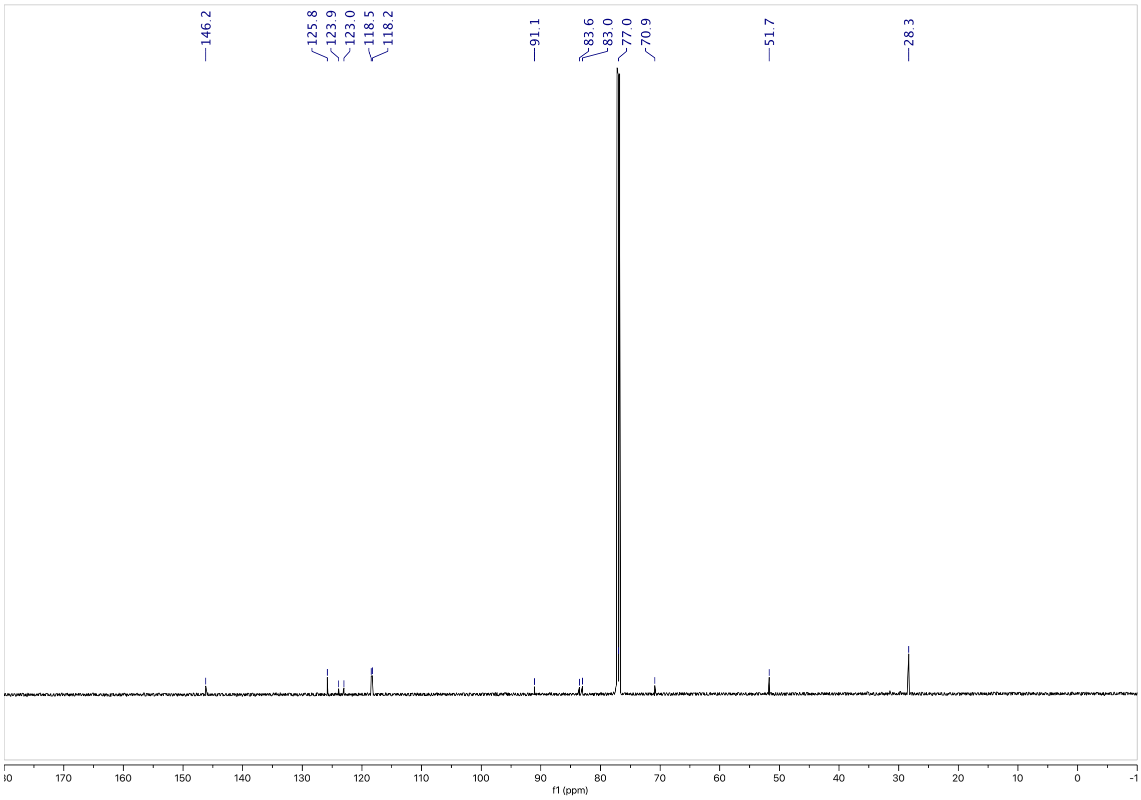

Figure S58. ^{13}C NMR (151 MHz, $CDCl_3$) of **S10.**

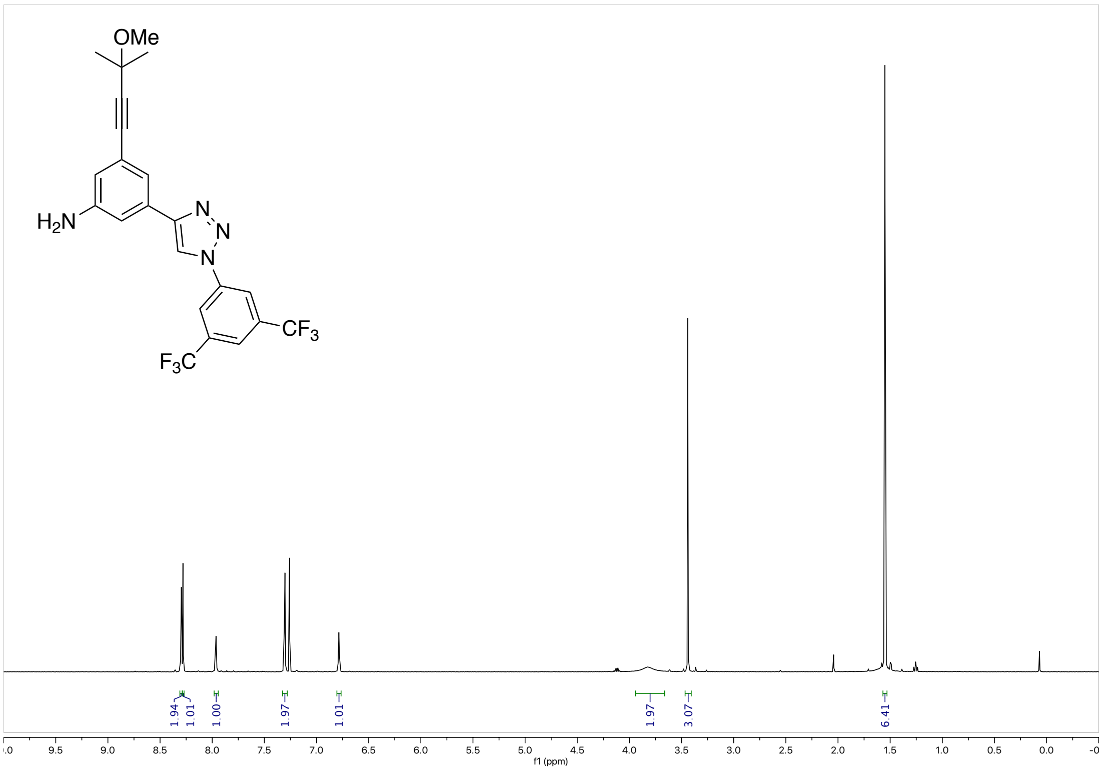

Figure S59. ^{1}H NMR (400 MHz, $CDCl_3$) of **S11.**

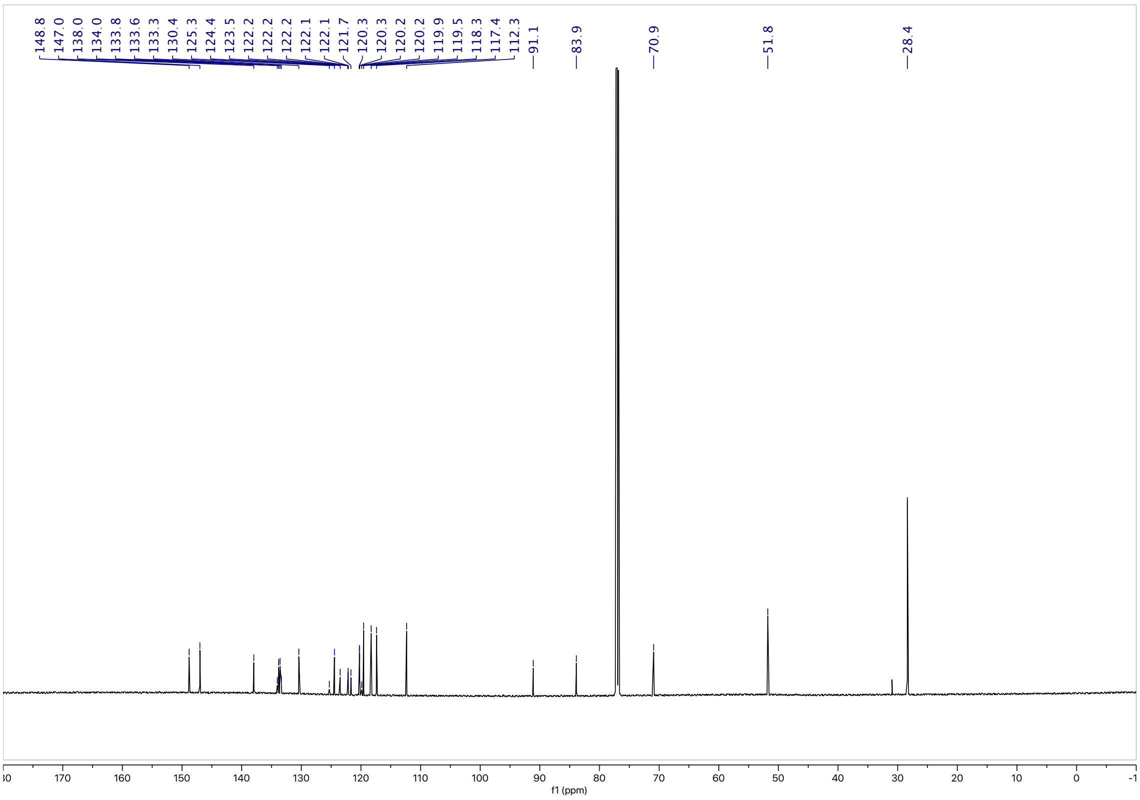

Figure S60. ^{13}C NMR (151 MHz, $CDCl_3$) of **S11.**

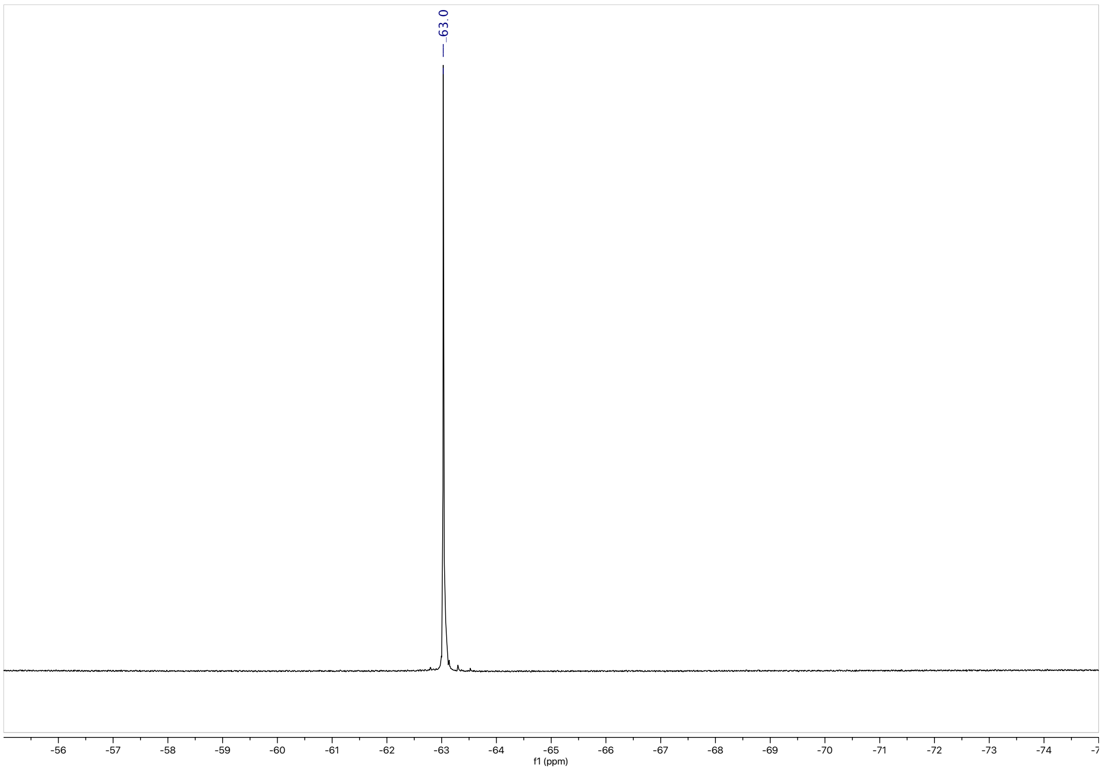

Figure S61. ^{19}F NMR (376 MHz, $CDCl_3$) of **S11.**

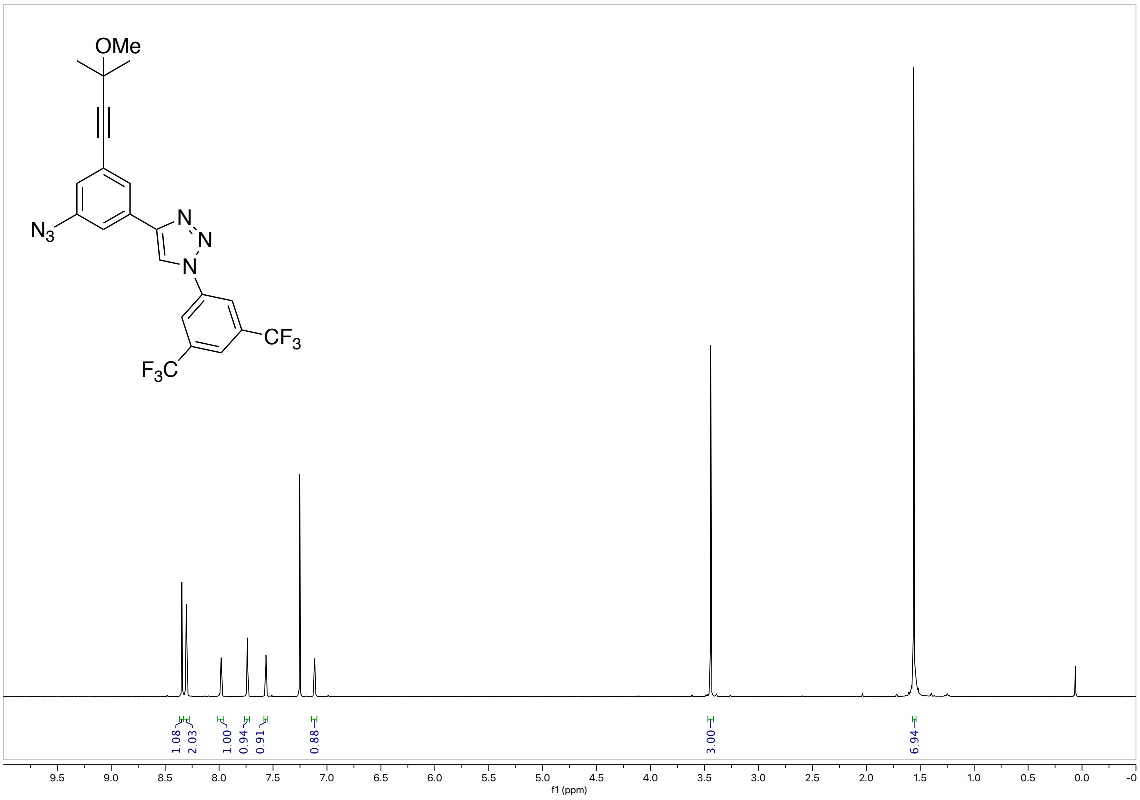

Figure S62. ^{1}H NMR (400 MHz, $CDCl_3$) of **5d.**

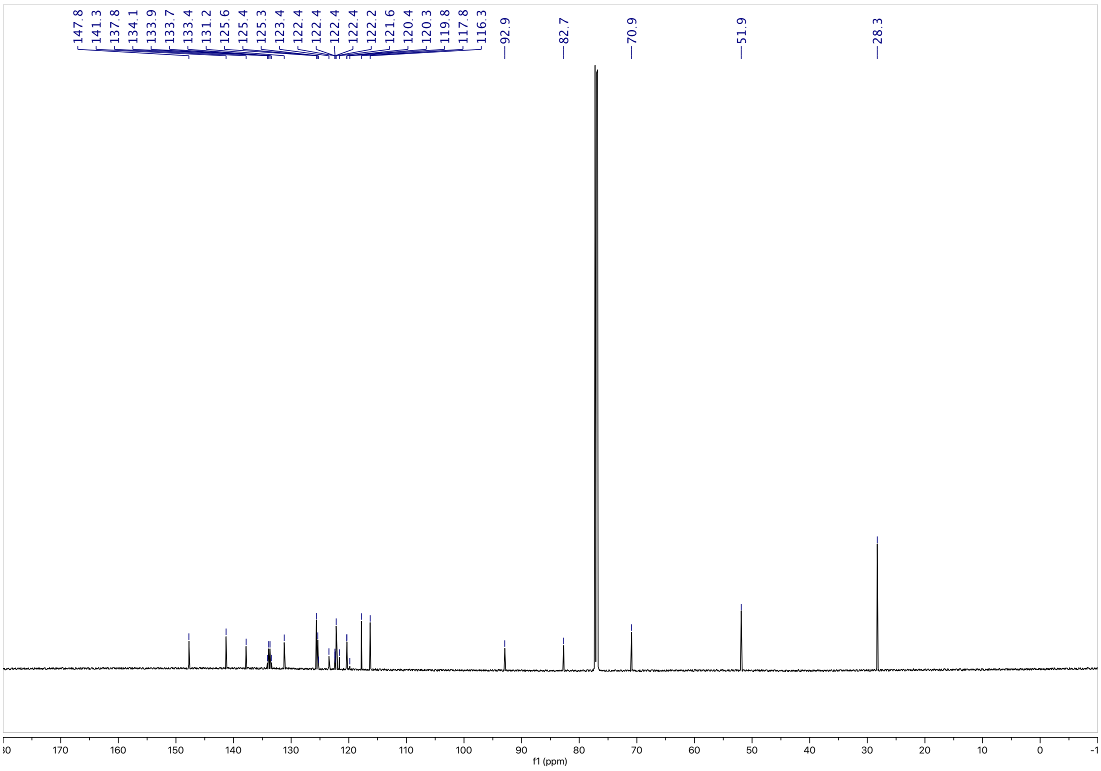

Figure S63. ^{13}C NMR (151 MHz, $CDCl_3$) of **5d.**

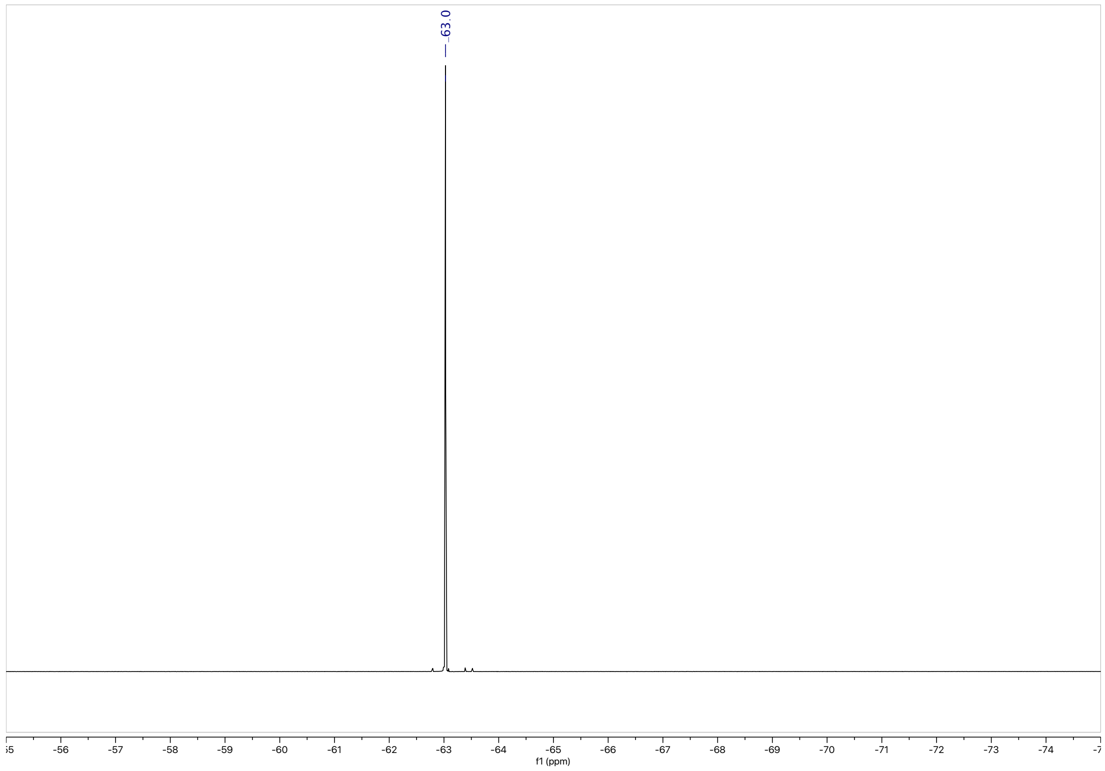

Figure S64. ^{19}F NMR (376 MHz, $CDCl_3$) of **5d.**

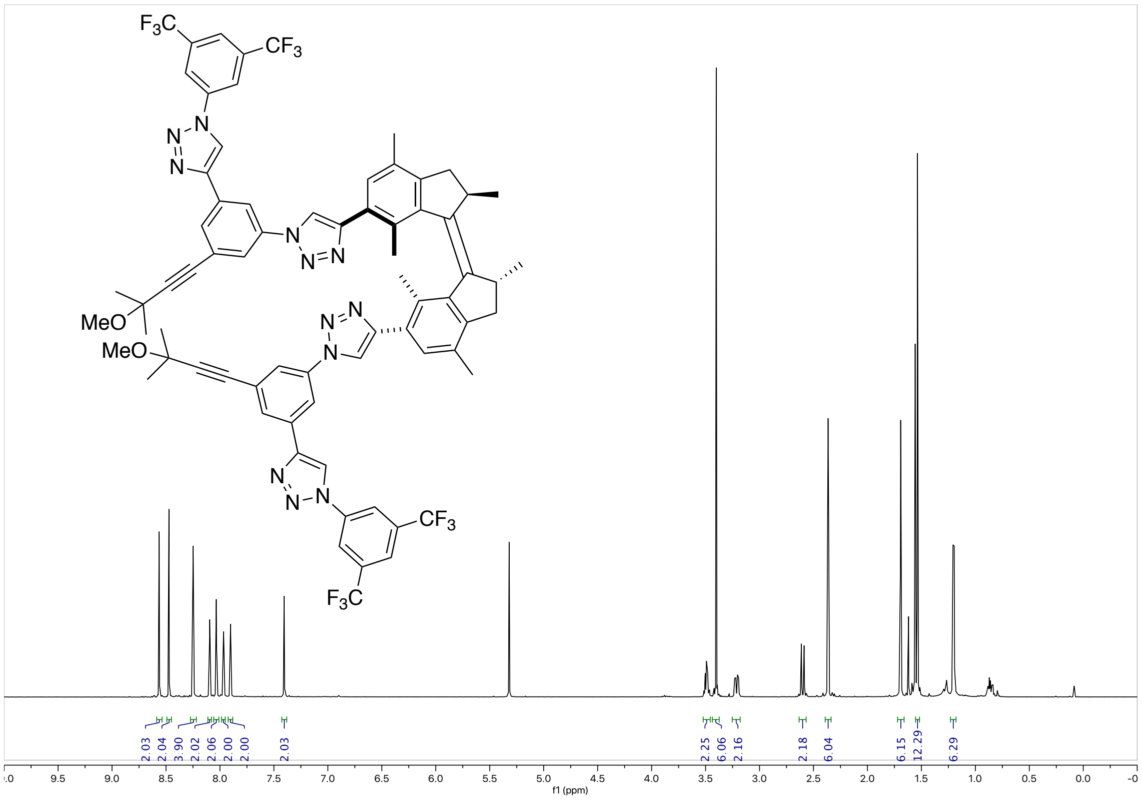

Figure S65. ^{1}H NMR (600 MHz, CD_2Cl_2) of ***(P,P)-cis*-1d.**

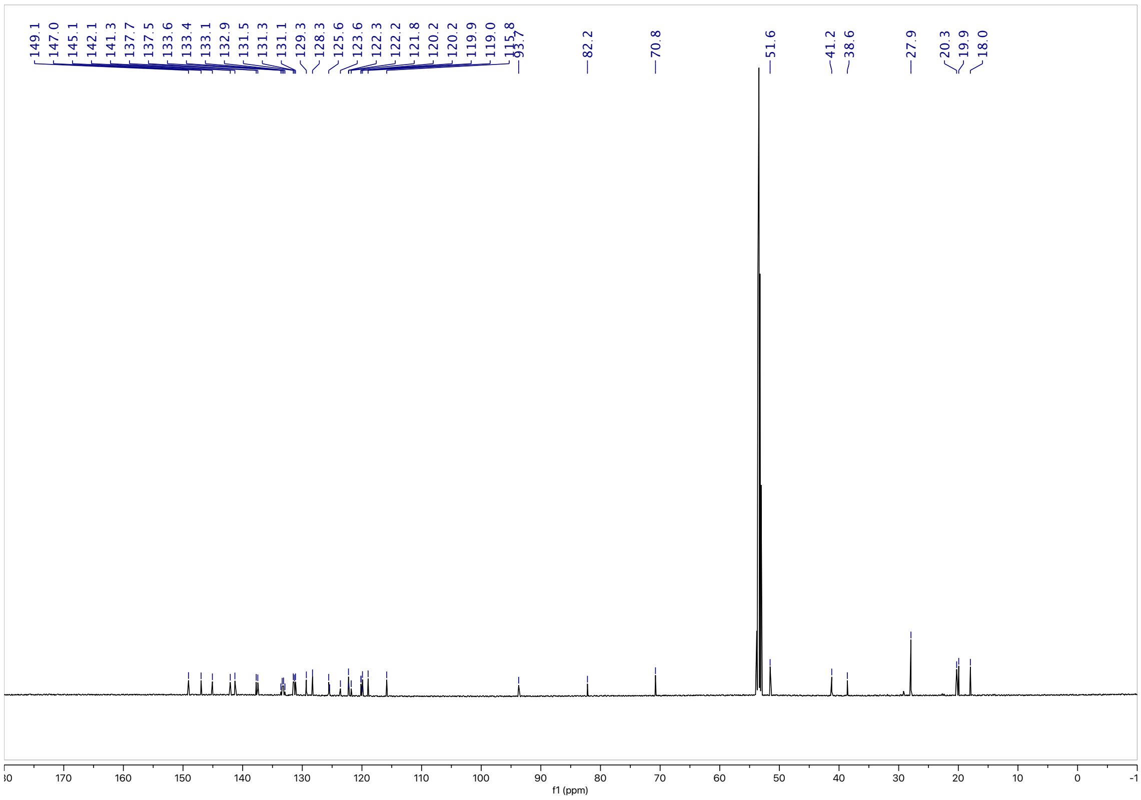

Figure S66. ^{13}C NMR (151 MHz, CD_2Cl_2) of ***(P,P)-cis*-1d**.

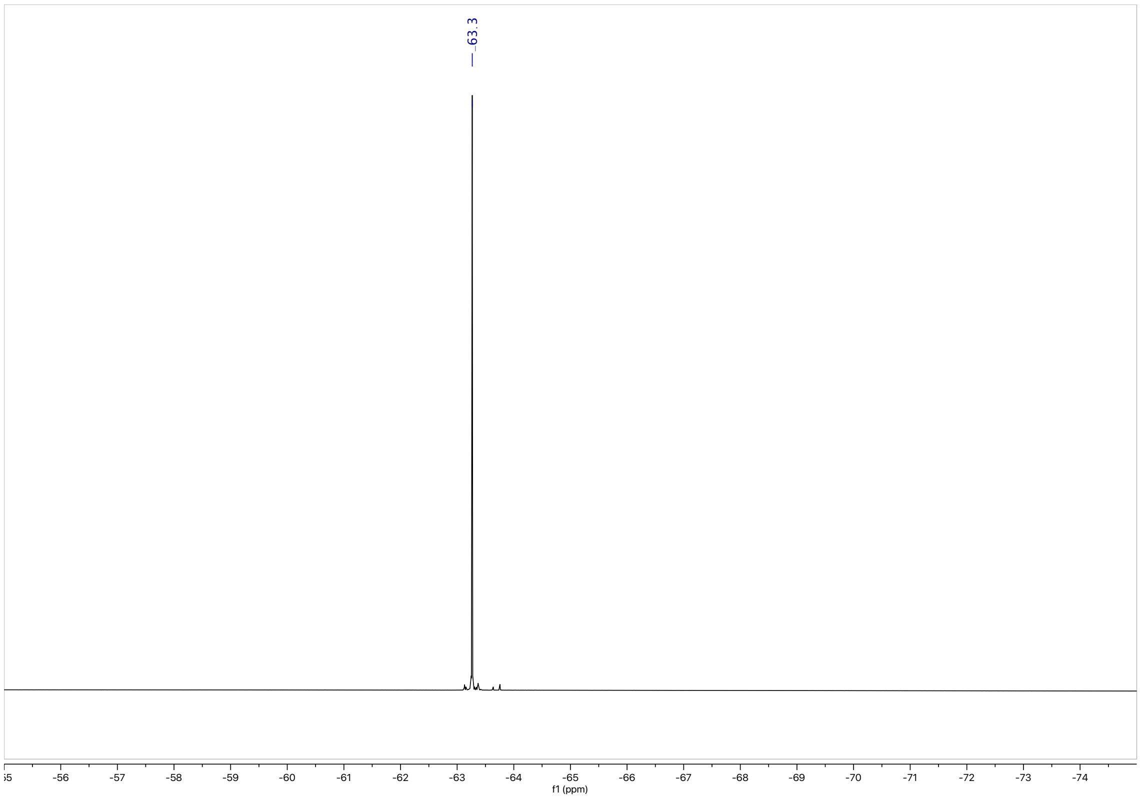

Figure S67. ^{19}F NMR (565 MHz, CD_2Cl_2) of ***(P,P)-cis*-1d**.

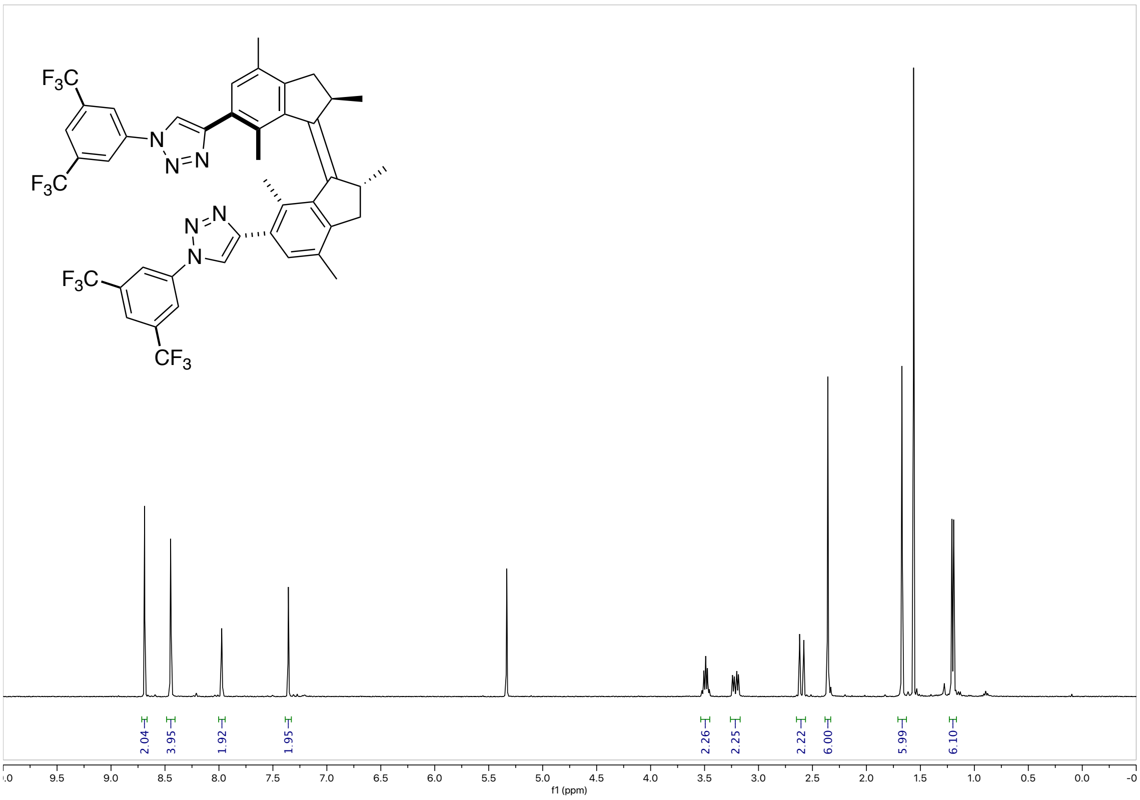

Figure S68. ^{1}H NMR (400 MHz, CD_2Cl_2) of ***(P,P)-cis*-7**.

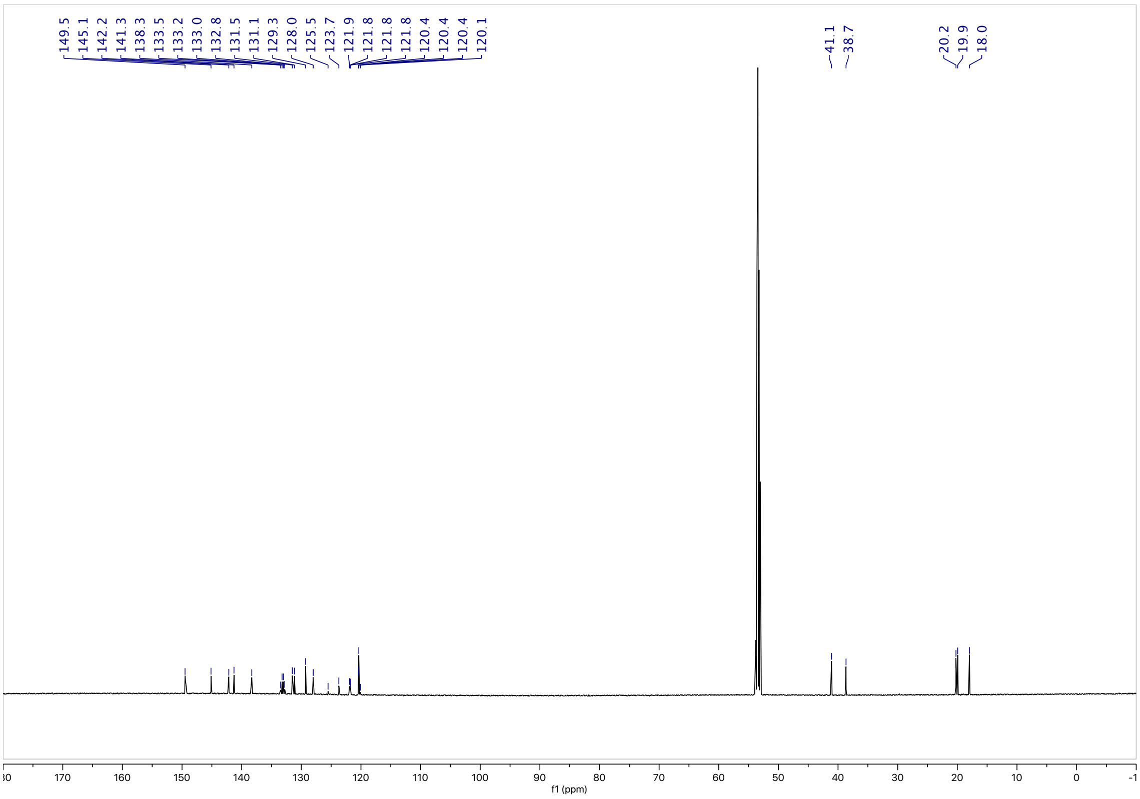

Figure S69. ^{13}C NMR (151 MHz, CD_2Cl_2) of ***(P,P)-cis*-7**.

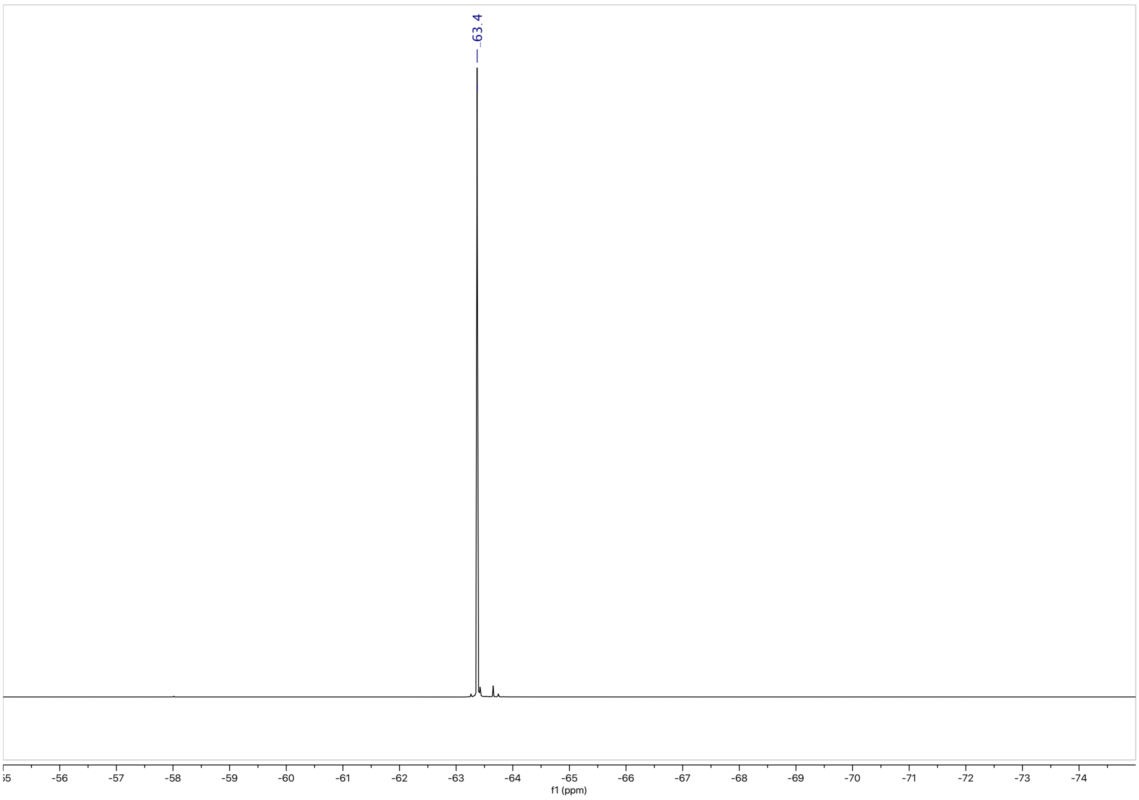

Figure S70. ^{19}F NMR (565 MHz, CD_2Cl_2) of ***(P,P)-cis*-7**.

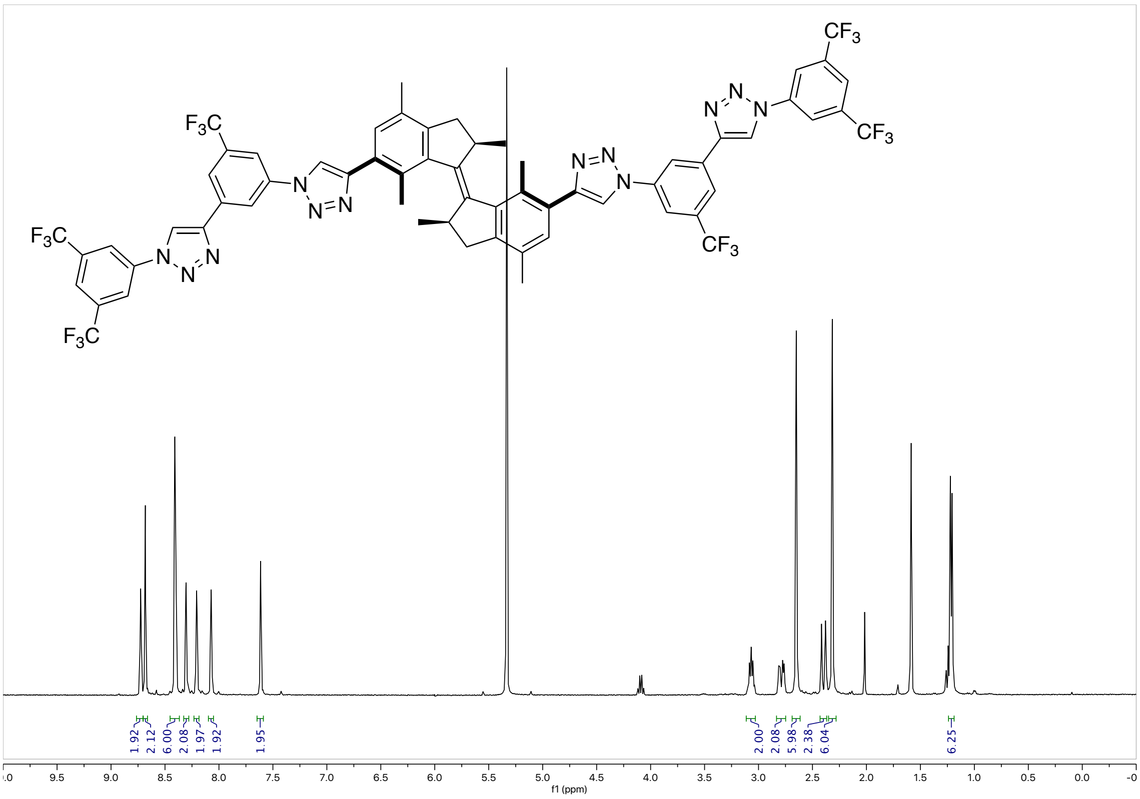

Figure S71. ^{1}H NMR (400 MHz, CD_2Cl_2) of ***(P,P)-trans*-1c.**

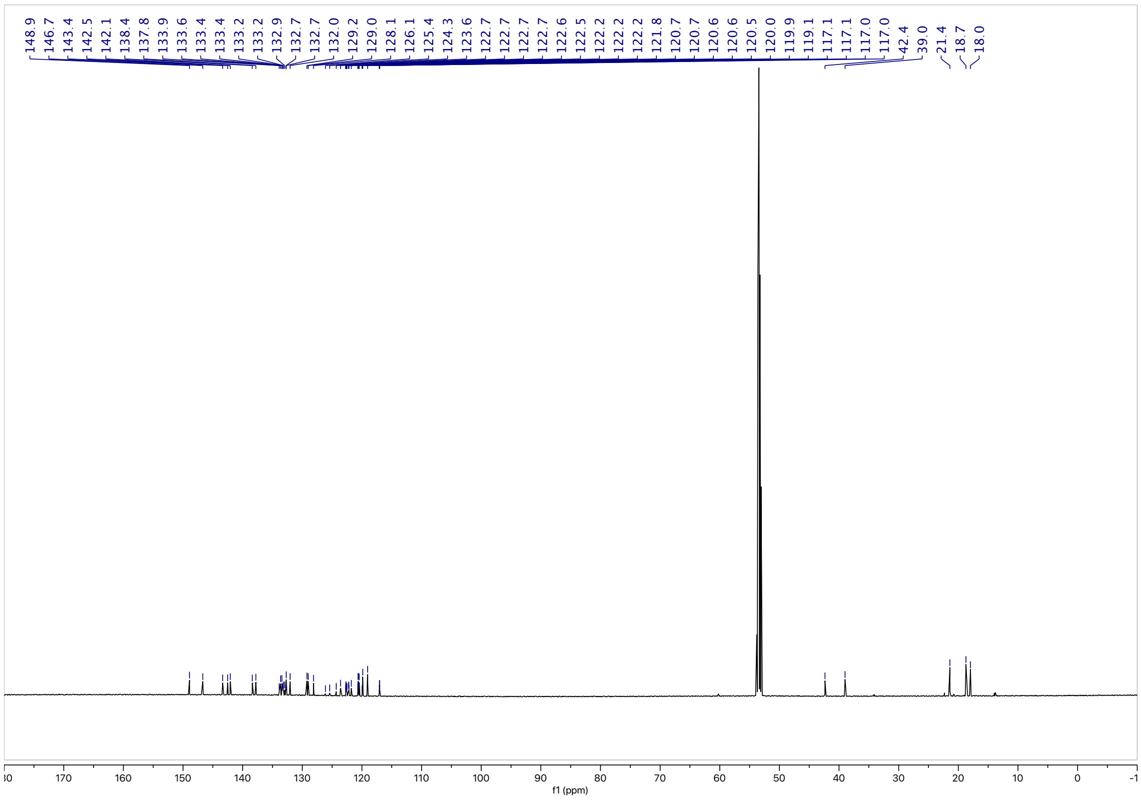

Figure S72. ^{13}C NMR (151 MHz, CD_2Cl_2) of ***(P,P)*-*trans*-1c**.

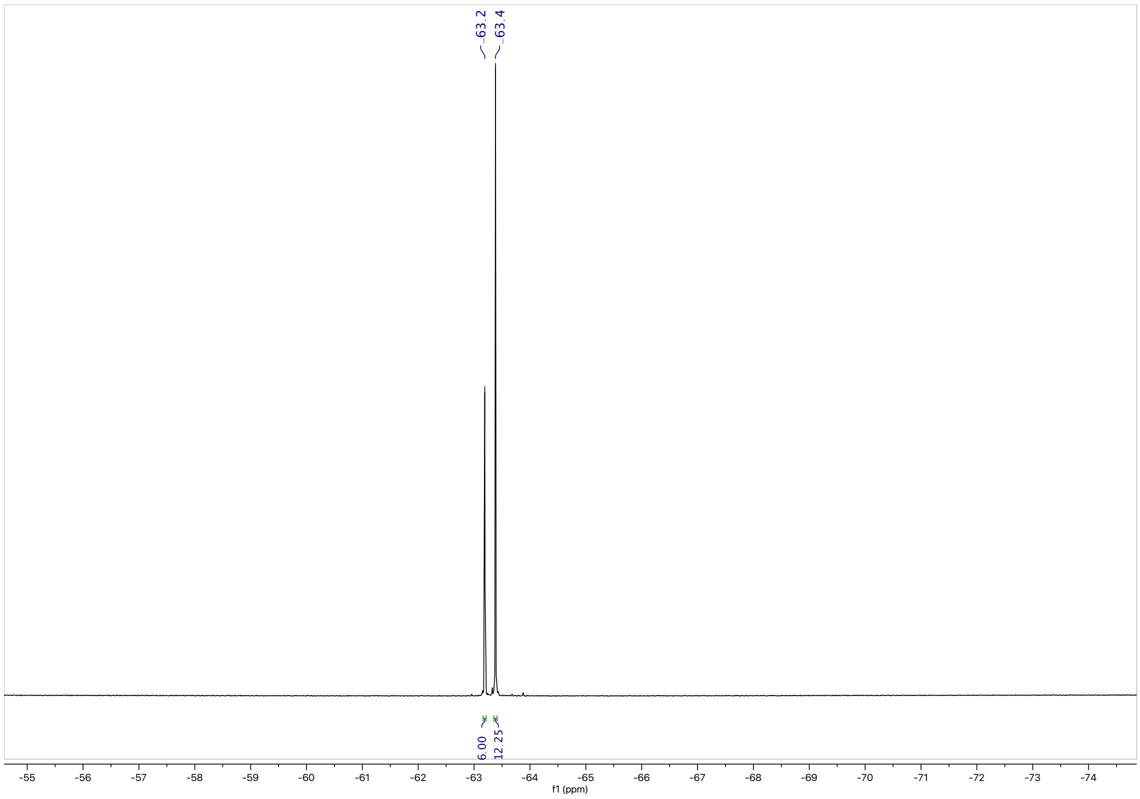

Figure S73. ^{19}F NMR (376 MHz, CD_2Cl_2) of ***(P,P)*-*trans*-1c**.

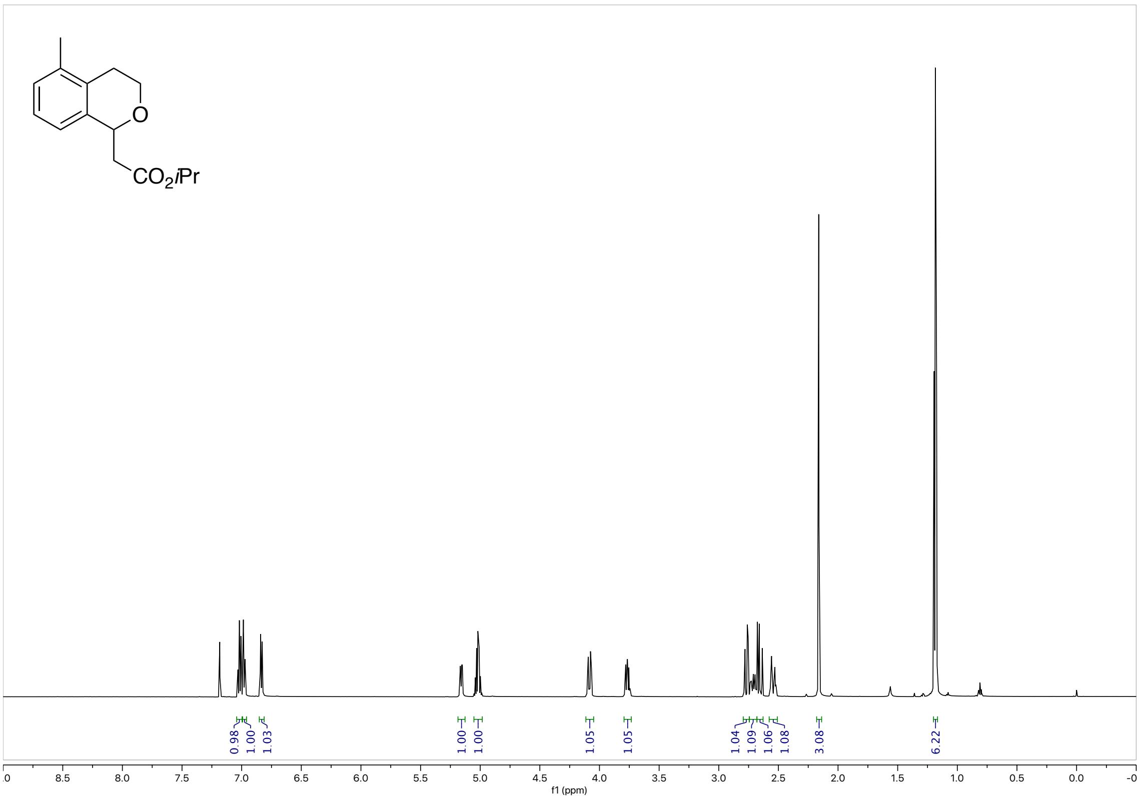

Figure S74. ^{1}H NMR (600 MHz, $CDCl_3$) of **10b.**

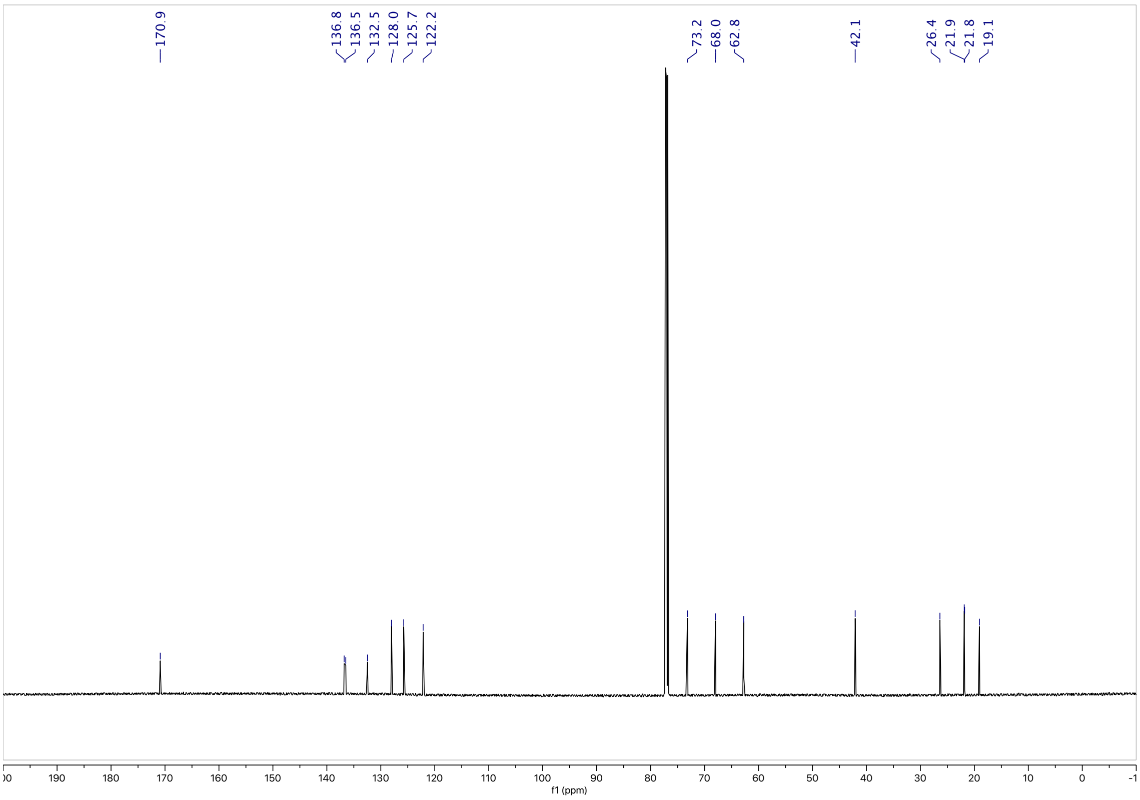

Figure S75. ^{13}C NMR (151 MHz, $CDCl_3$) of **10b.**

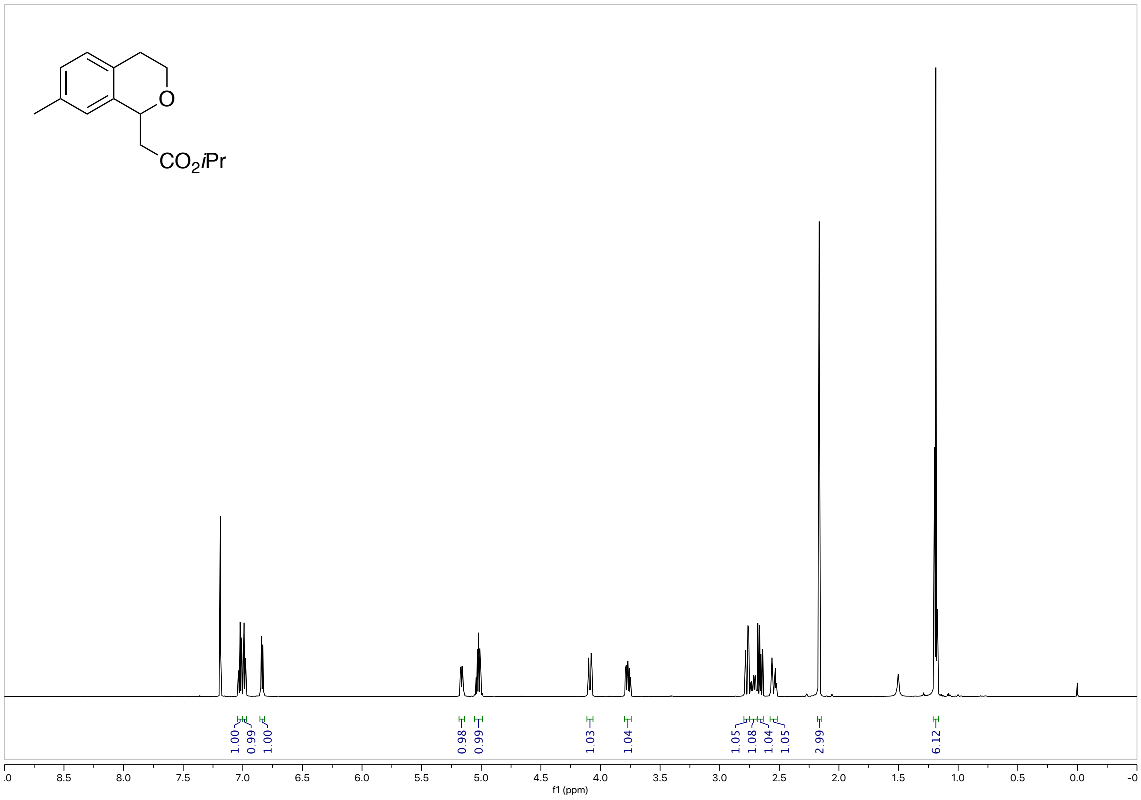

Figure S76. ^{1}H NMR (600 MHz, $CDCl_3$) of **10c.**

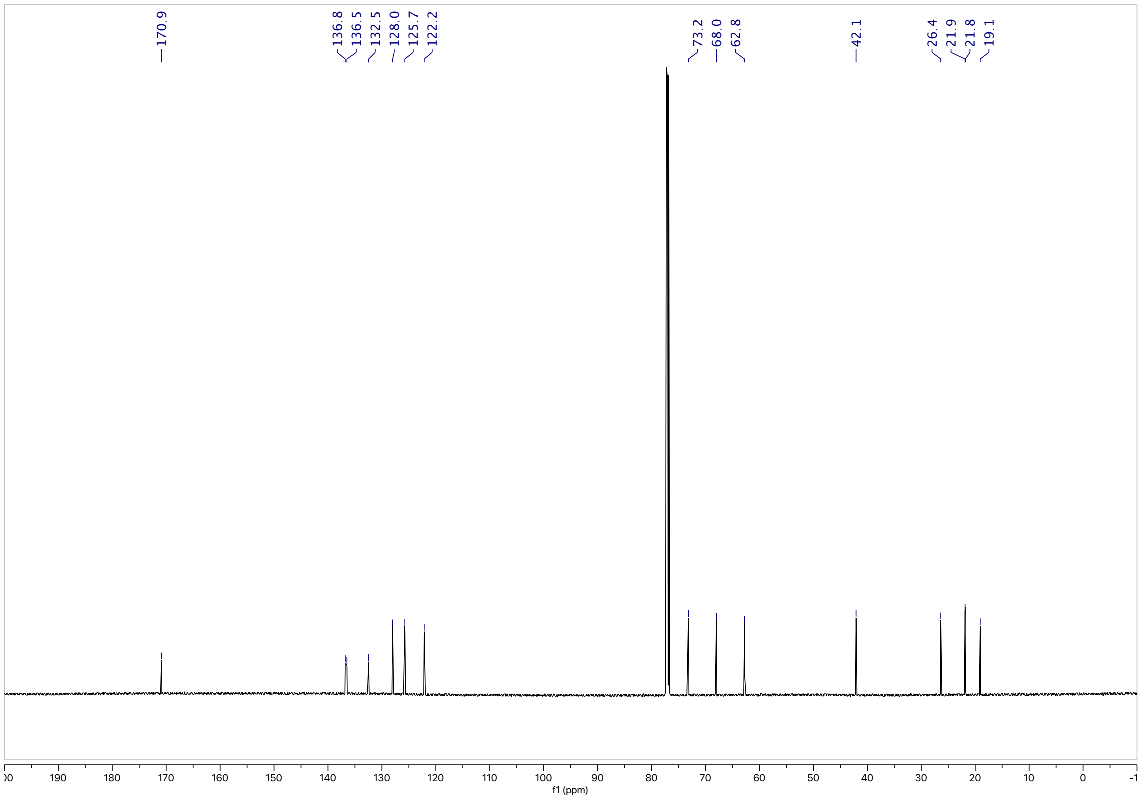

Figure S77. ^{13}C NMR (151 MHz, $CDCl_3$) of **10c.**

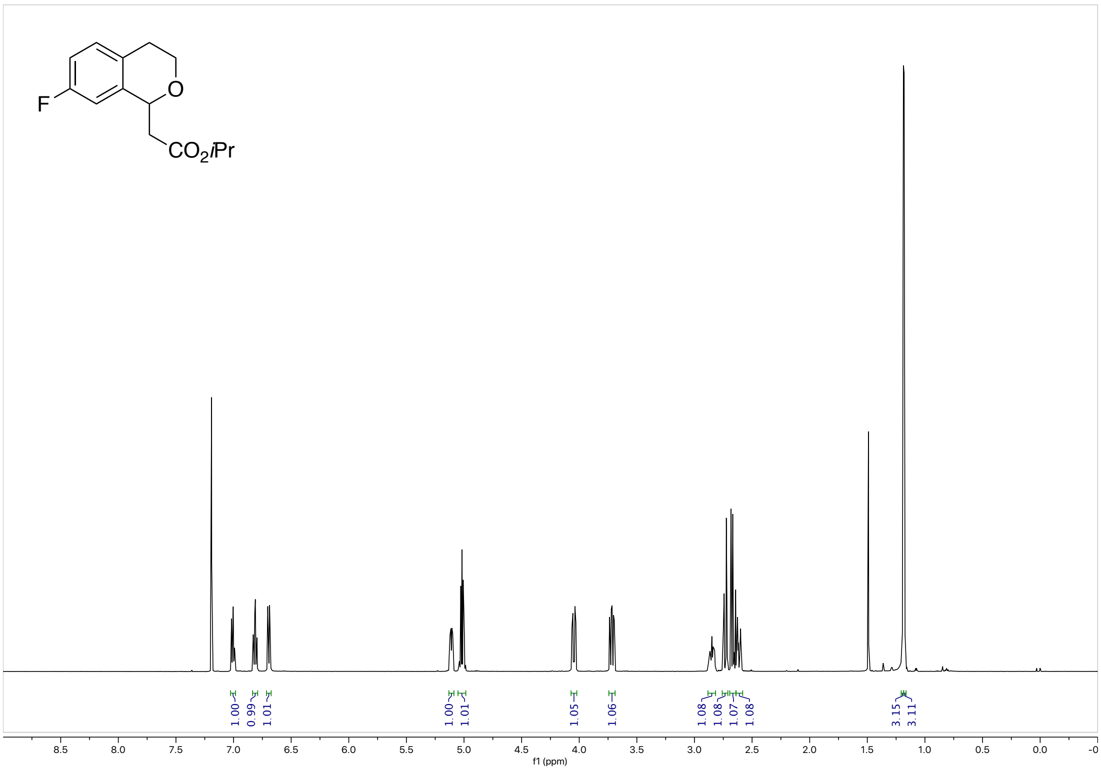

Figure S78. ^{1}H NMR (600 MHz, $CDCl_3$) of **10d.**

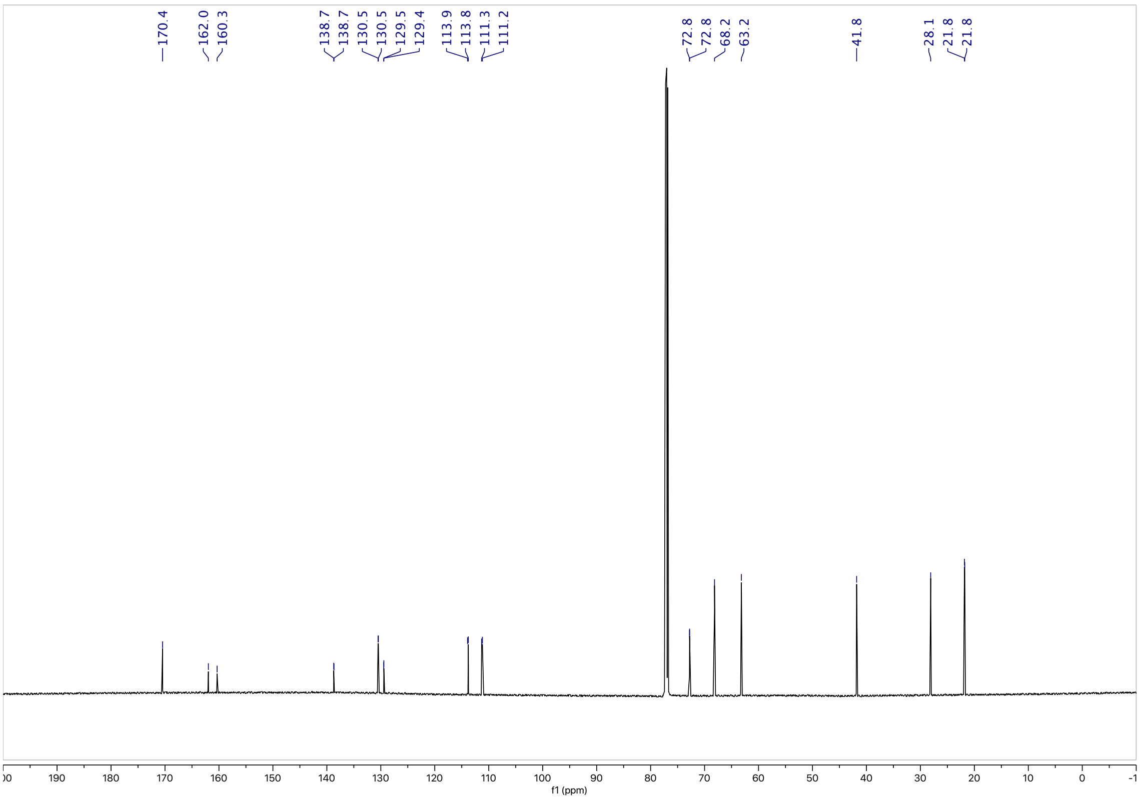

Figure S79. ^{13}C NMR (151 MHz, $CDCl_3$) of **10d.**

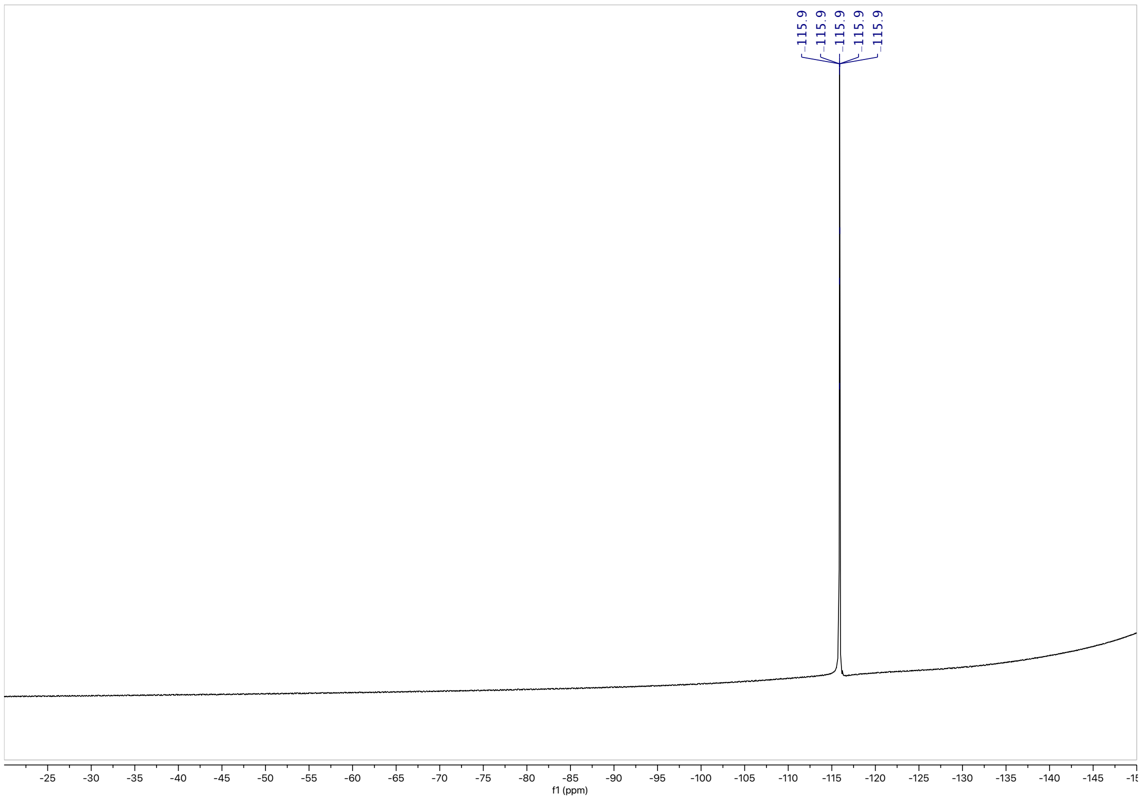

Figure S80. ^{19}F NMR (565 MHz, $CDCl_3$) of **10d.**

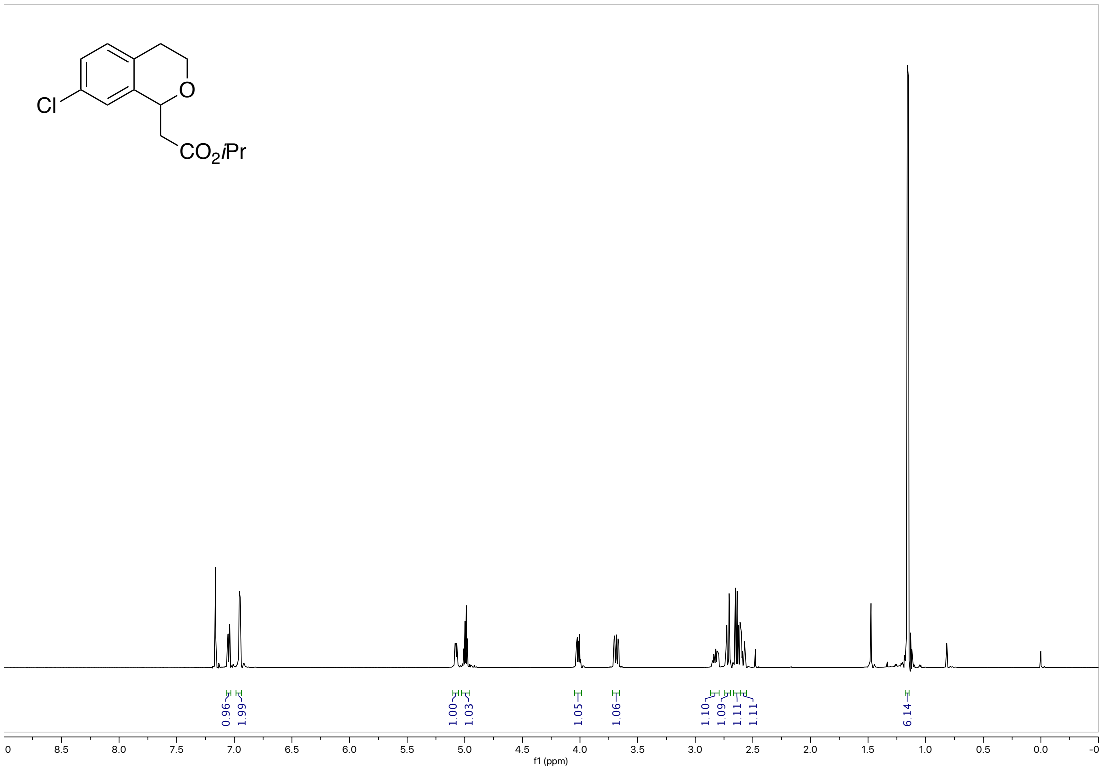

Figure S81. ^{1}H NMR (600 MHz, $CDCl_3$) of **10e.**

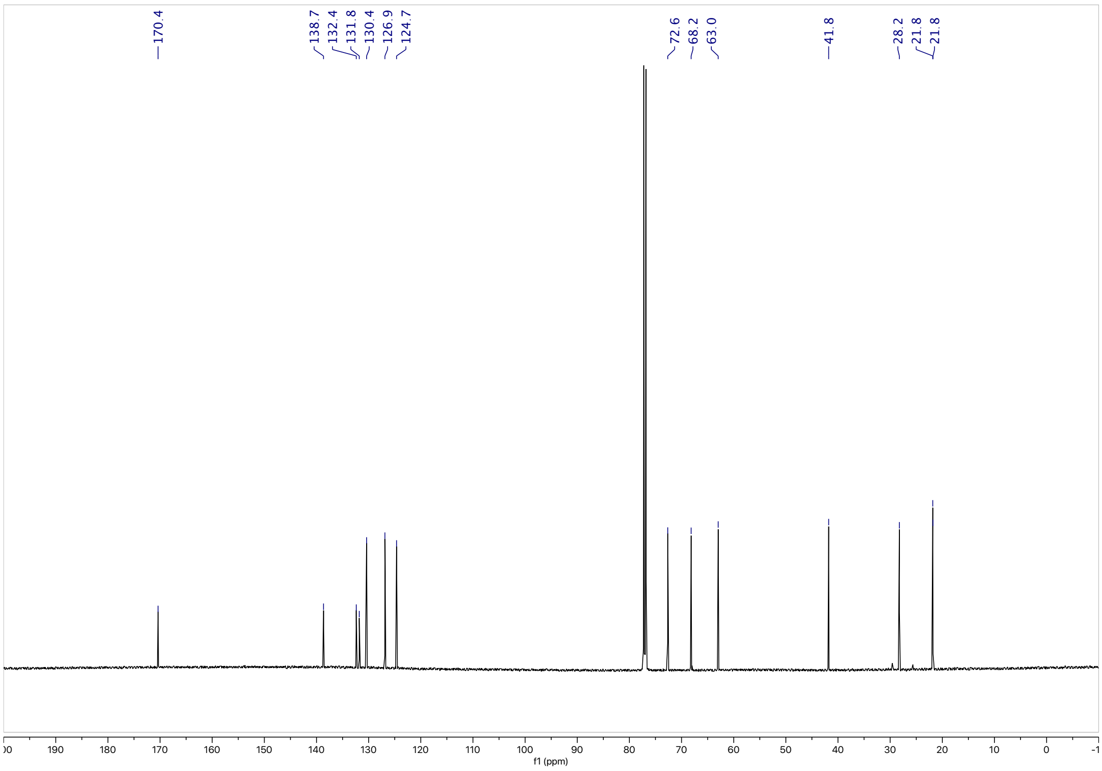

Figure S82. ^{13}C NMR (151 MHz, $CDCl_3$) of **10e.**

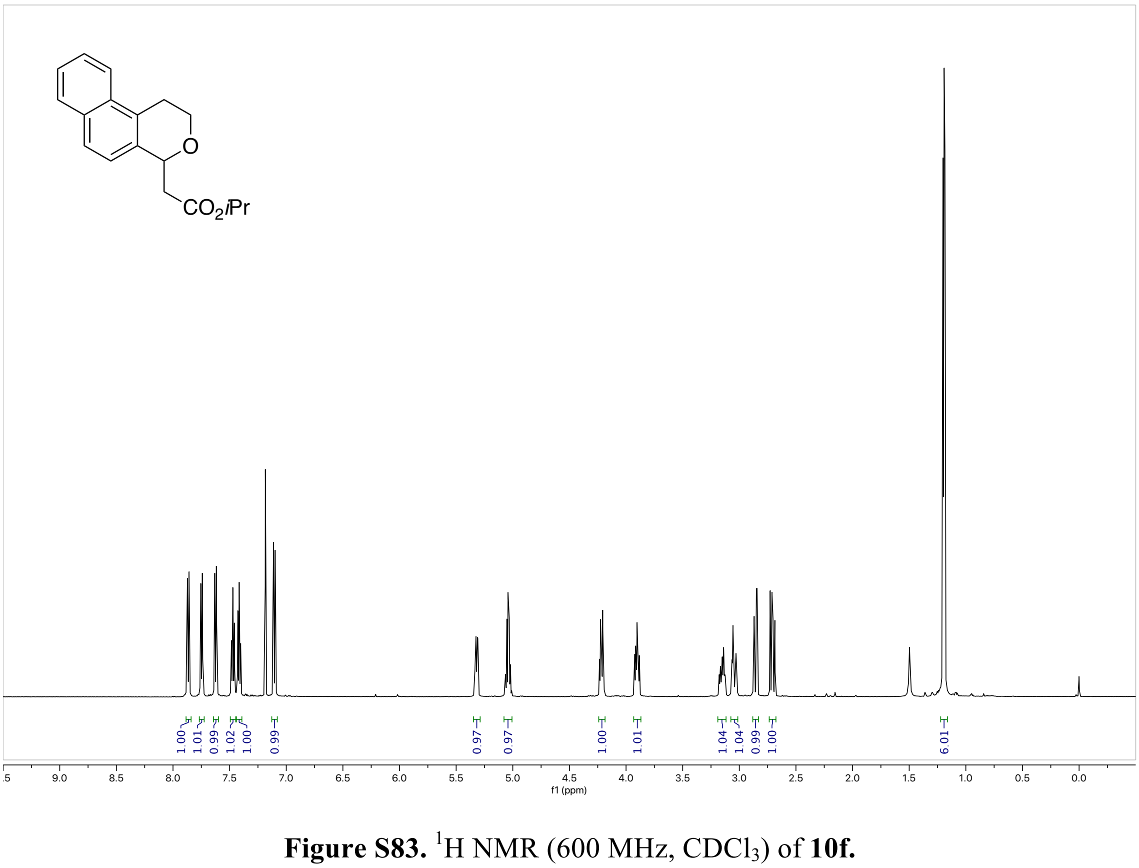

Figure S83. ^{1}H NMR (600 MHz, $CDCl_3$) of **10f.**

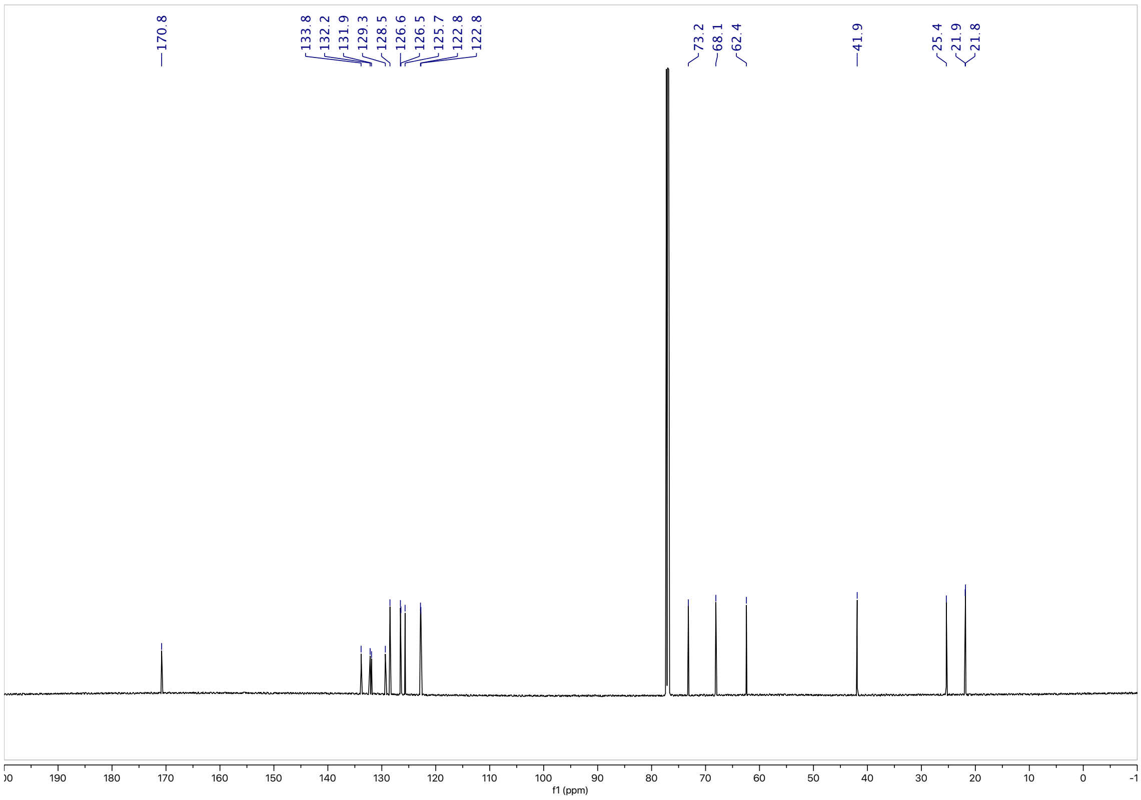

Figure S84. ^{13}C NMR (151 MHz, $CDCl_3$) of **10f.**

7. HPLC and SFC traces

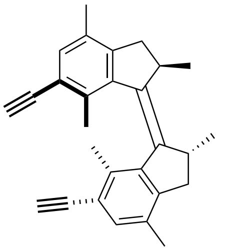

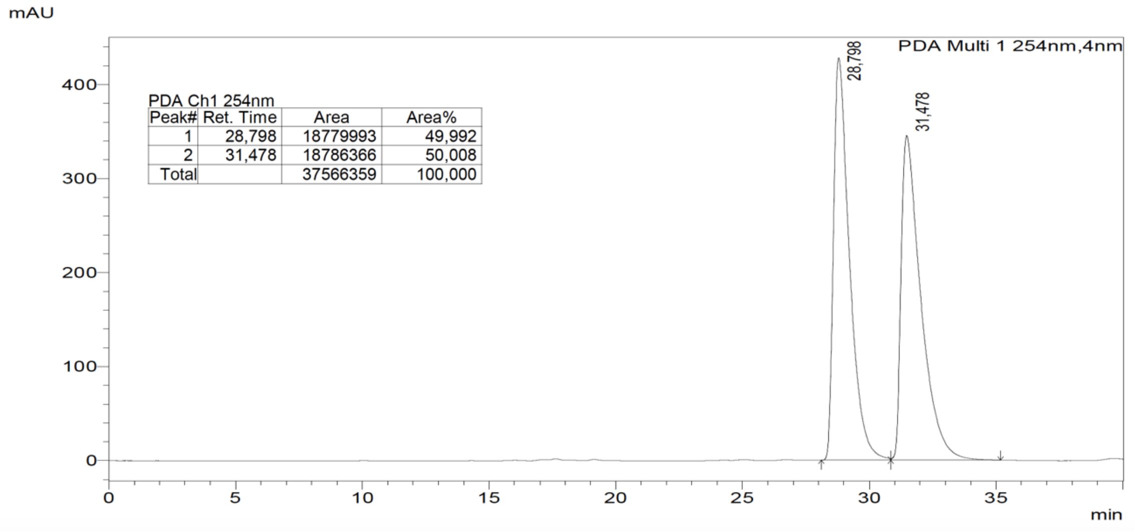

Figure S85. HPLC trace of *rac*-(*P*,*P*)-***cis*-4**.

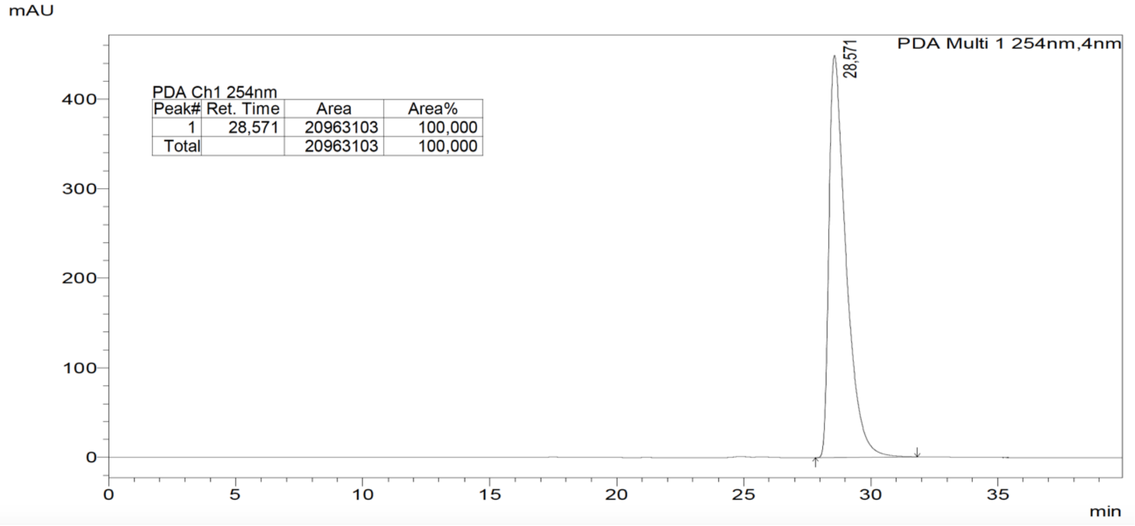

Figure S86. HPLC trace of (*R*,*R*)-(*P*,*P*)-*cis*-**4**.

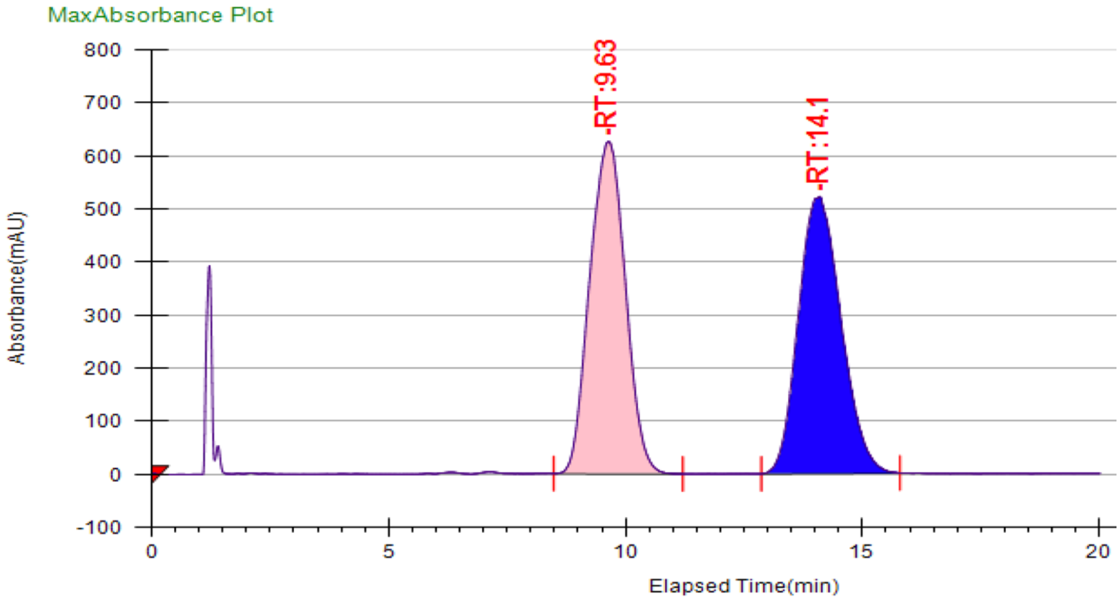

Peak Information

Peak No	% Area	Area	Ret. Time
1	50.0886	32690.530 9	9.63 min
2	49.9114	32574.875	14.1 min

Figure S87. SFC trace of *rac*-(*P*,*P*)-*cis*-**1b**.

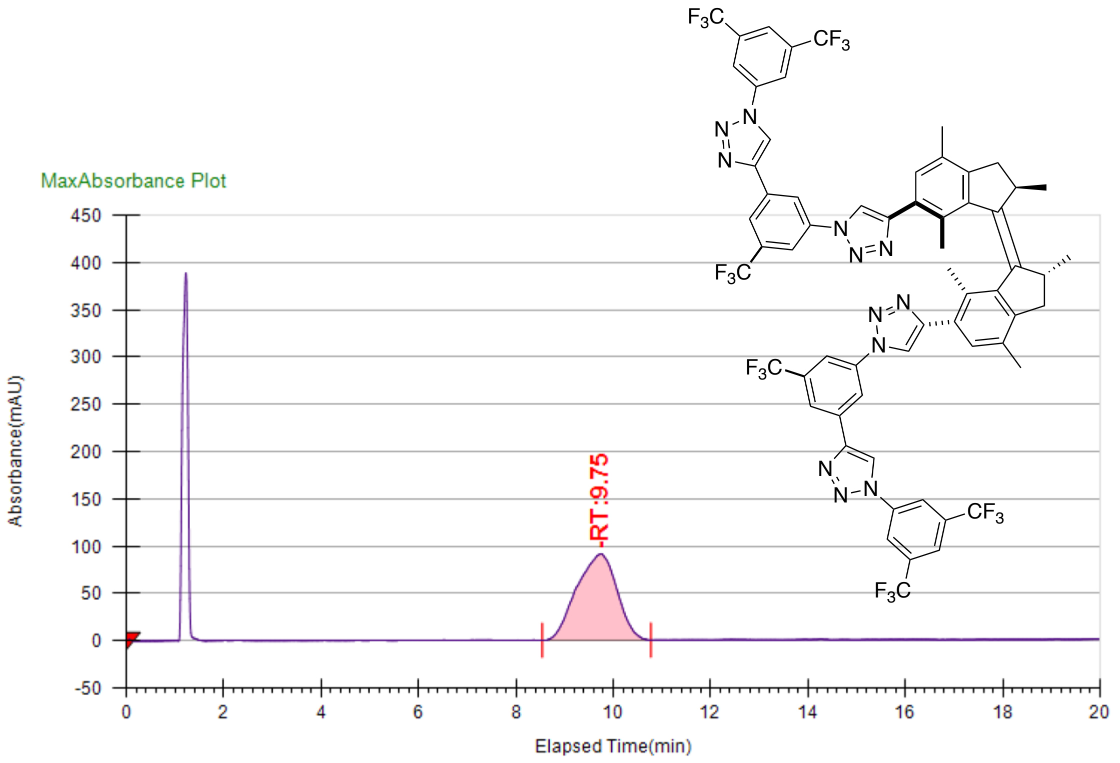

Peak Information

Peak No	% Area	Area	Ret. Time
1	100	5215.7051	9.75 min

Figure S88. SFC trace of (*R*,*R*)-(*P*,*P*)-*cis*-**1b**.

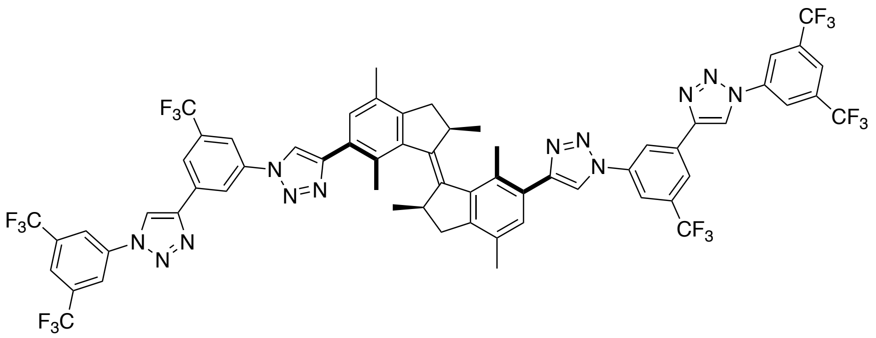

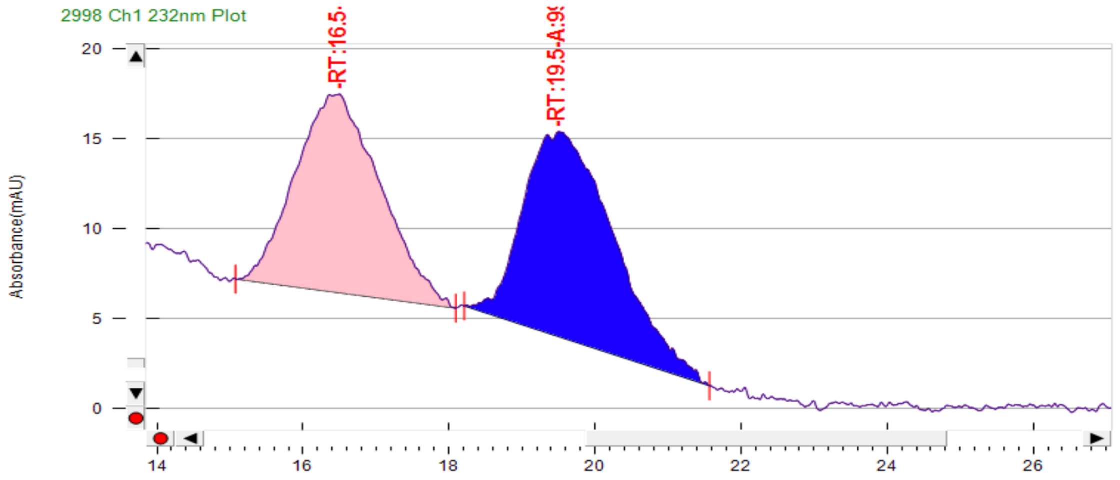

Peak Information

Peak No	% Area	Area	Ret. Time
1	47.2334	892.959	16.5 min
2	52.7666	997.5638	19.5 min

Figure S89. SFC trace of *rac*-(*P*,*P*)-*trans*-**1b**.

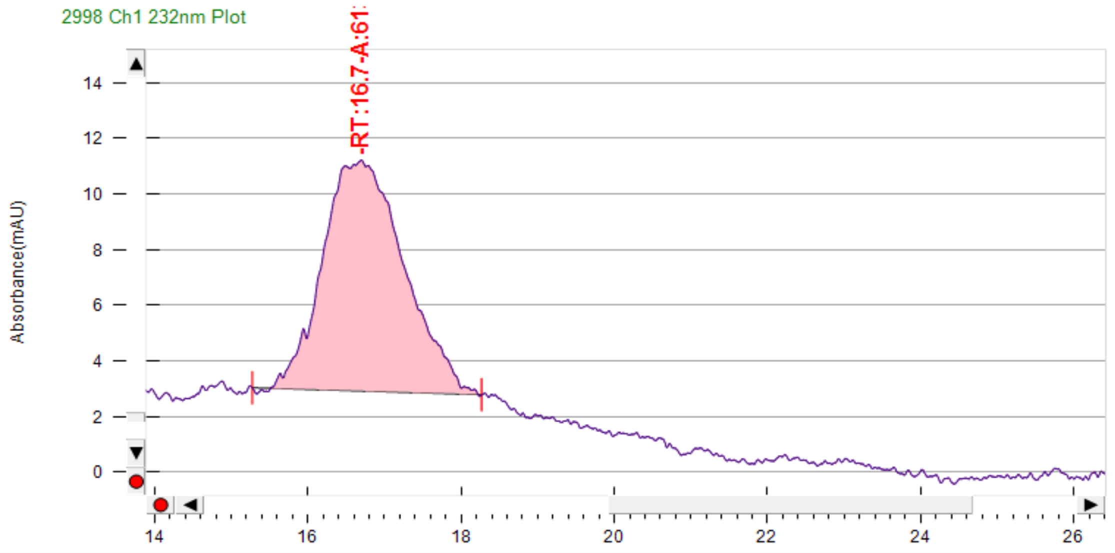

Peak Information

Peak No	% Area	Area	Ret. Time
1	100	613.9856	16.7 min

Figure S90. SFC trace of (*R*,*R*)-(*P*,*P*)-*trans*-**1b**.

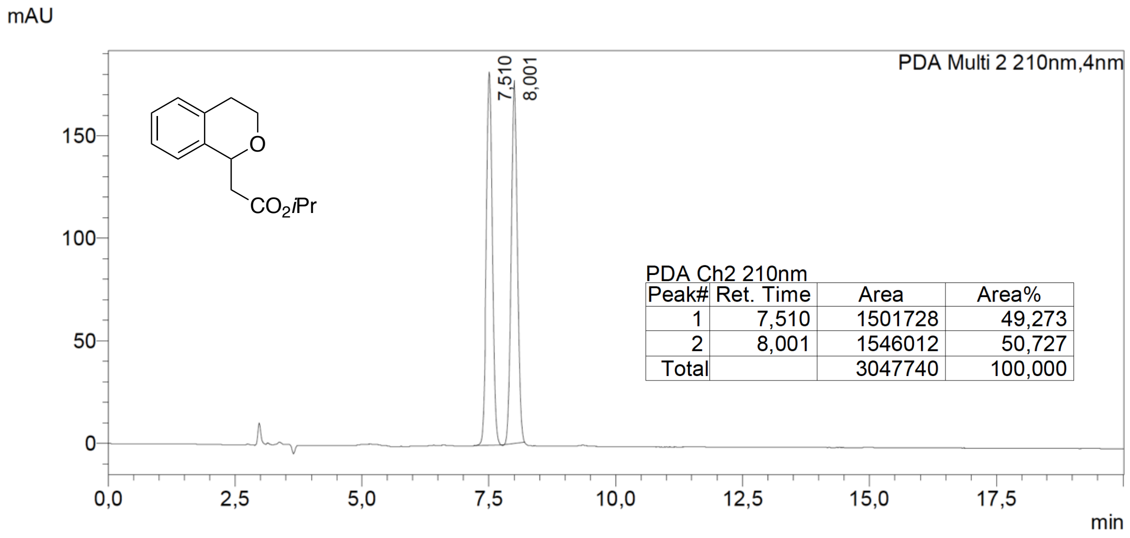

Peak#	Ret. Time	Area	Area%
1	7,510	1501728	49,273
2	8,001	1546012	50,727
Total		3047740	100,000

Figure S91. HPLC trace of **10a** obtained in the reaction catalyzed by (*R*,*R*)-(*P*,*P*)-*trans*-**1b**.

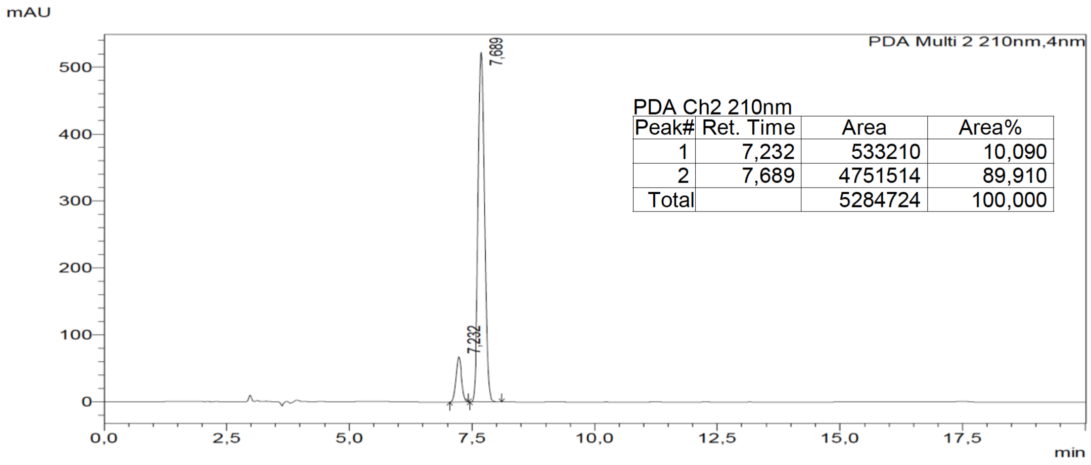

Peak#	Ret. Time	Area	Area%
1	7,232	533210	10,090
2	7,689	4751514	89,910
Total		5284724	100,000

Figure S92. HPLC trace of **10a** obtained in the reaction catalyzed by (*R*,*R*)-(*P*,*P*)-*cis*-**1b**.

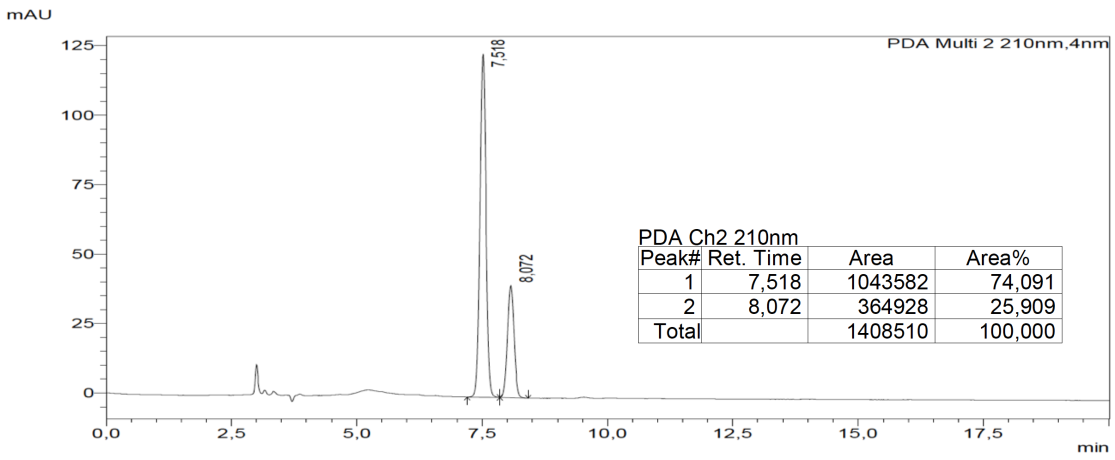

Peak#	Ret. Time	Area	Area%
1	7,518	1043582	74,091
2	8,072	364928	25,909
Total		1408510	100,000

Figure S93. HPLC trace of **10a** obtained in the reaction catalyzed by (*R*,*R*)-(*M*,*M*)-*cis*-**1b**.

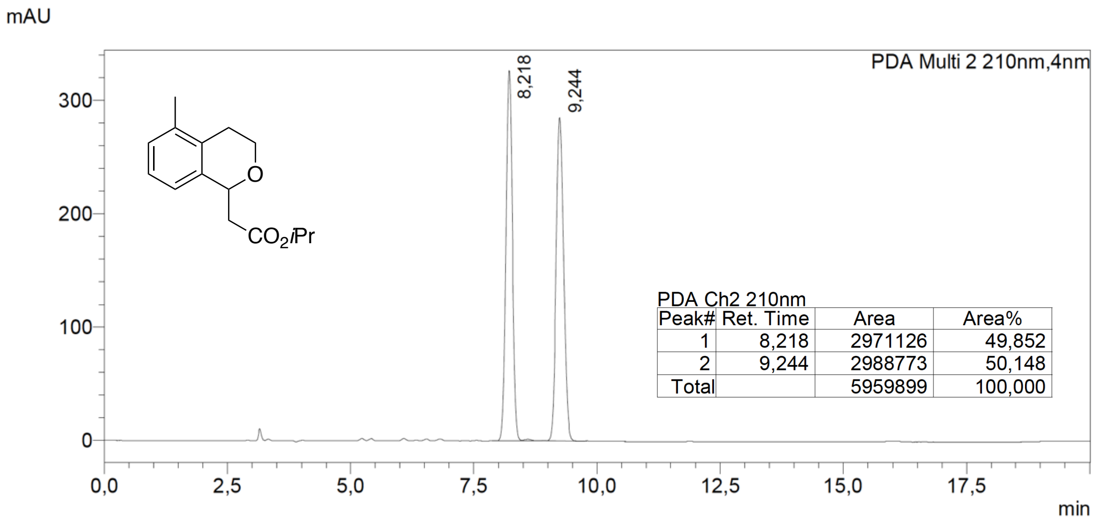

PDA Ch2 210nm

Peak#	Ret. Time	Area	Area%
1	8,218	2971126	49,852
2	9,244	2988773	50,148
Total		5959899	100,000

Figure S94. HPLC trace of **10b** obtained in the reaction catalyzed by (*R*,*R*)-(*P*,*P*)-*trans*-**1b**.

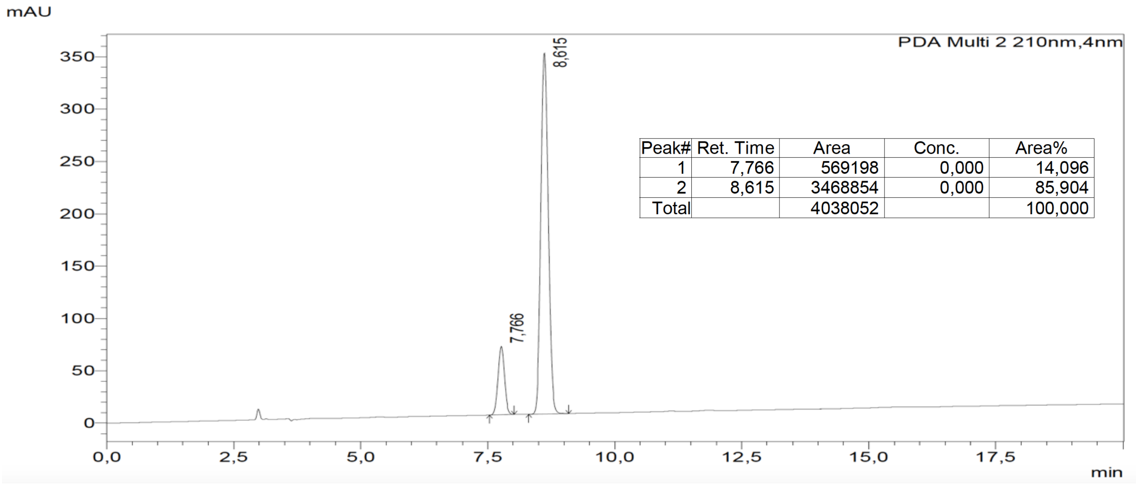

Peak#	Ret. Time	Area	Conc.	Area%
1	7,766	569198	0,000	14,096
2	8,615	3468854	0,000	85,904
Total		4038052		100,000

Figure S95. HPLC trace of **10b** obtained in the reaction catalyzed by (*R*,*R*)-(*P*,*P*)-*cis*-**1b**.

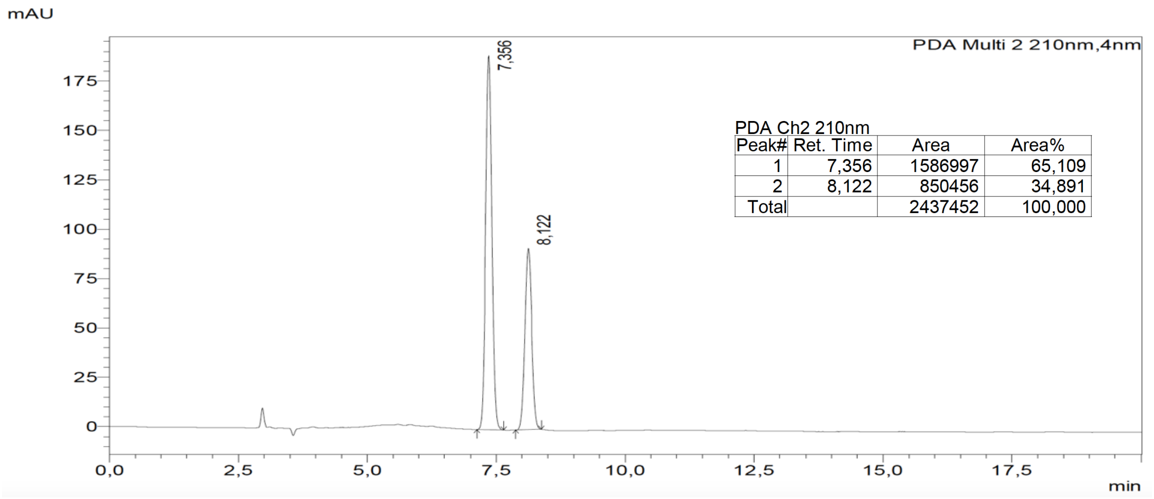

PDA Ch2 210nm

Peak#	Ret. Time	Area	Area%
1	7,356	1586997	65,109
2	8,122	850456	34,891
Total		2437452	100,000

Figure S96. HPLC trace of **10b** obtained in the reaction catalyzed by (*R*,*R*)-(*M*,*M*)-*cis*-**1b**.

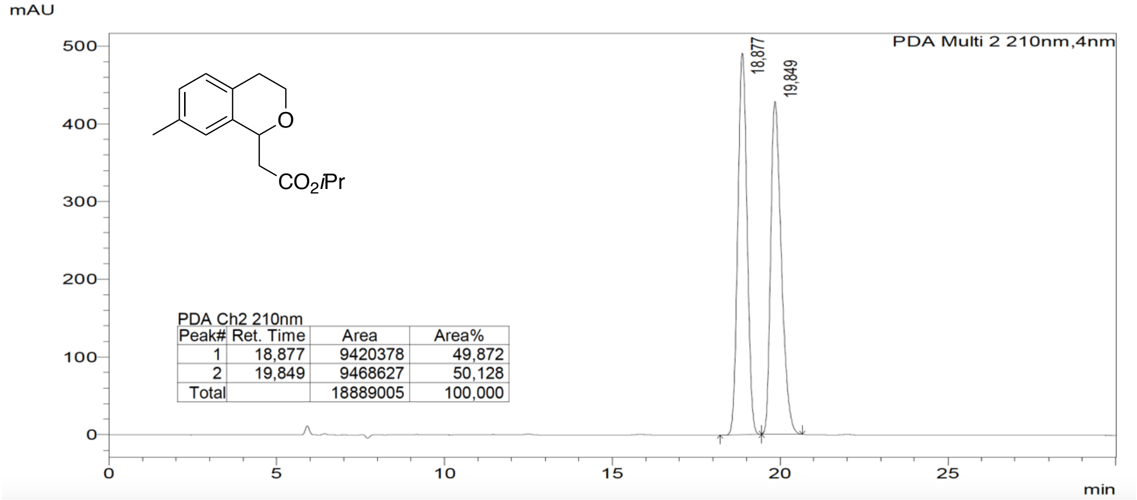

PDA Ch2 210nm

Peak#	Ret. Time	Area	Area%
1	18,877	9420378	49,872
2	19,849	9468627	50,128
Total		18889005	100,000

Figure S97. HPLC trace of **10c** obtained in the reaction catalyzed by (*R*,*R*)-(*P*,*P*)-*trans*-**1b**.

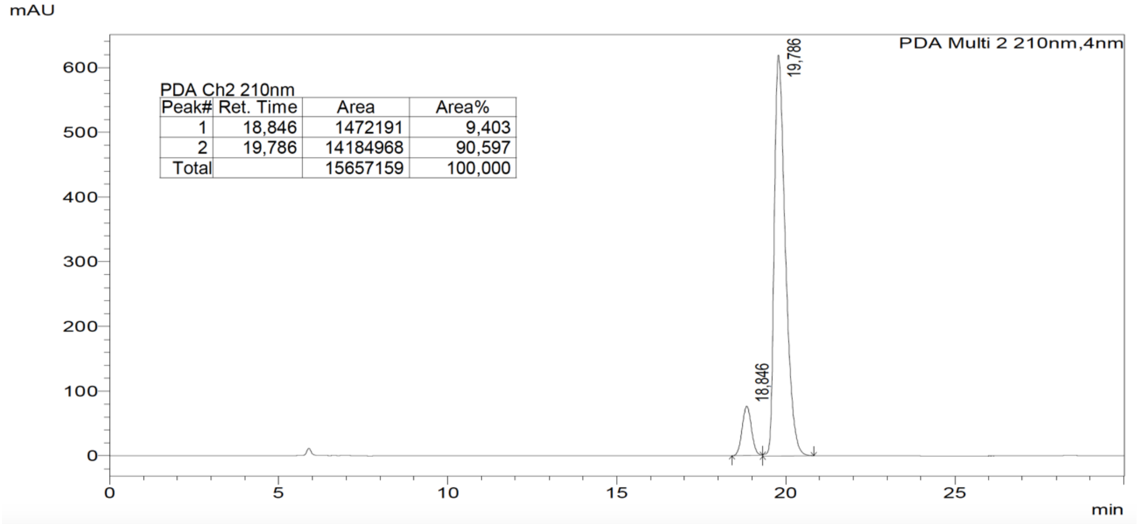

PDA Ch2 210nm

Peak#	Ret. Time	Area	Area%
1	18,846	1472191	9,403
2	19,786	14184968	90,597
Total		15657159	100,000

Figure S98. HPLC trace of **10c** obtained in the reaction catalyzed by (*R*,*R*)-(*P*,*P*)-*cis*-**1b**.

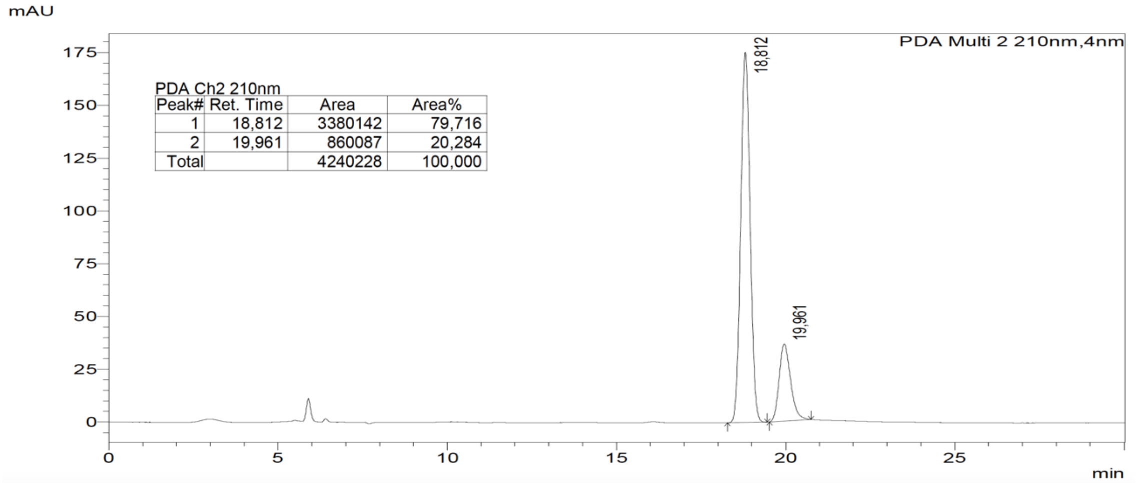

PDA Ch2 210nm

Peak#	Ret. Time	Area	Area%
1	18,812	3380142	79,716
2	19,961	860087	20,284
Total		4240228	100,000

Figure S99. HPLC trace of **10c** obtained in the reaction catalyzed by (*R*,*R*)-(*M*,*M*)-*cis*-**1b**.

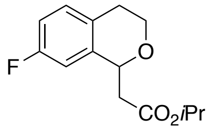

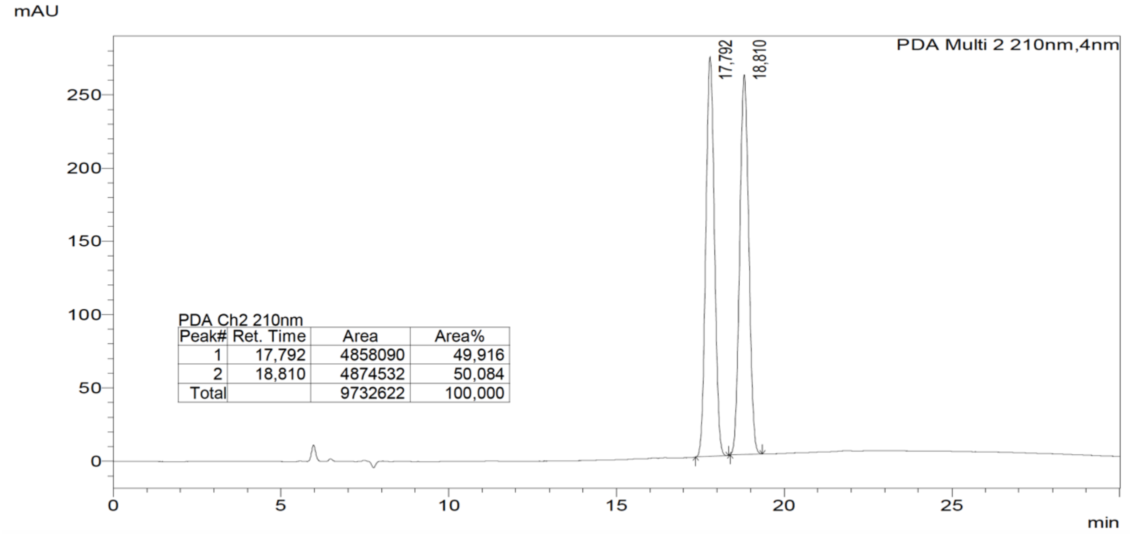

Figure S100. HPLC trace of **10d** obtained in the reaction catalyzed by (*R*,*R*)-(*P*,*P*)-*trans*-**1b**.

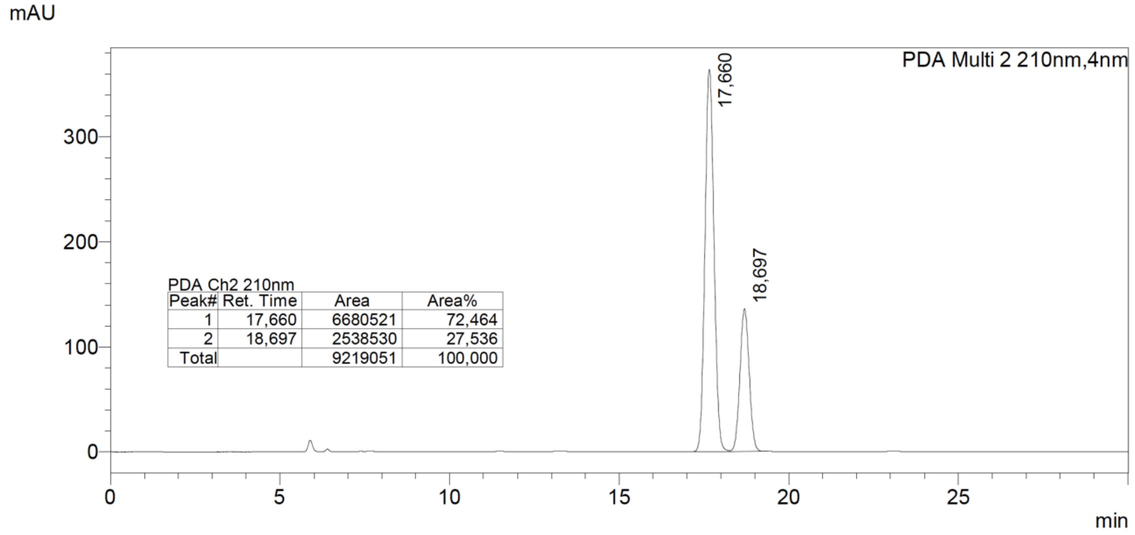

Figure S101. HPLC trace of **10d** obtained in the reaction catalyzed by (*R*,*R*)-(*P*,*P*)-*cis*-**1b**.

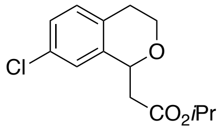

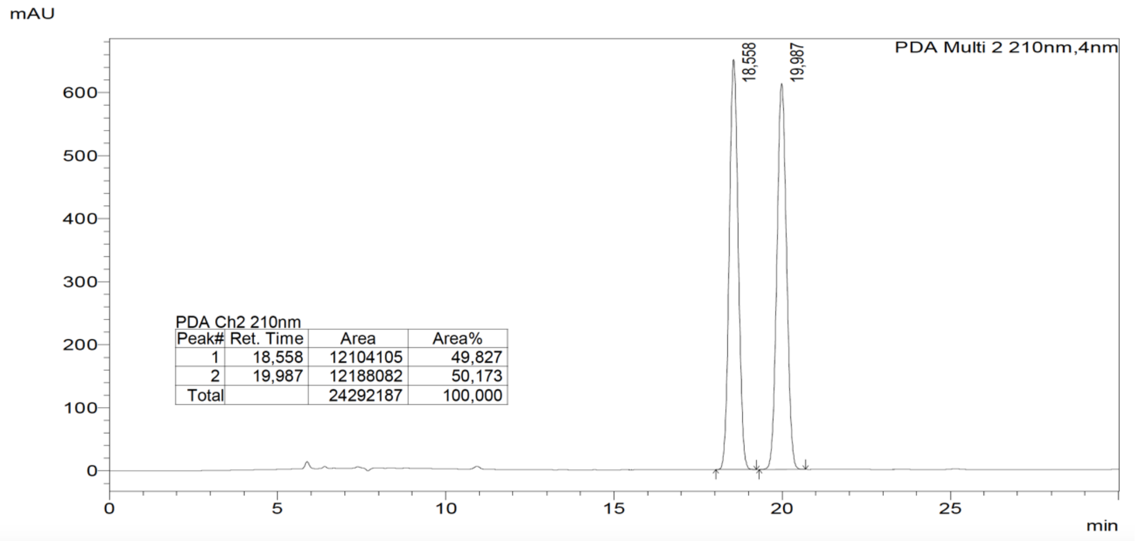

PDA Ch2 210nm

Peak#	Ret. Time	Area	Area%
1	18,558	12104105	49,827
2	19,987	12188082	50,173
Total		24292187	100,000

Figure S102. HPLC trace of **10e** obtained in the reaction catalyzed by (*R*,*R*)-(*P*,*P*)-*trans*-**1b**.

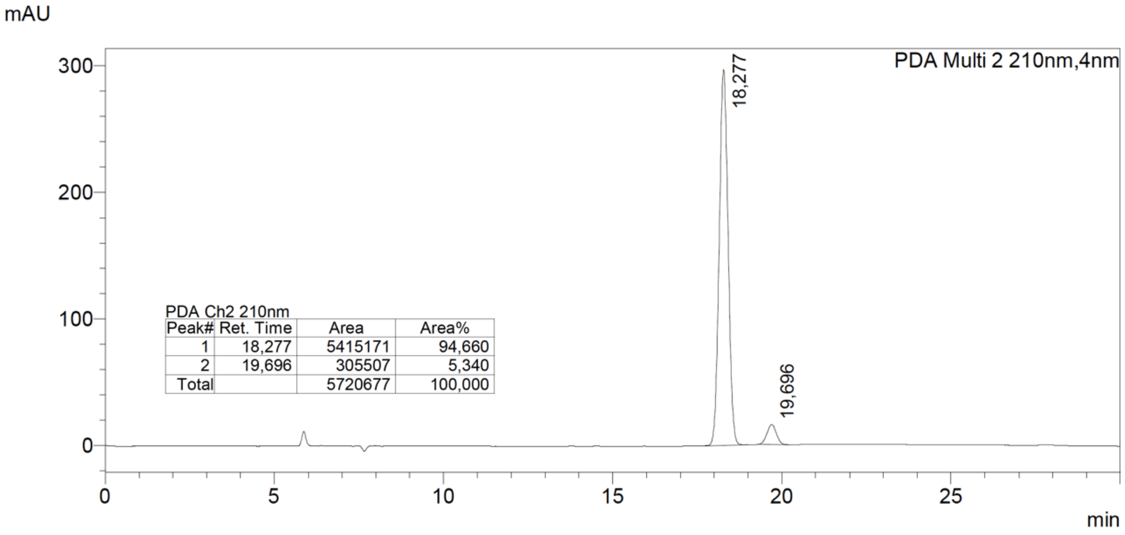

PDA Ch2 210nm

Peak#	Ret. Time	Area	Area%
1	18,277	5415171	94,660
2	19,696	305507	5,340
Total		5720677	100,000

Figure S103. HPLC trace of **10e** obtained in the reaction catalyzed by (*R*,*R*)-(*P*,*P*)-*cis*-**1b**.

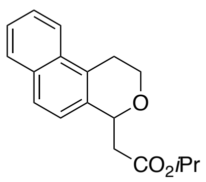

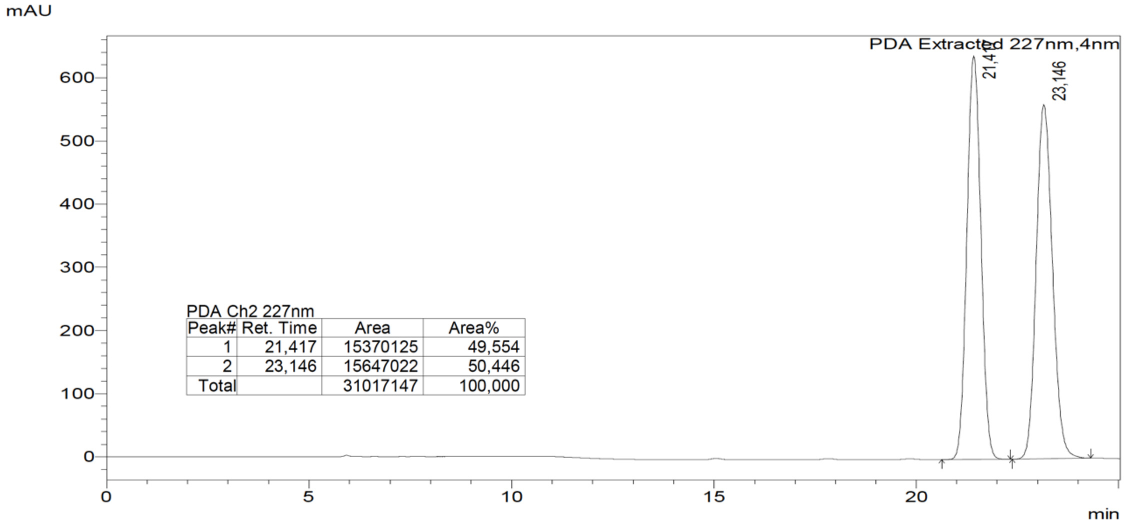

Figure S104. HPLC trace of **10f** obtained in the reaction catalyzed by (*R*,*R*)-(*P*,*P*)-*trans*-**1b**.

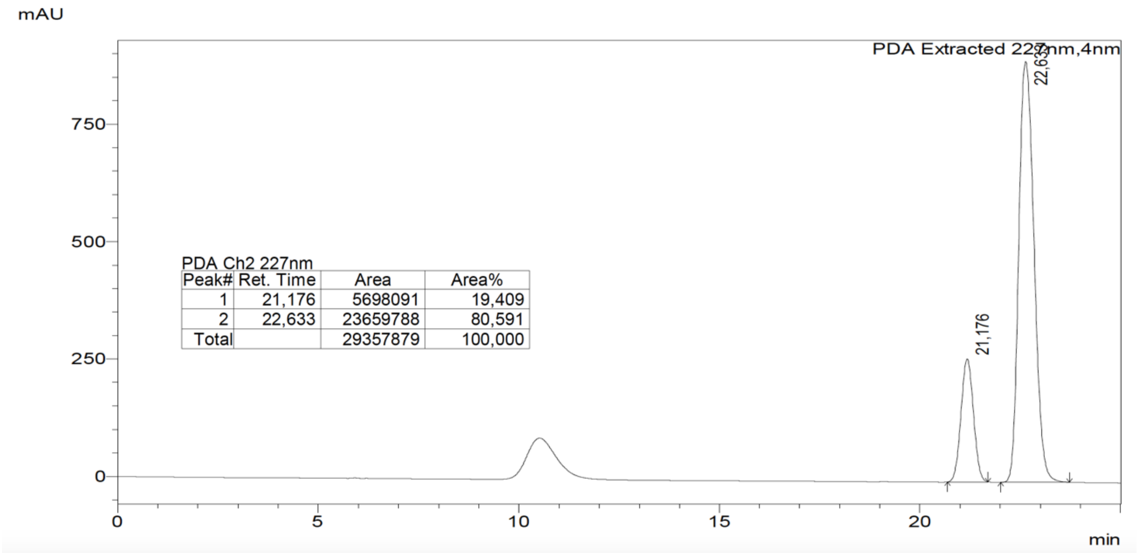

Figure S105. HPLC trace of **10f** obtained in the reaction catalyzed by (*R*,*R*)-(*P*,*P*)-*cis*-**1b**.
